# Antiviral activity and mechanism of purine morpholine nucleoside analogues incorporating a sulfonamide fragment

**DOI:** 10.1016/j.jare.2025.05.062

**Published:** 2025-06-01

**Authors:** Yuyuan Yang, Runjiang Song, Shaobo Wang, Guangcheng Zu, Baoan Song

**Affiliations:** State Key Laboratory of Green Pesticide, Center for R&D of Fine Chemicals of Guizhou University, Guiyang 550025, PR China

**Keywords:** Purine nucleoside analogues, Sulfonamide moiety, *Pepper mild mottle virus*, Antiviral activities, Mechanisms of action

## Abstract

**Introduction:**

*Pepper mild mottle virus* (PMMoV) poses an enormous threat to pepper production because of its high infectivity and soil persistence. Commercially available antiviral agents, ningnanmycin and ribavirin, have been restricted from widespread use due to their photosensitivity, water viscosity, and unsatisfactory efficacy.

**Objectives:**

In order to synthesize compounds with superior antiviral activities against PMMoV and clarify their antiviral mechanisms.

**Methods:**

A series of purine morpholine nucleoside analogues incorporating a sulfonamide moiety were successfully synthesized from D-glucosamine hydrochloride through a systematic multistage reaction involving condensation, acetylation, hydrolysis, sulfonation, bromination, and substitution. And activity-based protein profiling (ABPP) technology, Western blot, and microscale thermophoresis (MST) were combined to validate the target protein of **C1** acting on PMMoV. Subsequently, molecular docking, MD simulations, Western blot, and RT-qPCR were employed to identify the key amino acid sites for **C1** action. Next, co-immunoprecipitation mass spectrometry (Co-IP MS), RNA sequencing (RNA-seq), luciferase complementation assay (LCA), bimolecular fluorescence complementation (BiFC), and fluorescence recovery after photobleaching (FRAP) were applied to investigate the underlying mechanisms of infection diversity between GFP-PMMoV CP and GFP-PMMoV CP^Y13A^. Finally, the function of I3QHX5 in viral infection was further validated by virus-induced gene silencing (VIGS).

**Results:**

Thirty-eight purine morpholine nucleoside analogues incorporating a sulfonamide fragment with preferable inhibiting activities against PMMoV were designed and synthesized for the first time. ABPP technology, Western blot, and MST manifested that PMMoV CP was the target protein of **C1**. Molecular docking, MD simulations, RT-qPCR, and Western blot revealed that the tyrosine at position 13 (Tyr13) of PMMoV CP might be the key amino site for **C1** action. Co-IP MS, RNA-seq, LCA, BiFC, and FRAP indicated that the discrepancy in the liquid–liquid phase separation (LLPS) between PMMoV CP and PMMoV CP^Y13A^ with I3QHX5 might be the main cause of infection differences. Further VIGS demonstrated that silencing of I3QHX5 led to an attenuated accumulation of PMMoV.

**Conclusion:**

Our research revealed that purine morpholine nucleoside analogues containing a sulfonamide fragment possessed preferable inhibitory activities against PMMoV. Correlated mechanism studies indicated that **C1** could inhibit the formation of LLPS by specifically targeting Tyr13 of PMMoV CP, thus achieving the purpose of antiviral effects.

## Highlights

• Thirty-eight compounds of purine morpholine nucleoside analogues containing a sulfonamide moiety with favorable inhibitory activities against PMMoV were designed and synthesized.
• ABPP technology, western blot, and MST demonstrated that PMMoV CP was the target protein of C1. Molecular docking, MD simulations, confocal, Western blot, and RT-qPCR revealed that Y13A in PMMoV CP could severely inhibit virus infection.
• Co-IP MS, RNA-seq, LCA, and BiFC revealed that PMMoV CP could aggregate after interacting with I3QHX5, while PMMoV CP^Y13A^ could not under confocal. And the aggregates could be inhibited by applying of C1.
• Fusion, fission and FRAP demonstrated that the condensed material formed by CP and I3QHX5 had the properties of LLPS both *in vivo* and *in vitro*. Thus, PMMoV CP could promote viral infection, while PMMoV CP^Y13A^ could not.
• Further VIGS demonstrated that silencing of I3QHX5 leading to an attenuated accumulation of PMMoV CP.

## Graphical abstract

**Figure:**
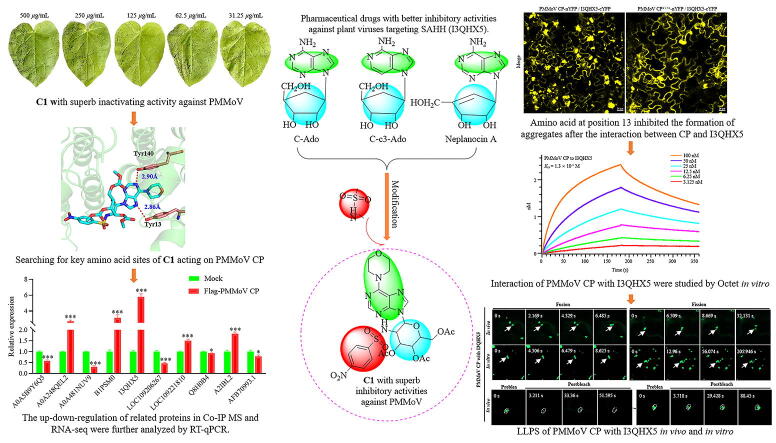

## Introduction

Chili peppers (*Capsicum* spp*.*), also known as bell peppers, are one of the most significant agricultural crops in the world, with a total global production of 752,000 tonnes and an overall market revenue of $4.1 billion in 2018 (https://www.researchandmarkets.com/reports/4701016/world-pepper-market-analysis -forecast). Rich in vitamins C, A, and E, and they are also a vital source of carotenoids as well as other essential nutrients such as fiber, potassium, iron, calcium, and folic acid [1,2], with properties of antioxidant, anticancer, anti-inflammatory, antimicrobial, and insecticidal [3,4]. Among all the categories of plant pathogens, viruses are the most destructive biological agents found in chili peppers [[5], [6], [7]]. Currently, 68 viruses have been reported in chili peppers [8], among which *pepper mild mottle virus* (PMMoV) poses a severe threat to the production of chili peppers due to its high infectivity and soil persistence [7]. The incidence of PMMoV has been reported to be as high as 95 %, which in turn leads to yield losses of 75–95 %, thus taking a severe threat to chili peppers [2]. Recent studies have demonstrated that PMMoV is correlated with clinical symptoms such as fever, itching, and abdominal pain, indicating that it might render a substantial risk to human health [9,10]. Commercially available antiviral agents, ningnanmycin and ribavirin, have been restricted from widespread use because of their photosensitivity, water viscosity and unsatisfactory efficacy [11]. Therefore, developing an efficient and eco-friendly antiviral agent and elucidating its mechanism of action has become our top priority.

Purine nucleoside derivatives harbor extensive antiviral activities in medicine, such as for *herpes simplex virus* (HSV), *varicella-zoster virus* (VZV), *human cytomegalovirus* (HCMV), *canine distemper virus* (CDV), *hepatitis B virus* (HBV), *human immunodeficiency virus* (HIV) and so on [[12], [13], [14], [15], [16]]. However, fewer studies have been conducted as plant antiviral agents, most purine nucleoside analogs from pharmaceuticals are used directly to screen plant viruses, such as *tobacco mosaic virus* (TMV), *potato virus X* (PVX), *cucumber mosaic virus* (CMV), and it has been reported that they might target *S*-adenosyl homocysteine hydrolase (SAHH) [[17], [18], [19], [20], [21]]. The sulfonamide skeleton plays a crucial role in the development of antiviral agents and is regarded as a “molecular heterozygote” capable of forming hydrogen bonds and interacting with the unipolar environment in proteins [[22], [23], [24], [25], [26], [27], [28]]. Morpholine derivatives have a wide range of biological activities such as insecticides, fungicides, herbicides, and antivirals [29]. In this work, a series of purine morpholine nucleoside analogs containing a sulfonamide fragment were designed and synthesized by introducing sulfonamide and morpholine scaffolds into the purine ring and modifying both the sugar and purine rings for the first time (see Scheme 1 in Supporting Information for details).

In this study, thirty-seven purine morpholine nucleoside analogues **C1**, **C3-C38** containing a sulfonamide fragment with favorable activities against PMMoV were designed and synthesized. Based on the preferable inactivating activity of compound **C1**, small molecule probe **C2** was then synthesized. Its EC_50_ of inactivating activities against PMMoV was 40.7 µg/mL, which was comparable to **C1** (37.0 µg/mL) and superior to the control agent ningnanmycin (67.3 µg/mL), indicating that **C2** could serve as a small molecule probe to capture target proteins. Activity-based protein profiling (ABPP) technology, Western blot and microscale thermophoresis (MST) manifested that PMMoV CP was the target protein of **C1**. The results of molecular docking, MD simulations, RT-qPCR, and Western blot revealed that the tyrosine at position 13 (Tyr13) of PMMoV CP might be the key amino site for **C1** action. Further studies of co-immunoprecipitation mass spectrometry (Co-IP MS), RNA sequencing (RNA-seq), luciferase complementation assay (LCA), bimolecular fluorescence complementation (BiFC), fluorescence recovery after photobleaching (FRAP), and virus-induced gene silencing (VIGS) revealed that the discrepancies in the interaction between I3QHX5 and PMMoV CP, as well as PMMoV CP^Y13A^ might be the main cause of the differences in infection. To the best of our knowledge, this work not only stated **C1** could be employed as a novel antiviral agent but also interpreted its mechanism for the first time.

## Materials and methods

### *Materials*

Reagents and solvents involved in the experiments were purchased from commercial suppliers without ulteriorly processing. Antibody of PMMoV CP and plasmid of pCB-GFP-PMMoV were graciously provided by Prof. Fei Yan (Ningbo University, Zhejiang, China).

### *General procedures for the preparation of target compounds****C1****,****C3****-****C38***

Intermediates **a–e** and **h** were synthesized with reference to the methods depicted in literature [[30], [31], [32], [33], [34], [35]]. See Supporting Information for details.

To the mixture of morpholine (20.00 mg, 229.56 µmol) and triethylamine (69.69 mg, 688.69 µmol) in ethanol (10 mL), 2-(acetoxymethyl)-5-((substituted phenyl)sulfonamidyl)-6-(2-substituted-6-chloro-9*H*-purin-9-yl)tetrahydro-2*H*-pyran-3,4-dicarboxylic acid dihydrate **h** (183.65 µmol) dissolved in ethanol (5 mL) was slowly added, and the mixture was heated to reflux. The reaction was monitored by TLC (DCM/EA, 1:1 v/v) until full completion, and the solvent was removed by rotary evaporation. Then the residue was dissolved in DCM (20 mL) and washed with water (3 × 20 mL). The organic layer was dried over anhydrous MgSO_4_, filtered, and concentrated under reduced pressure. Finally, the products **C1**, **C3**-**C35** were purified by column chromatography (DCM/EA, 1:1 v/v) [36,37].

Randomly selected compounds **C1**, **C25**, and **C34** (146.82 mol) in anhydrous methanol (10 mL), and added methanol/sodium methoxide solution dropwise until the reaction mixture was basic (pH = 10). The mixture was agitated at rt until the reaction was complete. It was then concentrated under reduced pressure, and the residue was purified by column chromatography (DCM/MeOH, 10:1 v/v) to acquire compounds **C36**-**C38** [34,38,39].

### *General procedures for the preparation of target compound****C2***

Intermediate **i** was prepared following the method reported in the literature [36].

To the mixture of propargyl bromide (35.16 mg, 295.57 µmol) and potassium carbonate (30.64 mg, 221.68 µmol) in acetone (10 mL), 2-(acetoxymethyl)-5-((4-nitrophenyl)sulfonylamino)-6-(6-(piperazine-1-yl)-9*H*-purine-9-yl)tetrahydro-2*H*-pyrano-3,4-diacetyldiacetate (100 mg, 147.79 µmol) dissolved in acetone (5 mL) was dropwise added at rt. The mixture was concentrated under reduced pressure after the reaction was finished. And the residue was dissolved in DCM (20 mL) and washed with water (3 × 20 mL). Then the organic layer was dried over anhydrous MgSO_4_, filtered, and concentrated under reduced pressure. Finally, the residue was purified by column chromatography (DCM/EA, 1:1 v/v) to yield the target compound **C2**.

### *Antiviral activity assay*

Virions were isolated and extracted from leaves of *N. benthamiana* infected with PMMoV referring to the method recorded in the literature [40]. And the inhibiting activities of **C1**–**C38** against PMMoV were assessed with reference to the literature as well [26,34].

### *Microscale thermophoresis (MST)*

The binding affinities of compounds with PMMoV CP were analyzed by MST based on the procedures delineated in the literature [41,42].

### *Molecular modeling*

#### Homology modeling

Homology modeling was performed by Discovery Studio 4.5 to predict the three-dimension (3D) structure of PMMoV CP with TMV CP (PDB code 1EI7) [43,44] as a template. The model of PMMoV CP was subjected to energy minimization, and the rationality of protein structure was analyzed through Protein Health [45] in Discovery Studio 4.5 and MolProbity Ramachandran analysis in MolProbity (https://altmolprobity.biochem.duke.edu/) [46,47].

#### Molecular docking

Molecular docking was performed with reference to the methods described in the literature [48]. The 3D structures of compounds were intended by SYBYL-X2.0 followed by 2000-steps steepest descent minimization and 2000-sreps conjugate gradient minimization, respectively. PMMoV CP was obtained by above mentioned homology modeling, and the hydrogens of the PMMoV CP were added by Discovery Studio 4.5. Compounds were docked into the binding pocket of PMMoV CP through AutoDock 4.2 [49]. The grid was set to be 60 × 60 × 60 with a default spacing 0.375 Å. The genetic algorithm (GA) was used to perform 256 runs. Conformation with top rank was chosen as the representative for further analysis.

#### Molecular dynamics (MD) simulations

In order to obtain the stable binding mode of compounds with PMMoV CP, MD simulations were performed based on the docking results of ligands and proteins, respectively. AM1-BCC charge model in Antechamber module of Amber22 program was used to assign charges for ligand atoms [50,51]. The topology and coordinate files of the complex were built with the Leap module in Amber22 program. AMBER ff14SB force field for amino acid residues, and general AMBER force field (GAFF) for ligands [[52], [53], [54]], Cl^-^ or Na^+^ for neutralizing the system. All molecules were dissolved in a rectangular TIP3P aqueous cassette extending at least 10 Å in each direction from the solute [55,56]. The Cpptraj module in Amber22 was used to perform root mean square deviation (RMSD), root mean square fluctuation (RMSF) and radius of gyration (Rg) analysis for measuring the stability of the complex during the MD process.

#### Transient expression of agrobacterium tumefaciens

Infectious cDNA clones of GFP-PMMoV CP^Y13A^ and GFP-PMMoV CP^Y140A^ were constructed following the approach depicted in the literature [34]. And leaves with significant characteristics were collected and preserved at −80 ℃ for further Western blot [34,57] and RT-qPCR [34,58]. The sequences of primers used in the construction of plasmids and RT-qPCR were listed in Table S3 for details.

#### Co-immunoprecipitation mass spectrometry (Co-IP MS) and RNA-seq

Vector construction and material preparation

The Flag-tagged vector was digested with KpnI and AscI, then fusion proteins of Flag-PMMoV CP and Flag-PMMoV CP^Y13A^ were constructed through homologous recombination. Western blot was performed to determine the expression of proteins at 24, 48 and 72 h of post inoculation (hpi), and the time point with the highest expression was selected for further Co-IP MS and RNA-seq.

#### Luciferase complementation assay (LCA) and bimolecular fluorescence complementation (BiFC)

Designed primers to construct vectors of LCA and BiFC via homologous recombination. Extracted plasmids and waited for sequencing confirmation before transforming them into GV3101 (pU19). *A. tumefaciens* transient transformation solution (200 μM acetosyringone, 10 mM MgCl_2_, and 10 mM MES, pH = 5.6) was prepared to resuspend the organisms with a final OD_600_ of 1.0, then mixed them with equal OD_600_ and volume before being infiltrated into *N. benthamiana* of the same growth stage. Interactions of prey proteins of A0A248QEL2, B1PSM0, I3QHX5, LOC109221810, and A2IBL2 with bait proteins of PMMoV CP and PMMoV CP^Y13A^ were recorded by UV or confocal at 48–72 hpi. The primers involved in LCA and BiFC were listed in Table S3.

#### Virus-induced gene silencing (VIGS)

pTRV2-I3QHX5, pTRV2-PDS, and pTRV2 with pTRV1 in equal OD_600_ (OD_600_ = 0.2) and equal volume were co-infiltrated into *N. benthamiana* with 3–5 leaves. When the positive control (TRV:PDS) exhibited the symptom of bleaching at 10 dpi, RT-qPCR was used to detect the silencing efficiency of I3QHX5. Concurrently, GFP-PMMoV of OD_600_ = 0.1 was infiltrated. The expression levels of PMMoV CP and CP mRNA in TRV:00 and TRV:I3QHX5 infiltrated with GFP-PMMoV at 8 dpi were detected via Western blot and RT-qPCR. The primers involved in VIGS were listed in Table S3.

## Results

### *Design and synthesis of target compounds****C1****,****C3-C38***

**Fig. 1:**
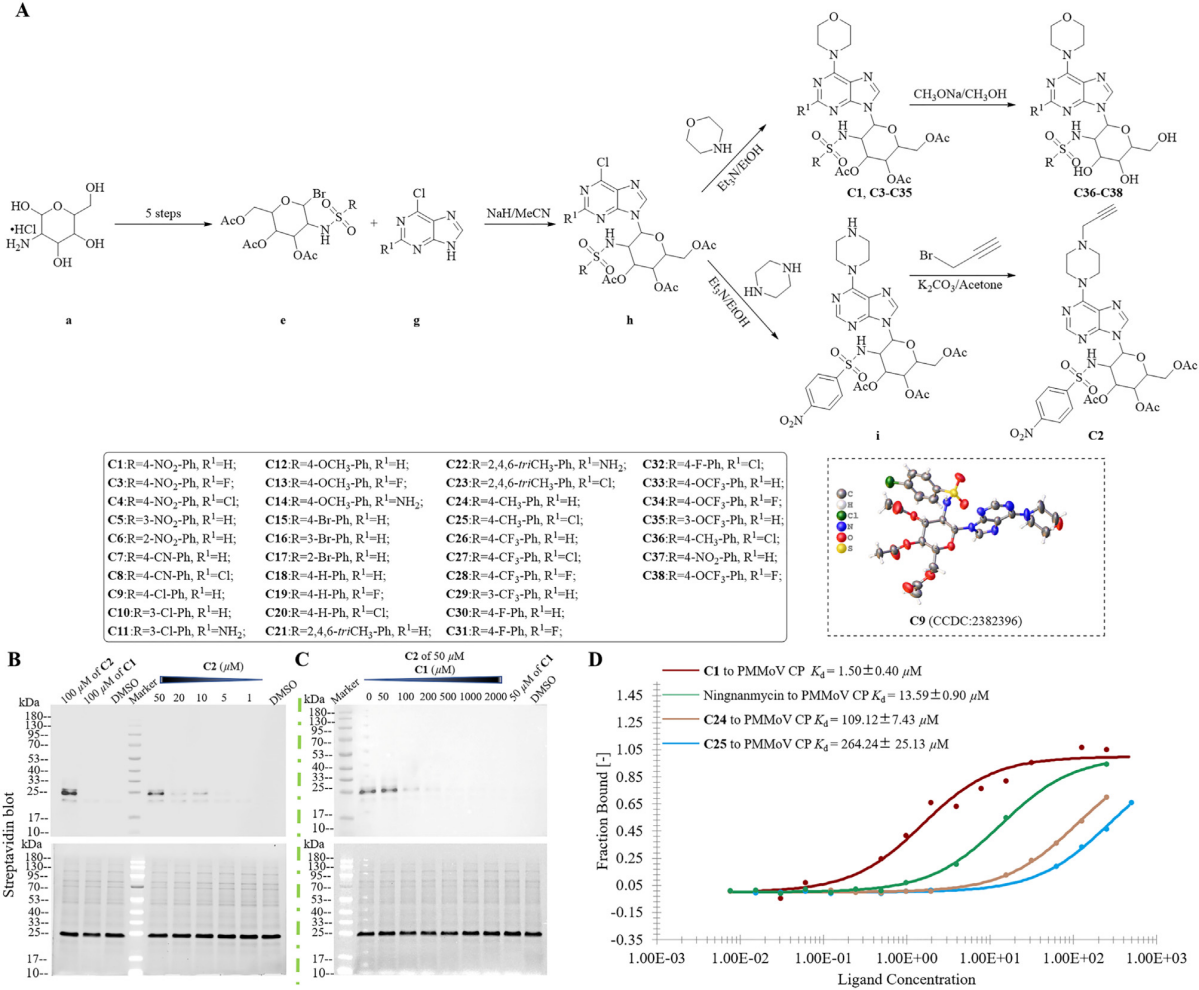
Determination of the target protein for **C1** action. (A) Design, synthesis of target compounds **C1**-**C38**, and single-crystal structure of **C9**. (B) Concentration-dependent experiments between **C2** and PMMoV CP. (C) Competitive inhibition assays of **C2** and **C1** against PMMoV CP. (D) Binding affinities of **C1**, **C24**, **C25**, and ningnanmycin with PMMoV CP.

A series of purine morpholine nucleoside analogues **C1**, **C3**-**C38** containing a sulfonamide moiety were designed and synthesized through modification of both sugar and purine rings. The target compounds underwent comprehensive structural characterization via ^1^H NMR, ^13^C NMR, ^19^F NMR, and HRMS. Slow crystallization with EA and DCM at rt yielded a single crystal of **C9**, and X-ray diffraction was further used to confirm its fine structure. (See Fig. 1A and Supporting Information for more details).

### *Inhibitory activities of****C1****,****C3****-****C38****against PMMoV*

As demonstrated in Table 1, **C1**, **C10**, **C12**, **C19**, **C22**, **C31**, **C32**, and **C34** all exhibited favorable inhibitory activities against PMMoV, with EC_50_ values of curative, protective, and inactivating activities were 17.0, 86.9, 37.0 µg/mL; 22.2, 17.8, 16.3 µg/mL; 53.0, 134.4, 43.1 µg/mL; 36.7, 159.1, 25.5 µg/mL; 26.2, 38.0, 13.7 µg/mL; 29.6, 99.6, 50.0 µg/mL; 23.8, 28.7, 38.0 µg/mL; and 33.8, 53.8, 53.0 µg/mL; respectively, which were significantly superior to the control agent ningnanmycin (257.6, 285.4, 67.3 μg/mL). And the inhibitory activities of **C1** against PMMoV *in vivo* were displayed in Fig. S129.

Subsequently, **C1**, **C25**, and **C34** were randomly selected for deacetylation, and the bioassay results demonstrated that the deprotected compounds of **C36**-**C38** exhibited poor antiviral activities compared to those of **C1**, **C25**, and **C34**, which were consistent with our previous results [26,34].

In addition, the structure–activity relationship (SAR) of **C1**, **C3**-**C38** against PMMoV was analyzed based on the data presented in Table 1. (1) When substituent R was identical, the antiviral activities of purine morpholine nucleoside analogues with R^1^ = H were superior to those of the corresponding R^1^ = F, R^1^ = Cl, and R^1^ = NH_2_. e.g., **C1** (R = 4-NO_2_, R^1^ = H, 37.0 µg/mL) < **C4** (R = 4-NO_2_, R^1^ = Cl, 141.3 µg/mL) < **C3** (R = 4-NO_2_, R^1^ = F, 198.1 µg/mL), **C12** (R = 4-OCH_3_, R^1^ = H, 43.1 µg/mL) < **C13** (R = 4-OCH_3_, R^1^ = F, 458.1 µg/mL) < **C14** (R = 4-OCH_3_, R^1^ = NH_2_, 4507.1 µg/mL), **C21** (R = 2,4,6-*tri*CH_3_, R^1^ = H, 83.2 µg/mL) < **C23** (R = 2,4,6-*tri*CH_3_, R^1^ = Cl, 239.5 µg/mL), **C24** (R = 4-CH_3_, R^1^ = H, 83.2 µg/mL) < **C25** (R = 4-CH_3_, R^1^ = Cl, 239.5 µg/mL), **C30** (R = 4-F, R^1^ = H, 20.4 µg/mL) < **C32** (R = 4-F, R^1^ = Cl, 38.0 µg/mL) < **C31** (R = 4-F, R^1^ = F, 50.0 µg/mL); (2) The antiviral activities of purine morpholine nucleoside analogues with electron-withdrawing groups were better than those of the corresponding electron-donating groups, when R^1^ remained constant and R was a different substituent. For example, **C1** (R = 4-NO_2_, R^1^ = H, 37.0 µg/mL) < **C9** (R = 4-Cl, R^1^ = H, 103.4 µg/mL) < **C18** (R = 4-H, R^1^ = H, 134.1 µg/mL) < **C24** (R = 4-CH_3_, R^1^ = H, 144.8 µg/mL), **C30** (R = 4-F, R^1^ = H, 20.4 µg/mL) < **C9** (R = 4-Cl, R^1^ = H, 103.4 µg/mL) < **C15** (R = 4-Br, R^1^ = H, 593.0 µg/mL); (3) When the substituents of both R^1^ and R were identical, the antiviral activities of the *para*-substituted purine morpholine nucleoside analogues were superior to those of the corresponding *ortho*- and *meta*-substituents. e.g., **C1** (R = 4-NO_2_, R^1^ = H, 37.0 µg/mL) < **C6** (R = 2-NO_2_, R^1^ = H, 181.7 µg/mL) < **C5** (R = 3-NO_2_, R^1^ = H, 622.1 µg/mL), **C33** (R = 4-OCH_3_, R^1^ = H, 176.4 µg/mL) < **C35** (R = 3-OCH_3_, R^1^ = H, 457.3 µg/mL); (4) When the substituents at R^1^ and R were identical, the antiviral activities of the purine morpholine nucleoside analogues were superior to those of the corresponding deacetylated analogues. For example, **C1** (R = 4-NO_2_, R^1^ = H, 37.0 µg/mL) < **C37** (R = 4-NO_2_, R^1^ = H, 279.7 µg/mL), **C34** (R = 4-OCF_3_, R^1^ = F, 53.0 µg/mL) < **C38** (R = 4-OCF_3_, R^1^ = F, 420.9 µg/mL). In summary, introducing electron-withdrawing groups at the *para*-position of the benzene ring while maintaining an unmodified purine ring was favorable for enhancing antiviral activities. Additionally, preserving the acetylated structure was crucial, as full exposure of the hydroxyl group might reduce activity due to increased polarity.

**Table 1:** Antiviral activities of purine morpholine nucleoside analogues **C1**-**C38** incorporating a sulfonamide fragment against PMMoV.

**Compd.**	Curative activity^a^^,^^b^ (%)	EC_50_ of curative activity^a^^,^^b^ (µg/mL)	Protective activity^a^^,^^b^ (%)	EC_50_ of protective activity^a^^,^^b^ (µg/mL)	Inactivating activity^a^^,^^b^ (%)	EC_50_ of inactivating activity^a^^,^^b^ (µg/mL)
**C1**	73.7 ± 5.2	17.0 ± 3.9	64.3 ± 2.8	86.9 ± 8.8	76.4 ± 2.6	37.0 ± 4.4
**C2**	67.3 ± 4.3	66.6 ± 4.5	65.9 ± 3.0	64.5 ± 4.5	67.4 ± 3.9	40.7 ± 2.3
**C3**	67.0 ± 3.6	11.5 ± 4.4	57.5 ± 4.3	387.8 ± 16.6	68.9 ± 4.9	198.1 ± 16.1
**C4**	61.2 ± 3.7	219.1 ± 9.0	52.6 ± 2.0	474.9 ± 15.8	64.1 ± 3.5	141.3 ± 12.1
**C5**	60.3 ± 2.5	63.8 ± 5.3	43.3 ± 2.3	2285.0 ± 24.8	47.5 ± 1.4	622.1 ± 14.7
**C6**	67.0 ± 4.9	131.7 ± 9.4	52.0 ± 2.3	357.9 ± 12.3	60.8 ± 1.9	181.7 ± 7.8
**C7**	55.8 ± 1.3	280.7 ± 10.3	44.7 ± 1.4	793.2 ± 17.1	36.6 ± 3.2	2414.6 ± 17.2
**C8**	69.6 ± 1.3	64.6 ± 4.6	44.3 ± 1.8	1043.6 ± 19.4	56.6 ± 4.0	222.2 ± 4.8
**C9**	70.9 ± 4.9	33.3 ± 1.2	58.9 ± 4.0	267.3 ± 8.6	65.8 ± 3.9	103.4 ± 9.1
**C10**	62.1 ± 5.8	22.2 ± 3.4	67.0 ± 4.3	17.8 ± 2.1	60.5 ± 1.8	16.3 ± 1.9
**C11**	51.8 ± 1.0	371.9 ± 18.4	58.8 ± 3.1	224.7 ± 11.9	51.8 ± 2.1	497.1 ± 3.0
**C12**	63.5 ± 2.6	53.0 ± 2.1	65.6 ± 4.7	134.4 ± 7.4	71.5 ± 5.0	43.1 ± 1.1
**C13**	72.5 ± 2.6	20.9 ± 3.0	46.5 ± 3.4	596.2 ± 10.9	47.3 ± 2.7	458.1 ± 3.3
**C14**	53.6 ± 2.4	100.1 ± 8.9	38.3 ± 4.4	925.9 ± 11.4	38.5 ± 2.0	4507.1 ± 24.2
**C15**	65.6 ± 3.4	61.0 ± 5.4	62.5 ± 3.3	103.2 ± 6.4	47.7 ± 0.69	593.0 ± 13.7
**C16**	58.9 ± 4.2	48.5 ± 3.1	45.3 ± 2.5	663.8 ± 12.4	55.9 ± 1.5	171.4 ± 9.6
**C17**	70.5 ± 3.8	16.1 ± 2.7	61.0 ± 5.3	140.4 ± 9.5	59.9 ± 4.0	84.0 ± 2.8
**C18**	63.5 ± 2.5	24.7 ± 1.3	41.9 ± 2.9	994.5 ± 11.2	61.3 ± 2.8	134.1 ± 8.5
**C19**	69.5 ± 4.7	36.7 ± 3.7	59.8 ± 3.6	159.1 ± 11.8	69.7 ± 1.9	25.5 ± 4.0
**C20**	74.8 ± 4.8	30.1 ± 3.5	54.8 ± 2.8	292.3 ± 17.6	72.0 ± 4.2	28.5 ± 4.2
**C21**	66.9 ± 1.8	61.6 ± 5.3	60.7 ± 2.5	74.0 ± 5.1	68.7 ± 4.5	83.2 ± 3.7
**C22**	67.6 ± 1.9	26.2 ± 1.3	61.2 ± 1.6	38.0 ± 3.5	61.3 ± 4.5	13.7 ± 1.6
**C23**	69.0 ± 3.5	11.3 ± 3.3	57.4 ± 1.6	144.7 ± 7.7	55.4 ± 2.6	239.5 ± 17.8
**C24**	72.5 ± 2.2	30.7 ± 1.2	40.8 ± 3.5	918.6 ± 10.2	65.1 ± 4.2	144.8 ± 4.7
**C25**	59.0 ± 3.5	167.1 ± 5.2	52.3 ± 1.1	345.1 ± 11.3	46.9 ± 1.8	572.7 ± 10.9
**C26**	67.6 ± 2.5	45.1 ± 4.7	44.5 ± 1.6	787.5 ± 12.8	45.5 ± 2.6	632.0 ± 15.2
**C27**	69.9 ± 3.6	51.1 ± 3.6	45.0 ± 3.4	534.0 ± 14.9	62.4 ± 4.7	184.4 ± 10.0
**C28**	69.4 ± 4.9	45.4 ± 3.0	42.9 ± 0.7	633.4 ± 13.6	44.0 ± 4.3	575.9 ± 14.9
**C29**	69.8 ± 4.3	64.8 ± 3.1	64.9 ± 4.7	88.4 ± 6.3	53.4 ± 0.8	352.8 ± 18.7
**C30**	67.9 ± 3.3	60.2 ± 3.1	58.9 ± 3.8	261.7 ± 10.3	61.7 ± 4.6	20.4 ± 4.9
**C31**	61.9 ± 3.3	29.6 ± 1.1	56.0 ± 1.9	99.6 ± 3.8	68.5 ± 1.7	50.0 ± 2.0
**C32**	68.0 ± 2.7	23.8 ± 5.5	68.9 ± 2.0	28.7 ± 4.4	67.9 ± 5.1	38.0 ± 4.4
**C33**	71.5 ± 4.4	32.0 ± 1.0	53.0 ± 3.7	305.4 ± 12.2	54.4 ± 3.3	176.4 ± 11.3
**C34**	67.4 ± 2.0	33.8 ± 4.6	65.6 ± 4.5	53.8 ± 4.8	72.7 ± 2.0	53.0 ± 2.2
**C35**	67.9 ± 3.3	17.0 ± 1.4	68.9 ± 2.0	33.9 ± 0.5	49.1 ± 1.6	457.3 ± 10.2
**C36**	57.3 ± 1.1	283.1 ± 10.5	52.2 ± 0.8	561.7 ± 11.1	50.6 ± 3.6	313.0 ± 7.1
**C37**	36.2 ± 4.6	1998.2 ± 21.8	50.6 ± 2.1	343.7 ± 15.8	57.8 ± 3.4	279.7 ± 13.9
**C38**	51.54 ± 0.3	441.9 ± 12.6	46.5 ± 1.0	473.8 ± 27.7	51.6 ± 0.5	420.9 ± 7.4
Ningnanmycin^c^	54.5 ± 3.3	257.6 ± 7.6	60.8 ± 3.1	285.4 ± 5.3	71.2 ± 6.1	67.3 ± 3.5

a Average of three replicates.

b The ± values represent standard deviation.

c Ningnanmycin was used as a positive control.

### *Searching for the target protein of****C1****acted on PMMoV*

As illustrated in Table 1, **C1**, **C10**, **C12**, **C19**, **C22**, **C31**, **C32**, and **C34** all exhibited pleasant inhibitory activities against PMMoV, and the EC_50_ of the inactivating activity of **C1** (37.0 µg/mL) against PMMoV was superior to those of **C12** (43.1 µg/mL), **C31** (50.0 µg/mL), **C32** (38.0 µg/mL), and **C34** (53.0 µg/mL). Although the EC_50_ values for the inactivating activities against PMMoV of **C10** (16.3 µg/mL) and **C22** (13.7 µg/mL) were better than that of **C1**, the inactivating activity of **C1** (76.4 %) was better than those of **C10** (60.5 %) and **C22** (61.3) at 500 µg/mL. Based on the dual evaluation system of EC_50_ value and inactivating efficiency, **C1** was chosen as a representative to investigate the inactivating mechanism of series **C** acting on PMMoV.

ABPP technology utilized its unique chemical probe strategy to selectively capture target proteins and was regarded as a well-established approach for discovering target proteins. According to the superior inactivating activity of **C1** against PMMoV, the small molecule probe **C2** was designed and synthesized by introducing an alkyne group into purine nucleoside with piperazine as a bridging bond. As can be seen from Table 1, **C2** exhibited excellent inactivating activity against PMMoV, with an EC_50_ value of 40.7 µg/mL, which was comparable to **C1** (37.0 µg/mL) and superior to ningnanmycin (67.3 µg/mL). The aforementioned results indicated that **C2** not only retains the antiviral properties of the parent compound, but also exhibits promising potential as a capture probe for target protein.

From the results of Western blot (Fig. S132), it could be seen that at the identical concentration, **C2** was capable of capturing the protein with a molecular weight of 17 kDa, whereas neither **C1** nor DMSO exhibited such a capacity. Of the four open reading frames of PMMoV, only PMMoV CP corresponded to a size of 17.5 kDa, indicating that the target protein of **C1** acting on PMMoV was PMMoV CP.

### *Concentration dependent experiment of****C2****with PMMoV CP and competitive inhibition between****C2****and****C1****with PMMoV CP*

As illustrated in Fig. 1B, **C2** could capture PMMoV CP, while **C1** could not at the same concentration of 100 µM. Additionally, **C2** successfully labeled protein at concentrations of 10–50 µM, but failed to do so when the concentrations were reduced to 5–1 µM. These results collectively established that the interaction of **C2** with PMMoV CP occurred in a concentration-dependent manner.

As manifested in Fig. 1C, **C2** formed a distinct protein band with PMMoV CP in the absence of **C1**, while no corresponding protein band was observed in DMSO. The intensity of this protein band was gradually weakened with increasing concentrations of **C1**. These findings collectively demonstrated that **C1** competitively inhibited the interaction of **C2** with PMMoV CP.

### *Detection of binding affinities of compounds with PMMoV CP via MST*

**C1**, **C24**, and **C25** with good, moderate, and poor inactivating activities against PMMoV were chosen to evaluate their binding affinities with PMMoV CP via MST using ningnanmycin as a control agent. According to Fig. 1D, compound **C1** exhibited strong binding affinity to PMMoV CP, with a dissociation constant of 1.50 µM, which was better than ningnanmycin (*K*_d_ = 13.59 µM), **C24** (*K*_d_ = 109.12 µM), and **C25** (*K*_d_ = 267.24 µM).

### *Searching for the pivotal amino acid residues of****C1****acting on PMMoV CP via molecular modeling*

**Fig. 2:**
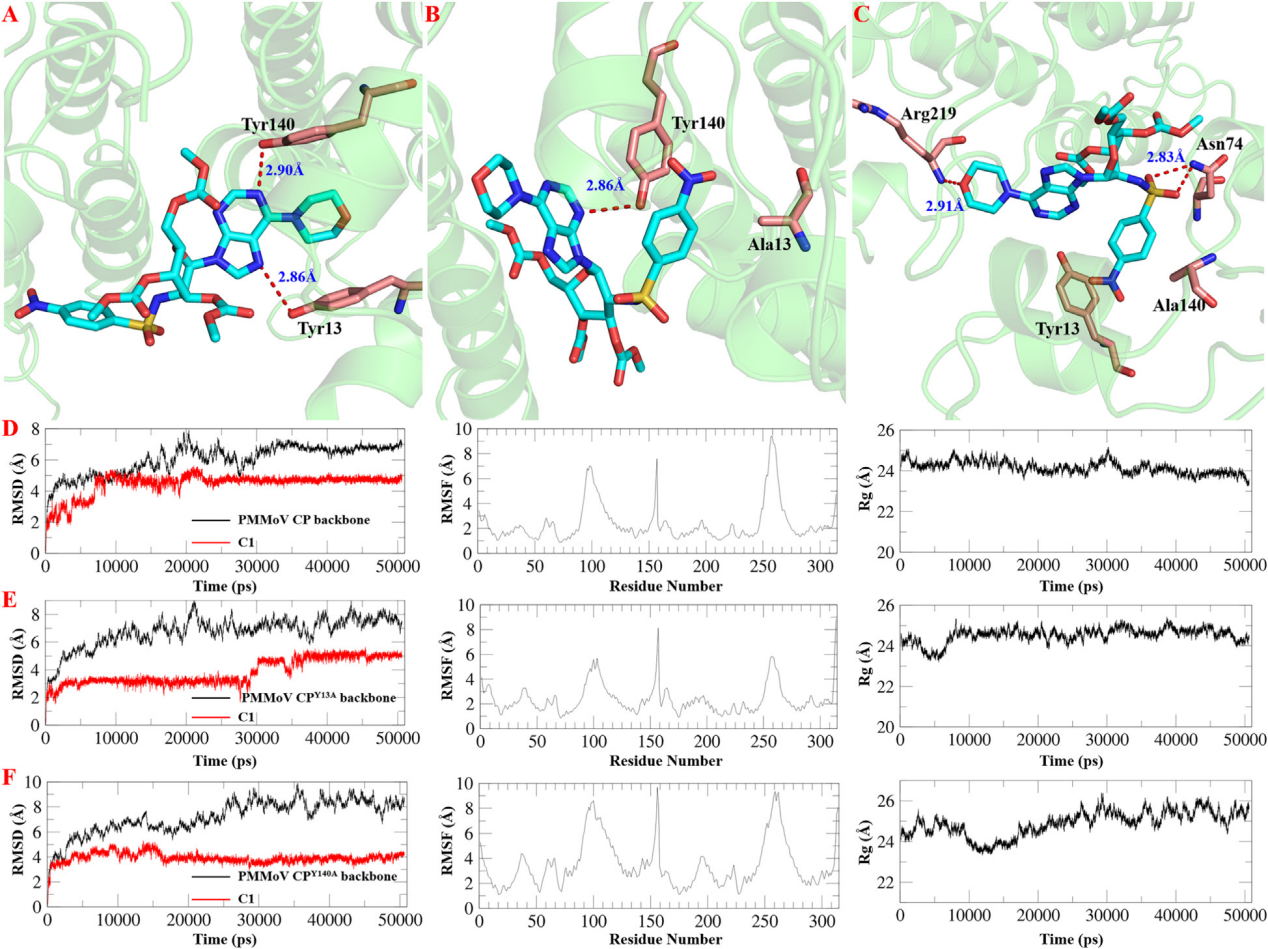
Searching for key amino acid sites of **C1** acting on PMMoV CP. (A, D) Molecular dynamics (MD) simulations and root mean square deviation (RMSD) between **C1** and PMMoV CP, root mean square fluctuation (RMSF) values and radius of gyration of gyration (Rg) of PMMoV CP. (B, E) MD simulations and RMSD between **C1** and PMMoV CP^Y13A^, RMSF and Rg of PMMoV CP^Y13A^. (C, F) MD simulations and RMSD between **C1** and PMMoV CP^Y140A^, RMSF and Rg of PMMoV CP^Y140A^.

As shown in Fig. 2D, RMSD of the system remained in an upward phase until 35,000 ps, indicating that **C1** and PMMoV CP were relatively unstable in the initial stage of MD simulations. However, as the simulation progressed, small molecule penetrated deeper into the protein pocket and formed additional hydrogen bonds, thus reaching a stable state for both. According to the binding mode obtained from equilibrated MD simulations (Fig. 2A), **C1** mainly interacted with the Tyr13 and Tyr140 of PMMoV CP through hydrogen bonding.

In order to investigate which of these two amino acid residues was more critical, PMMoV CP^Y13A^ and PMMoV CP^Y140A^ were subsequently constructed using Modeller and the MD simulations of their binding modes with **C1** were conducted via Amber22. From Fig. 2E-F, it could be seen that compared to PMMoV CP, PMMoV CP^Y13A^ and PMMoV CP^Y140A^ had larger fluctuations and they were basically in a dynamically stable state after 35000 ps. The fluctuation ranges of RMSF and Rg values of the proteins before and after the mutation were relatively minimal, indicating that the protein structures before and after the mutation were stabilized in the dynamic process. After reaching a relative equilibrium state, the binding energies (Table S2) of **C1** to PMMoV CP^Y13A^ and PMMoV CP^Y140A^ were −8.53 and −10.71 kcal/mol, respectively, both lower than that of **C1** to PMMoV CP (−24.08 kcal/mol), revealing that the binding stabilities of **C1** to PMMoV CP^Y13A^ and PMMoV CP^Y140A^ was not as stable as that of **C1** to PMMoV CP. And the binding energy of **C1** to PMMoV CP^Y13A^ was the weakest, indicating that Tyr13 plays a more critical role in stabilizing the interaction. The aforementioned findings implied that Tyr13 and Tyr140 of PMMoV CP might be the key binding sites for **C1**, with Tyr13 being potentially more critical. However, further experimental validation was required to confirm this hypothesis.

### *Difference in symptoms among GFP-PMMoV CP*^*Y13A*^*, GFP-PMMoV CP*^*Y140A*^*, and GFP-PMMoV in N. benthamiana*

**Fig. 3:**
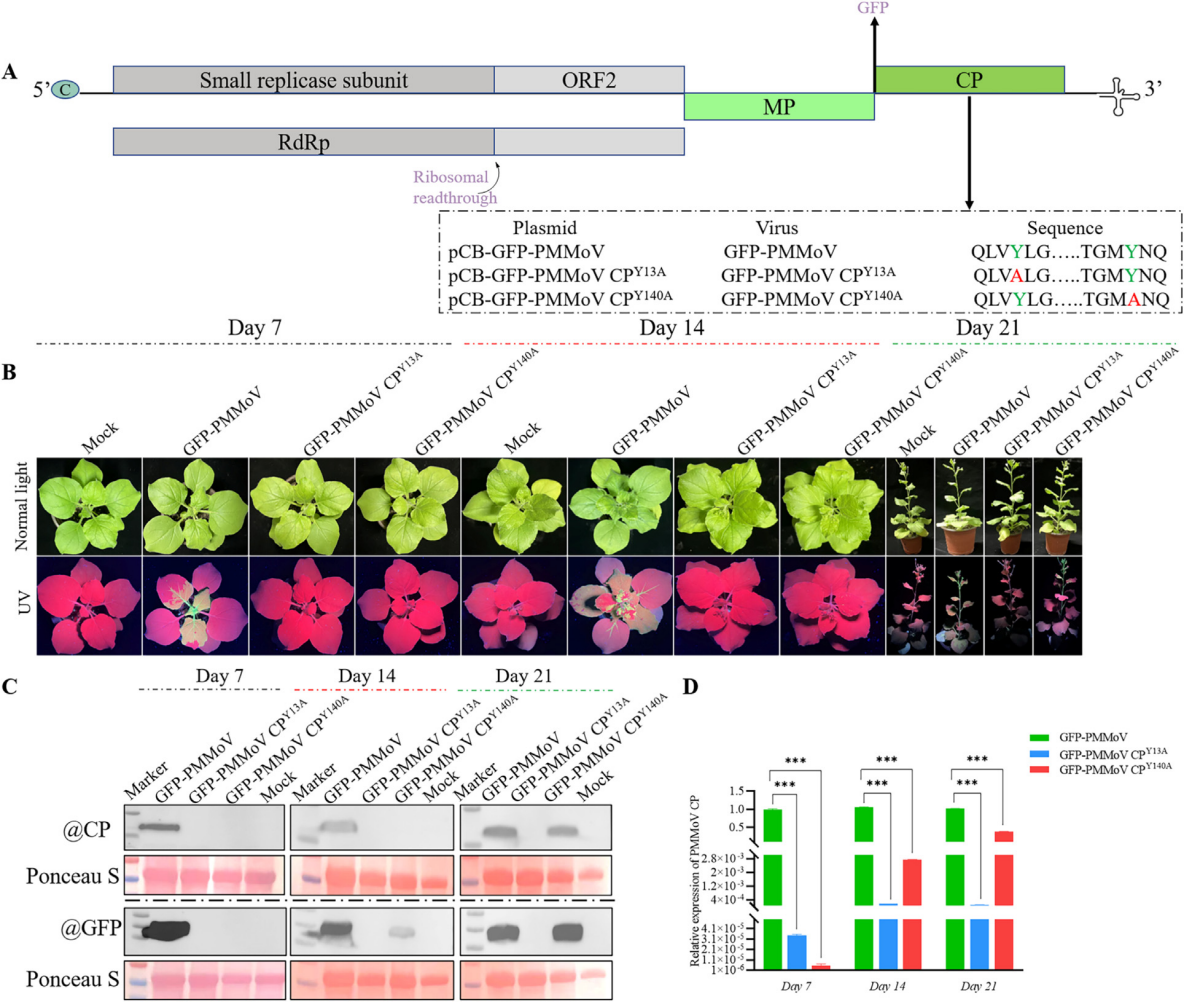
Determination and verification of key amino acid residues of **C1** acting on PMMoV CP. (A) Schematic diagram of pCB-GFP-PMMoV genome, wild-type and muted plasmids, viruses, and sequences were exhibited in the black border. (B) Phenotypic differences of *N. benthamiana* inoculated with wild-type and mutated GFP-PMMoV under normal light (upper panel) and UV illumination (upper panel). (C-D) The expression levels of PMMoV CP in the systemic leaves inoculated with wild-type and mutant GFP-PMMoV were analyzed by Western blot and quantitative RT-PCR (RT-qPCR) at 7, 14 and, 21 days of post inoculation (dpi). Statistical significance was determined by one-way ANOVA (IBM SPSS Statistics 27), with asterisks indicating *p*-values: *p* < 0.05, **p* < 0.01, ***p* < 0.001.

As exhibited in Fig. 3B, the green fluorescence of the inoculated leaves infiltrated with GFP-PMMoV changed from stronger to weaker at 7 dpi. Concurrently, widespread green fluorescence appeared in the systemic leaves. Compared with GFP-PMMoV, no green fluorescence was observed both in the systemic and infiltrated leaves inoculated with GFP-PMMoV CP^Y13A^ and GFP-PMMoV CP^Y140A^.

With the extension of time, both the systemic leaves and veins of *N. benthamiana* inoculated with GFP-PMMoV exhibited significant green fluorescence at 14 dpi. Compared with GFP-PMMoV, only trace green fluorescence was discovered in the systemic leaves inoculated with GFP-PMMoV CP^Y140A^, while there was no green fluorescence were detected in systemic leaves inoculated with GFP-PMMoV CP^Y13A^.

Eventually, more obvious green fluorescence was appeared in the whole strain of *N. benthamiana* inoculated with GFP-PMMoV at 21 dpi. And a large amount of green fluorescence was found both in the systemic leaves and veins after inoculation of GFP-PMMoV CP^Y140A^, whereas the green fluorescence of *N. benthamiana* injected with GFP-PMMoV CP^Y13A^ was hardly perceived.

### *Expression of CP and GFP by Western blot*

The results of Western blot (Fig. 3C) revealed that the expression levels of CP and GFP were higher in the systemic leaves of *N. benthamiana* inoculated with GFP-PMMoV at 7, 14 and 21 dpi. In comparison with GFP-PMMoV, neither CP nor GFP was detectable in the systemic leaves inoculated with GFP-PMMoV CP^Y13A^ and GFP-PMMoV CP^Y140A^ at 7 dpi. And the systemic leaves of *N. benthamiana* inoculated with GFP-PMMoV CP^Y13A^ and GFP-PMMoV CP^Y140A^ displayed almost no expression of CP, while GFP was slightly expressed with inoculation of GFP-PMMoV CP^Y140A^ at 14 dpi (Fig. 3C). Finally, both CP and GFP were highly expressed in systemic leaves of *N. benthamiana* inoculated with GFP-PMMoV^Y140A^, whereas neither CP nor GFP was expressed in systemic leaves inoculated with GFP-PMMoV^Y13A^ at 21 dpi (Fig. 3C).

### *Expression of CP gene via RT-qPCR*

RT-qPCR (Fig. 3D) demonstrated that the CP gene was unexpressed in the systemic leaves of *N. benthamiana* inoculated with GFP-PMMoV CP^Y13A^ and GFP-PMMoV CP^Y140A^, compared with those inoculated with GFP-PMMoV at 7 and 14 dpi. And the expression level of CP gene with inoculation of GFP-PMMoV CP^Y140A^ was approximately 26.0 % of GFP-PMMoV, whereas there was almost no expression of CP gene inoculated with GFP-PMMoV CP^Y13A^ at 21 dpi.

### *Looking for differential proteins that interacted with PMMoV CP and PMMoV CP*^*Y13A*^*through Co-IP MS and RNA-Seq*

**Fig. 4:**
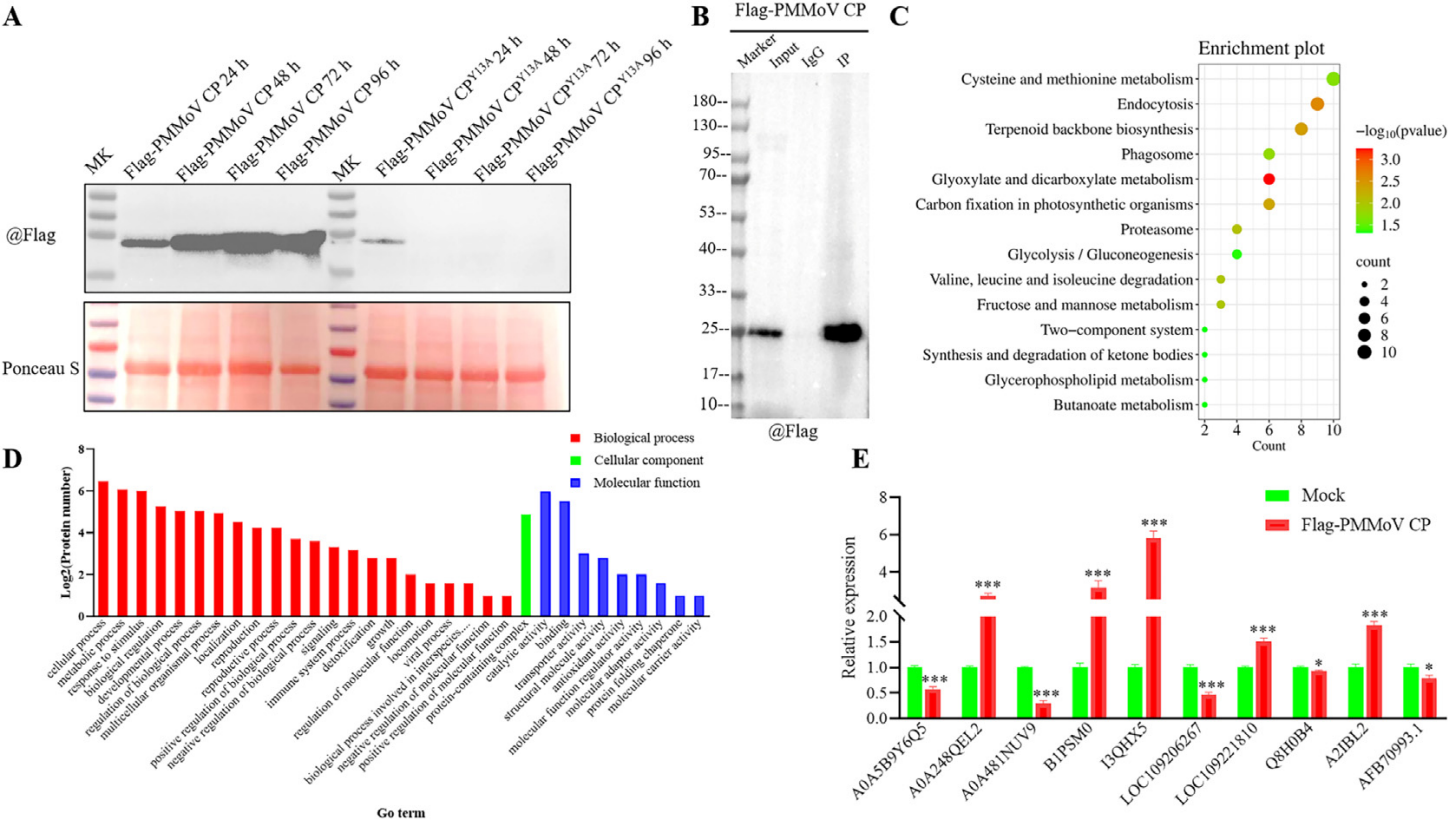
Identification of differentially expressed proteins (DEPs) interacting with PMMoV CP and PMMoV CP^Y13A^ via co-immunoprecipitation mass spectrometry (Co-IP MS) and RNA-seq. (A) The expression levels of Flag-PMMoV CP and Flag-PMMoV CP^Y13A^ were detected by Western blot at 24, 48, 72, and 96 h of post inoculation (hpi). (B) Protein enrichment analysis of different input, IgG and IP treatment groups. (C) Top enriched KEGG pathways from DEP screening. (D) Gene Ontology (GO) classification of DEPs across biological process, molecular function, and cellular component. (E) Candidate proteins in Co-IP MS and RNA-seq were analyzed by RT-qPCR. Statistical significance was determined by one-way ANOVA (IBM SPSS Statistics 27), with asterisks indicating *p*-values: *p* < 0.05, **p* < 0.01, ***p* < 0.001.

The plant samples of the third day with highest expression level of Flag-CP (Fig. 4A) were selected for Co-IP MS and RNA-seq. As can be seen from Fig. 4B, the enrichment of magnetic beads was qualified, and then mass spectrometry could be further performed. According to the mapping result of Co-IP MS and RNA-seq (Fig. S136), the differentially expressed proteins (DEPs) between CP and CP^Y13A^ were LOC109206267, LOC109221810 and AFB70993.1. Combining the top ranked DEPs in Co-IP MS with the analysis of Co-IP MS and RNA-seq, 10 DEPs (Table 2) were chosen and analyzed by RT-qPCR to validate the omics data. According to the results of RT-qPCR (Fig. 4D), the up-regulated proteins were mainly A0A248QEL2, B1PSM0, I3QHX5, LOC109221810, and A2IBL2, which were in agreement with the data of omics. Therefore, these 5 proteins were chosen for further study.

**Table 2:** List of differentially expressed proteins in Co-IP MS and RNA-seq.

No.	Protein ID	Protein Name	Organism	Length	Sig
1	A0A5B9Y6Q5	MLP-like protein 43	*N. benthamiana*	146 AA	up
2	A0A248QEL2	S-adenosylmethionine synthase	*N. benthamiana*	390 AA	up
3	A0A481NUV9	Ferredoxin-NADP(+) reductase (Fragment)	*N. benthamiana*	322 AA	up
4	B1PSM0	Methyltransferase	*N. benthamiana*	617 AA	up
5	I3QHX5	Adenosylhomocysteinase	*N. benthamiana*	485 AA	up
6	Q8H0B4	Mitogen-activated protein kinase	*N. benthamiana*	376 AA	up
7	A2IBL2	Histone H_2_B	*N. benthamiana*	147 AA	up
8	LOC109206267	Putrescine *N*-methyltransferase 2	*N. attenuata*	681 AA	up
9	LOC109221810	Putrescine *N*-methyltransferase 2	*N. attenuata*	566 AA	up
10	AFB70993.1	Ribulose-1,5-bisphosphate carboxylase/oxygenase large subunit	*N. attenuata*	477 AA	down

Enrichment analysis of the GO terms and KEGG pathways revealed that most of DEPs were mainly involved in primary and secondary metabolic processes. These findings further suggested that the infection of PMMoV significantly activated multiple proteins participating in metabolic pathways, thereby endowing plants with the ability to resist viruses.

### *Validation of differential proteins interacting with PMMoV CP and PMMoV CP*^*Y13A*^*via LCA*

**Fig. 5:**
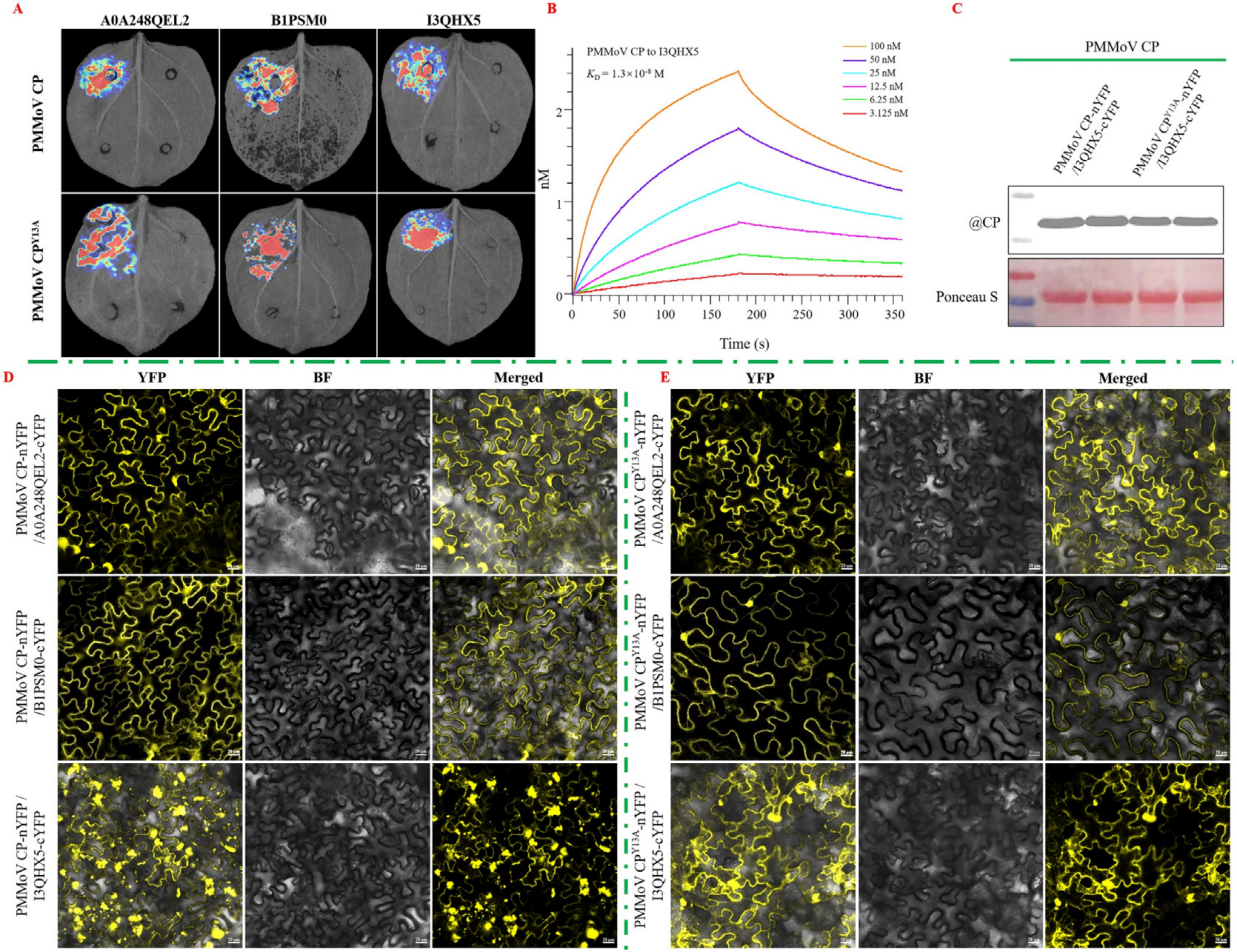
Differences in the interaction of PMMoV CP and PMMoV CP^Y13A^ with potential target proteins. (A, D) Analysis of the interactions between PMMoV CP and PMMoV CP^Y13A^ with A0A248QEL2, B1PSM0, and I3QHX5 by luciferase complementation assay (LCA) and bimolecular fluorescence complementary (BiFC). (B) Binding affinity of PMMoV CP with I3QHX5 *in vitro*. (C) The expression level of PMMoV CP in *N. benthamiana* co-inoculated with PMMoV CP-nYFP/I3QHX5-cYFP and PMMoV CP^Y13A^-nYFP/I3QHX5-cYFP at 2 dpi. Scale bars: 20 μm.

From the results of LCA in Fig. 5A, it can be seen that both PMMoV CP and PMMoV CP^Y13A^ interacted with A0A248QEL2, B1PSM0, I3QHX5, LOC109221810, and A2IBL2. (The LCA results of PMMoV CP and PMMoV CP^Y13A^ with LOC109221810 and A2IBL2 were shown in Fig. S137).

### *Confirmation of differential proteins interacting with PMMoV CP and PMMoV CP*^*Y13A*^*via BiFC*

**Fig. 6:**
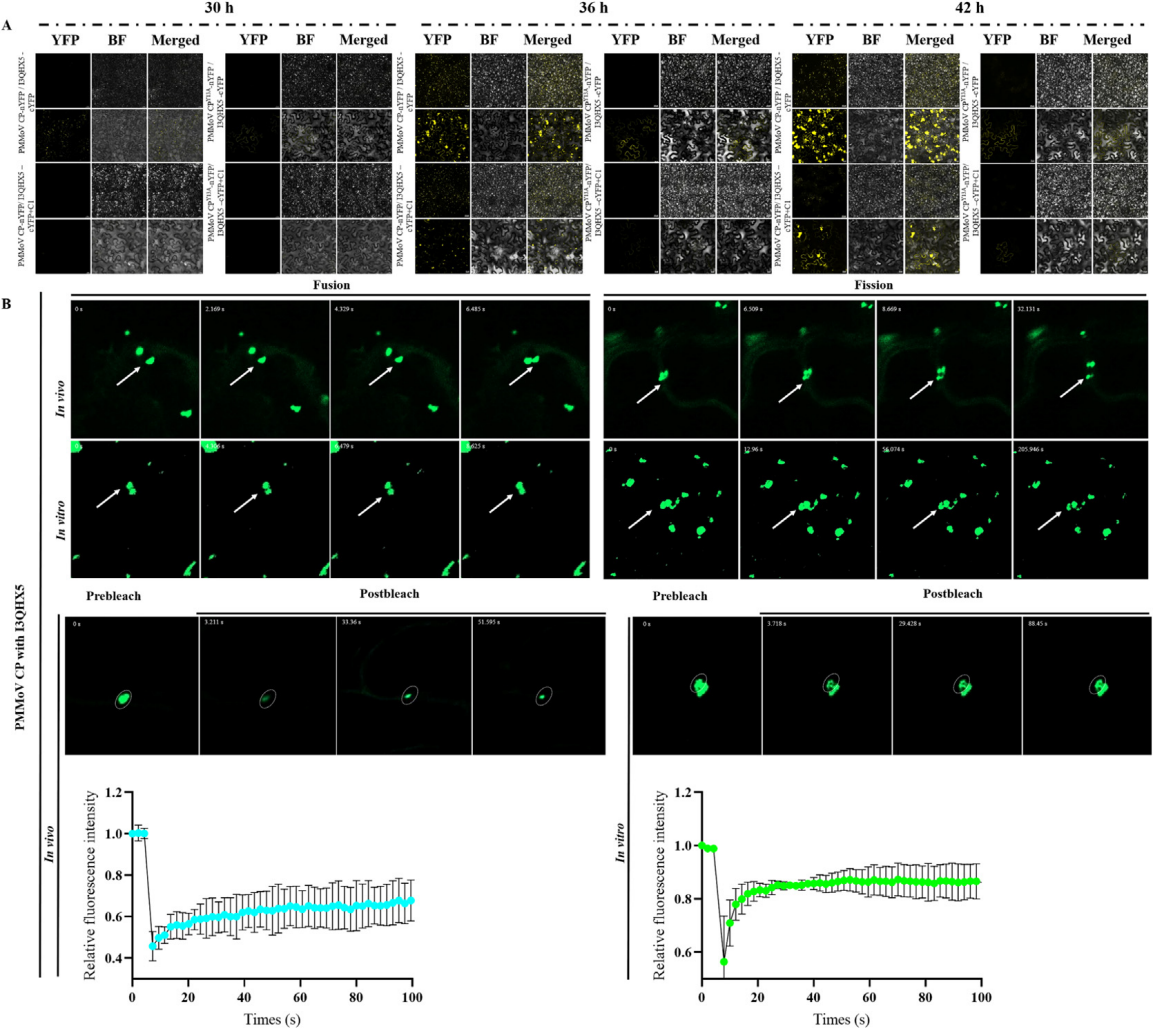
**C1** attenuated viral replication by mediating the liquid–liquid phase separation (LLPS) formed by PMMoV CP and I3QHX5. (A) Differences in the interaction of PMMoV CP and PMMoV CP^Y13A^ with I3QHX5 at 30, 36 and 42 hpi before and after application of **C1**. (B) Phase separation and fluorescence recovery after photobleaching (FRAP) of PMMoV CP with I3QHX5 *in vivo* and *in vitro*.

As can be seen from Fig. 5D-E, PMMoV CP and PMMoV CP^Y13A^ interacted with A0A248QEL2, B1PSM0, and I3QHX5, but not with A0A248QEL2 and LOC109221810 (The BiFC results of PMMoV CP and PMMoV CP^Y13A^ with LOC109221810 and A2IBL2 were exhibited in Fig. S138). Notably, there were differences in the interaction between PMMoV CP and PMMoV CP^Y13A^ with I3QHX5. Furthermore, the addition of **C1** also affected the interaction between PMMoV CP and PMMoV CP^Y13A^ with I3QHX5 at 30, 36 and 42 hpi (Fig. 6A).

### *Interaction of PMMoV CP with I3QHX5 in vitro*

The result of Octet (Fig. 5B) demonstrated that PMMoV CP had a nanomolar binding affinity to I3QHX5, with a binding constant of 1.3 × 10^-8^ M, which suggested that there was a strong interaction between PMMoV CP and I3QHX5 *in vitro*.

### *Liquid-liquid phase separation (LLPS) of PMMoV CP with I3QHX5*

BiFC results (Fig. 5D) illustrated that there were obvious fluorescent aggregation and droplet formation after the interaction of PMMoV CP with I3QHX5, and these droplets would undergo fusion and fission, after which they would relax into spherical shapes (Fig. 6). And with fluorescence bleaching, the fluorescence intensity of the aggregates decreased to less than 50 %, and after stopping fluorescence bleaching, the visible fluorescence intensity gradually recovered. These results indicated that the condensed material formed by CP and I3QHX5 has the properties of LLPS *in vivo*. Subsequently, PMMoV CP-GFP and I3QHX5-GFP fusion expression vectors were constructed and the proteins were purified, and the results of phase separation and FRAP were basically in accord with those *in vivo* (Fig. 6B).

Based on the above results, it was further demonstrated that CP had the properties of LLPS both *in vitro* and *in vivo* after interacting with I3QHX5.

### *Silencing of I3QHX5 attenuated the infection of GFP-PMMoV in N. benthamiana*

**Fig. 7:**
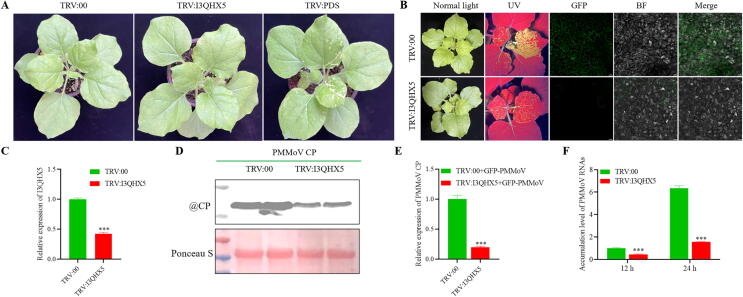
Silencing of I3QHX5 significantly attenuated systemic invasion of PMMoV. (A) Phenotypic differences of *N. benthamiana* inoculated with TRV:00, TRV:I3QHX5, and TRV:PDS at 10 dpi. (B) Symptomatic differences of TRV:00 and TRV:I3QHX5 inoculated with GFP-PMMoV under normal light, UV illumination and confocal at 8 dpi. (C) Relative expression of I3QHX5 in *N. benthamiana* inoculated with TRV:I3QHX5 and TRV:00 at 10 dpi. (D-E) The relative accumulation of PMMoV CP of *N. benthamiana* inoculated with TRV:I3QHX5 and TRV:00 via Western blot and RT-qPCR. (F) The accumulation level of PMMoV RNAs in protoplasts inoculated with TRV:I3QHX5 and TRV:00. Scale bars: 20 μm. Statistical significance was determined by one-way ANOVA (IBM SPSS Statistics 27), with asterisks indicating *p*-values: *p* < 0.05, **p* < 0.01, ***p* < 0.001.

**Fig. 8:**
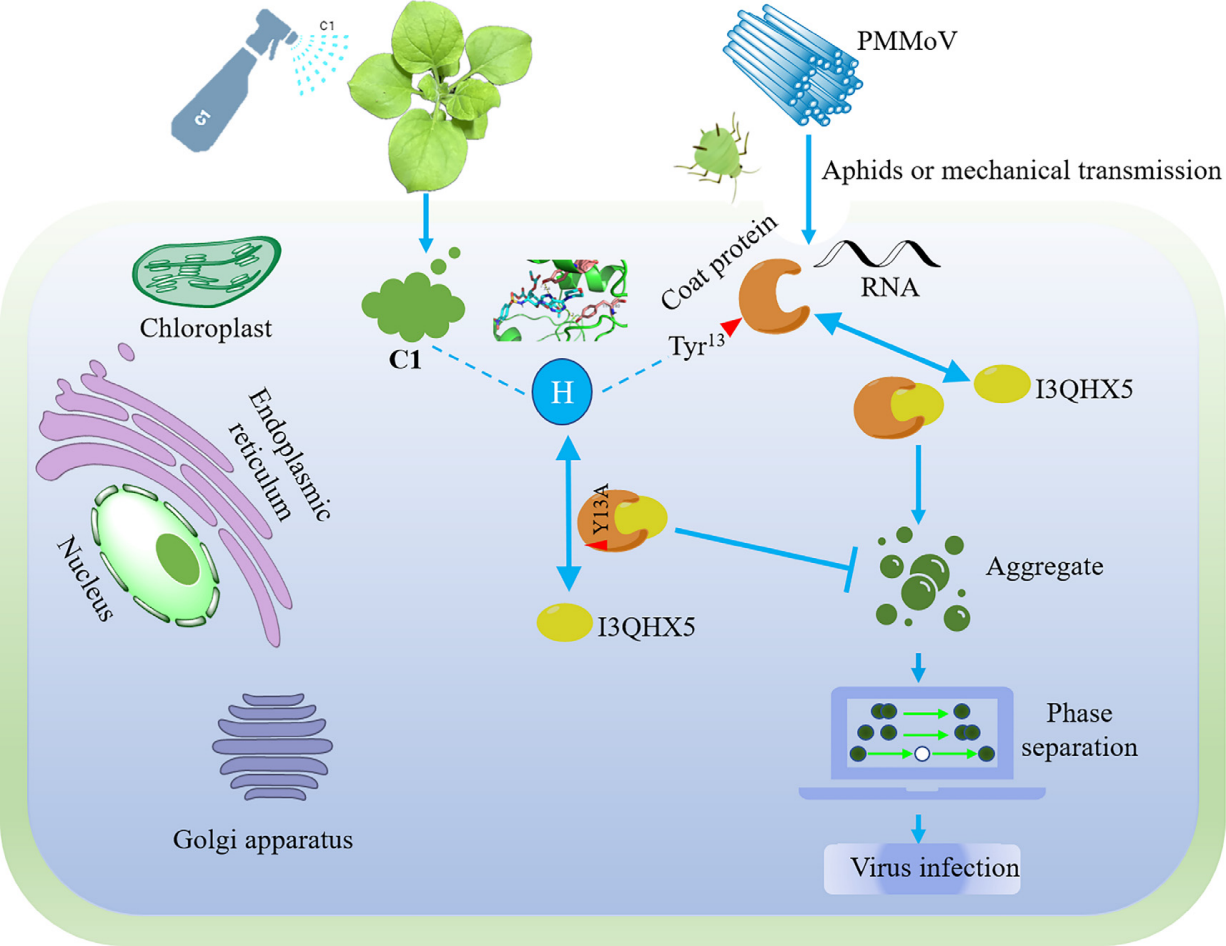
Proposed antiviral mechanism of **C1** through modulating phase separation in PMMoV infection. Overexpression of Flag-PMMoV CP triggered rapid upregulation of I3QHX5, and silencing of I3QHX5 significantly attenuated systemic invasion of PMMoV. PMMoV CP promoted LLPS by interacting with I3QHX5 to facilitate viral replication, while PMMoV CP^Y13A^ could not. And **C1** specifically targeted PMMoV CP^Y13A^ to inhibit LLPS, thereby suppressing viral replication.

As displayed in Fig. 7, compared with the *N. benthamiana* inoculated with TRV:00, the expression of I3QHX5 with TRV:I3QHX5 decreased to approximately 60 % of normal levels, while the plant did not show any obvious phenotype at 10 dpi. And *N. benthamiana* with TRV:00 showed more severe crumpling symptoms and a broader range of green fluorescence than silenced plants of TRV:I3QHX5 inoculated with GFP-PMMoV at 8 dpi. Western blot and RT-qPCR demonstrated that silencing of I3QHX5 leading to an attenuated accumulation of PMMoV, revealing that PMMoV CP could regulate the protein of I3QHX5 to facilitate viral transmission (see Fig. 8).

As shown in Fig. 7F, the accumulation of PMMoV RNAs in protoplasts of TRV:00 and TRV:I3QHX5 were lower after transfection of pCB-GFP-PMMoV for 12 h, while the accumulation level of PMMoV RNAs in TRV:I3QHX5 was less than that in TRV:00. And the accumulation level of PMMoV RNAs in TRV:00 was significantly higher than that of TRV:I3QHX5 after transfection of pCB-GFP-PMMoV for 24 h. The above results further indicated that silencing of I3QHX5 impaired the level of viral replication.

## Discussion

In this study, a series of purine morpholine nucleoside analogues containing a sulfonamide fragment, including **C1**, **C10**, **C12**, **C19**, **C22**, **C31**, **C32**, and **C34** with preferable inhibitory activities towards PMMoV, were designed and synthesized by modifying and reforming both the purine and sugar rings. And the EC_50_ value for the inactivating activity of **C1** against PMMoV was superior to those of **C12**, **C31**, **C32**, and **C34**. Although the EC_50_ values for inactivating activities against PMMoV of **C10**, **C19**, and **C22** were better than that of **C1**, the inactivating activity of **C1** was superior to those of **C10**, **C19**, and **C22** at 500 µg/mL. Based on the dual evaluation system of EC_50_ value and inactivating efficiency, **C1** was chosen as a representative to investigate the inactivating mechanism of series **C** acting on PMMoV.

Firstly, the small molecule probe **C2** was synthesized by introducing an alkyne group into the purine ring through a modification on **C1**, and the inactivating activity of **C2** against PMMoV was comparable to that of **C1**, suggesting that **C2** could be used as a small molecule probe to capture target protein. It could be observed from the results of ABPP that, at the identical concentration, **C2** was capable of capturing the protein with a molecular weight of 17 kDa, whereas neither **C1** nor DMSO exhibited such a capacity. Of the four open reading frames of PMMoV, only PMMoV CP corresponded to a size of 17.5 kDa, indicating that the target protein of **C1** acting on PMMoV was PMMoV CP. Concentration-dependent, competitive inhibition assays and MST further confirmed that PMMoV CP was a target of **C1**.

Complementary molecular docking analyses confirmed that both **C1** and **C2** interacted with Tyr13 and Tyr140 residues of PMMoV CP (Fig. S134). Together, these computational and experimental data strongly suggested that **C1** and **C2** competed mechanistically for the overlapping binding sites on PMMoV CP and thus might interfere with viral processes through similar pathways. In order to investigate the effects of Tyr13 and Tyr140 on virus infection, infectious clones of GFP-PMMoV CP^Y13A^ and GFP-PMMoV CP^Y140A^ were constructed. RT-qPCR and Western blot revealed that the substitution of tyrosine with alanine at position 140 (Y140A) in the PMMoV CP delayed the systemic infection of PMMoV, while the mutation of Y13A inhibited the systemic infection. These findings highlighted that Tyr13 of PMMoV CP played an essential role than Tyr140 in viral infection, which was consistent with the result of molecular docking and MD simulations. Subsequently, Co-IP MS and RNA-seq were proposed to analyze the reasons for this diversity by looking for differences between PMMoV CP and PMMoV CP^Y13A^.

A total of 5 proteins designated A0A248QEL2, B1PSM0, I3QHX5, LOC109221810, and A2IBL2 were identified through Co-IP MS, RNA-seq and RT-qPCR. From the results of LCA and BiFC, it can be seen that both PMMoV CP and PMMoV CP^Y13A^ interacted with A0A248QEL2, B1PSM0, and I3QHX5, but not with LOC109221810 and A2IBL2. It was entertaining that a large number of aggregates were formed after the interaction between PMMoV CP and I3QHX5, while no aggregates were produced with the interaction between PMMoV CP^Y13A^ and I3QHX5 both *in vivo* and *in vitro* (Fig. S142). The formation of aggregates was closely related to phase separation as reported in literature [59,60]. In cells, certain proteins and other biomolecules could concentrate together through phase separation to form aggregates. These aggregates might serve as “reaction centers” within cells, promoting specific biochemical reactions or signaling processes. Phase separation has become a fundamental principle for organizing and dividing cellular processes and played an important role in the lifecycle of viruses. It regarded as the foundation for the formation of certain virus factories, such as inclusion bodies (IB), which could achieve efficient virus replication and particle assembly, and served as targets for antiviral agents [59,60]. Based on the fusion, fission and FRAP, it was demonstrated that CP and I3QHX5 could form LLPS both *in vivo* and *in vitro*, which further indicated that the formation of LLPS might be the main reason for the difference in the systemic infection between GFP-PMMoV CP and GFP-PMMoV CP^Y13A^. As shown in Fig. 6A, the addition of **C1** at 30, 36, and 42 hpi also affected the interactions of PMMoV CP with I3QHX5. Further suggesting that the antiviral activity exhibited by **C1** might be due to the inhibition of aggregate formation. Tyrosine has been proven to be crucial for phase separation. Mutations or deletions of tyrosine residues could reduce their phase separation tendency [[61], [62], [63]]. This result was consistent with that the mutation of amino acid at 13 from tyrosine to alanine in PMMoV CP inhibited the propensity to form phase separation with I3QHX5.

Since it was tentatively concluded that the diverse interactions in PMMoV CP and PMMoV CP^Y13A^ with I3QHX5 were responsible for their differences in viral infection, what role did I3QHX5 play in virus infestation? Subsequently, VIGS was utilized to investigate the role of I3QHX5 played in viral infection. As shown in Fig. 7C, TRV:00 controls exhibited pronounced leaf curling phenotypes and enhanced green fluorescent signals at 8 dpi inoculated with GFP-PMMoV, while TRV:I3QHX5 plants displayed diminished fluorescent intensity and absence of phenotypic abnormalities under identical experimental conditions. Western blot and RT-qPCR manifested that silencing of I3QHX5 in *N. benthamiana* significantly reduced the accumulation of PMMoV CP and CP mRNA, indicating a critical role of I3QHX5 in viral systemic spread. And the accumulation level of PMMoV RNAs in protoplasts of TRV:00 was significantly higher than that of TRV:I3QHX5 after transfection of pCB-GFP-PMMoV for 24 h, further suggesting that silencing of I3QHX5 impaired the efficiency of viral replication. This finding was consistent with previous reports in the literature that AHCY (or SAHH) played crucial roles in viral infection, replication, or immune evasion across diverse viruses by regulating methylation metabolism or directly participating in the viral life cycle, thereby offering potential molecular targets for antiviral strategies [[64], [65], [66]].

In summary, our research findings revealed that **C1** had significant potential as an antiviral agent. Further molecular docking, Co-IP MS, RNA-seq, LCA, BiFC, FRAP, and VIGS indicated that PMMoV CP could form LLPS through interaction with I3QHX5, while PMMoV CP^Y13A^ could not, thereby explaining the observed differences in viral replication efficiency.

## Conclusion

In this study, thirty-eight purine morpholine nucleoside analogues incorporating a sulfonamide fragment with favorable activities against PMMoV were designed and synthesized. ABPP technology, Western blot and MST demonstrated that PMMoV CP was the target protein of **C1**. Molecular docking, MD simulations, confocal, Western blot and RT-qPCR revealed that Y13A in PMMoV CP could severely inhibit virus infection. Correlated mechanism studies revealed that PMMoV CP could aggregate after interacting with I3QHX5, while PMMoV CP^Y13A^ could not under confocal. Fusion, fission, and FRAP demonstrated that the condensed material formed by CP and I3QHX5 had the properties of LLPS both *in vivo* and *in vitro*. These findings indicated that **C1** could inhibit the formation of LLPS by specifically targeting Tyr13 of PMMoV CP, thus achieving the purpose of antiviral effects. Therefore, this study not only pointed out the direction for the research and development of novel antiviral agents, but also gave a certain reference for the study of antiviral mechanisms.

## Compliance with ethics requirement

This article does not contain any studies with human or animal subjects.

## CRediT authorship contribution statement

**Yuyuan Yang:** Methodology, Investigation, Visualization, Data curation, Formal analysis, Writing – original draft. **Runjiang Song:** Conceptualization, Supervision, Funding acquisition, Project administration, Writing – review & editing. **Shaobo Wang:** Formal analysis, Writing – original draft. **Guangcheng Zu:** Formal analysis, Writing – original draft. **Baoan Song:** Conceptualization, Supervision, Funding acquisition, Project administration, Writing – review & editing.

## Funding

We acknowledge the financial support from the 10.13039/501100001809National Natural Science Foundation of China (No. 32330087 & 32302388), the Key Technologies R&D Program of Guizhou Province in China (No. 2017-5788-1), and the Scientific Research Innovation Team of Guizhou University (No. 202403).

## Declaration of competing interest

The authors declare that they have no known competing financial interests or personal relationships that could have appeared to influence the work reported in this paper*.*

## References

[1] Gómez-García M., Ochoa-Alejo N. (2013). Biochemistry and molecular biology of carotenoid biosynthesis in chili peppers (*Capsicum* spp.). Int J Mol Sci.

[2] Kumari N., Sharma V., Patel P., Sharma P.N. (2023). *Pepper mild mottle virus*: a formidable foe of capsicum production-a review. Front Virol.

[3] Dairam A., Fogel R., Daya S., Limson J.L. (2008). Antioxidant and iron-binding properties of curcumin, capsaicin, and *S*-allylcysteine reduce oxidative stress in rat brain homogenate. J Agric Food Chem.

[4] Hernández-Pérez T., Gómez-García M.D.R., Valverde M.E., Paredes-López O. (2020). *Capsicum annuum* (hot pepper): an ancient Latin‐American crop with outstanding bioactive compounds and nutraceutical potential. Compr Rev Food Sci Food Saf.

[5] Arogundade O., Balogun O.S., Kareem K.T. (2012). Occurrence and distribution of *pepper veinal mottle virus* and *cucumber mosaic virus* in pepper in Ibadan, Nigeria. Virol J.

[6] Waweru B.W., Miano D.W., Kilalo D.C., Rukundo P., Kimenju J.W. (2021). Detection and distribution of viruses infecting hot pepper (*Capsicum* spp.) in Rwanda. J Plant Pathol.

[7] Rialch N., Sharma V., Sharma A., Sharma P.N. (2015). Characterization and complete nucleotide sequencing of *pepper mild mottle virus* infecting bell pepper in India. Phytoparasitica.

[8] Kenyon L., Kumar S., Tsai W.S., Hughes J.d'A. (2014). Virus diseases of peppers (*Capsicum* spp.) and their control. Adv Virus Res.

[9] Colson P., Richet H., Desnues C., Balique F., Moal V., Jean-Jacques J. (2010). *Pepper mild mottle virus*, a plant virus associated with specific immune responses, fever, abdominal pains, and pruritus in humans. PLoS One.

[10] Aguado-García Y., Taboada B., Morán P., Rivera-Gutiérrez X., Serrano-Vázquez A., Iša P. (2020). *Tobamoviruses* can be frequently present in the oropharynx and gut of infants during their first year of life. Sci Rep.

[11] Chen J.X., Luo X., Chen Y.F., Wang Y., Peng J., Xing Z.F. (2022). Recent research progress: discovery of anti-plant virus agents based on natural scaffold. Front Chem.

[12] Schaeffer H.J., Beauchamp L., de Miranda P., Elion G.B., Bauer D.J., Collins P. (1978). 9-(2-hydroxyethoxymethyl) guanine activity against viruses of the herpes group. Nature.

[13] Laskin O.L., Stahl-Bayliss C.M., Kalman C.M., Rosecan L.R. (1987). Use of ganciclovir to treat serious *cytomegalovirus* infections in patients with AIDS. J Infect Dis.

[14] de Carvalho O.V., Félix D.M., de Camargo T.C., Fietto J.L.R., de Almeida M.R., Bressan G.C. (2017). 6-methylmercaptopurine riboside, a thiopurine nucleoside with antiviral activity against *canine distemper virus in vitro*. Virol J.

[15] Kohgo S., Imoto S., Tokuda R., Takamatsu Y., HigashiKuwata N., Aoki M. (2018). Synthesis of 4′‐substituted purine 2′‐deoxynucleosides and their activity against *human immunodeficiency virus* type 1 and *hepatitis B virus*. ChemistrySelect.

[16] Luo M., Groaz E., De Jonghe S., Schols D., Herdewijn P. (2019). Synthesis and anti‐HIV activity of guanine modified fluorinated acyclic nucleoside phosphonate derivatives. Chem Biodivers.

[17] De Clercq E. (1987). S-adenosylhomocysteine hydrolase inhibitors as broad-spectrum antiviral agents. Biochem Pharmacol.

[18] Noguchi T., Yasuda Y., Niida T., Shomura T. (1968). Inhibitory effects of miharamycin A on the multiplication of plant viruses and the symptom development. Ann Phytopath Soc Japan.

[19] Lozoya-Saldana H., Dawson W.O., Murashige T. (1984). Effects of ribavirin and adenine arabinoside on *tobacco mosaic virus* in *Nicotiana tabacum* L. var Xanthi tissue cultures. Plant Cell Tiss Organ Cult.

[20] Dawson W.O. (1984). Effects of animal antiviral chemicals on plant viruses. Phytopathology.

[21] De Fazio G., Vicente M., De Clercq E. (1987). Antiviral effects of dihydroxypropyladenine ((*RS*)-DHPA) and bromovinyldeoxyuridine (BVDU) on plant viruses. Antiviral Res.

[22] Chen Z., Xu W.M., Liu K.M., Yang S., Fan H.T., Bhadury P.S. (2010). Synthesis and antiviral activity of 5‑(4‑chlorophenyl)-1,3,4-thiadiazole sulfonamides. Molecules.

[23] Zhou D.G., Xie D.D., He F.C., Song B.A., Hu D.Y. (2018). Antiviral properties and interaction of novel chalcone derivatives containing a purine and benzenesulfonamide moiety. Bioorg Med Chem Lett.

[24] Yang Y.Y., Zhang J., Li X.Y., He F.C., Wu R., Hu D.Y. (2020). Discovery of dithioacetal derivatives containing sulfonamide moiety of novel antiviral agents by TMV coat protein as a potential target. ACS Omega.

[25] Jiang D.H., Chen J.X., Zan N.N., Li C.Y., Hu D.Y., Song B.A. (2021). Discovery of novel chromone derivatives containing a sulfonamide moiety as anti-ToCV agents through the *tomato chlorosis virus* coat protein-oriented screening method. J Agric Food Chem.

[26] Yang Y.Y., Hu D.Y., Wang S.B., Wang Z.J., Zu G.C., Song B.A. (2022). First discovery of novel cytosine derivatives containing a sulfonamide moiety as potential antiviral agents. J Agric Food Chem.

[27] Li C.Y., Song R.J., He S.Q., Wu S.K., Wu S., Wu Z.X. (2022). First discovery of imidazo[1,2-*α*]pyridine mesoionic compounds incorporating a sulfonamide moiety as antiviral agents. J Agric Food Chem.

[28] He F.C., Shi J., Wang Y.J., Wang S.B., Chen J.X., Gan X.H. (2019). Synthesis, antiviral activity, and mechanisms of purine nucleoside derivatives containing a sulfonamide moiety. J Agric Food Chem.

[29] Tang X., Lei L., Liao A.J., Sun W., Zhang J., Wu J. (2023). Morpholine derivatives in agrochemical discovery and development. J Agric Food Chem.

[30] Ajayi K., Thakur V.V., Lapo R.C., Knapp S. (2010). Intramolecular *α*-glucosaminidation: synthesis of mycothiol. Org Lett.

[31] Qi C.M., He Y., Wang X., Feng M., Xu J.L., Ding R. (2011). Synthesis and evaluation of *N*-(2-[^18^F]fluoro-4-nitrobenzoyl)glucosamine: a preliminary report. J Radioanal Nucl Chem.

[32] Wang J., Qi W.J., Chen G.S. (2019). The effect of monosaccharides on self-assembly of benzenetricarboxamides. Chin Chem Lett.

[33] Thanh N.D., Hai D.S., Ngoc Bich V.T., Thu Hien P.T., Ky Duyen N.T., Mai N.T. (2019). Efficient click chemistry towards novel 1*H*-1,2,3-triazole-tethered 4*H*-chromene-D-glucose conjugates: Design, synthesis and evaluation of *in vitro* antibacterial, MRSA and antifungal activities. Eur J Med Chem.

[34] Yang Y.Y., Song R.J., Yin L.M., Han K.L., Yan F., Song B.A. (2023). Inactivating activities and mechanism of imidazo[1,2-*c*]pyrimidin-5(6*H*)-one nucleoside derivatives incorporating a sulfonamide scaffold. J Agric Food Chem.

[35] Novosjolova I., Bizdēna Ē., Turks M. (2013). Application of 2,6-diazidopurine derivatives in the synthesis of thiopurine nucleosides. Tetrahedron Lett.

[36] Lougiakis N., Gavriil E.S., Kairis M., Sioupouli G., Lambrinidis G., Benaki D. (2016). Design and synthesis of purine analogues as highly specific ligands for FcyB, a ubiquitous fungal nucleobase transporter. Bioorg Med Chem.

[37] Wang Y.N., Bheemanaboina R.R.Y., Cai G.X., Zhou C.H. (2018). Novel purine benzimidazoles as antimicrobial agents by regulating ROS generation and targeting clinically resistant *Staphylococcus aureus* DNA groove. Bioorg Med Chem Lett.

[38] Patching S.G., Baldwin S.A., Baldwin A.D., Young J.D., Gallagher M.P., Henderson P.J.F. (2005). The nucleoside transport proteins, NupC and NupG, from *Escherichia coli*: specific structural motifs necessary for the binding of ligands. Org Biomol Chem.

[39] Wang S.J., Zhu W.J., Wang X., Li J.G., Zhang K.H., Zhang L.R. (2014). Design, synthesis and SAR studies of NAD analogues as potent inhibitors towards CD38 NADase. Molecules.

[40] Gooding G.V., Hebert T.T. (1967). A simple technique for purification of *tobacco mosaic virus* in large quantities. Phytopathology.

[41] Wei C.L., Zhao C.N., Li J., Li C.Y., Song B.A., Song R.J. (2024). Innovative arylimidazole-fused phytovirucides via carbene-catalyzed [3+4] cycloaddition: locking viral cell-to-cell movement by out-competing virus capsid-host interactions. Adv Sci.

[42] Wang Z.Z., Xie D.D., Gan X.H., Zeng S., Zhang A.W., Yin L.M. (2017). Synthesis, antiviral activity, and molecular docking study of trans-ferulic acid derivatives containing acylhydrazone moiety. Bioorg Med Chem Lett.

[43] Bhyravbhatla B., Watowich S.J., Caspar D.L.D. (1998). Refined atomic model of the four-layer aggregate of the *tobacco mosaic virus* coat protein at 2.4-Å resolution. Biophys J.

[44] Rose P.W., Prlić A., Altunkaya A., Bi C., Bradley A.R., Christie C.H. (2017). The RCSB protein data bank: integrative view of protein, gene and 3D structural information. Nucleic Acids Res.

[45] Wang H.C., Wu J.S., Chia J.C., Yang C.C., Wu Y.J., Juang R.H. (2009). Phytochelatin synthase is regulated by protein phosphorylation at a threonine residue near its catalytic site. J Agric Food Chem.

[46] Lovell S.C., Davis I.W., Arendall W.B., de Bakker P.I., Word J.M., Prisant M.G. (2003). Structure validation by Calpha geometry: phi, psi and Cbeta deviation. Proteins.

[47] Read R.J., Adams P.D., Arendall W.B., Brunger A.T., Emsley P., Joosten R.P. (2011). A new generation of crystallographic validation tools for the protein data bank. Structure.

[48] Wu F.X., Wang F., Yang J.F., Jiang W., Wang M.Y., Jia C.Y. (2020). AIMMS suite: a web server dedicated for prediction of drug resistance on protein mutation. Brief Bioinform.

[49] Cosconati S., Forli S., Perryman A.L., Harris R., Goodsell D.S., Olson A.J. (2010). Virtual screening with AutoDock: theory and practice. Expert Opin Drug Discov.

[50] Jakalian A., Jack D.B., Fast B.CI. (2002). efficient generation of high-quality atomic charges. AM1-BCC model: II. Parameterization and validation. J Comput Chem.

[51] Jakalian A., Bush B.L., Jack D.B., Christopher (2000). Fast, efficient generation of high-quality atomic charges. AM1-BCC model. I. Method. J Comput Chem.

[52] Maier J.A., Martinez C., Kasavajhala K., Wickstrom L., Hauser K.E., Simmerling C. (2015). ff14SB: Improving the accuracy of protein side chain and backbone parameters from ff99SB. J Chem Theory Comput.

[53] Wang B., Merz K.M. (2006). A fast QM/MM (quantum mechanical/molecular mechanical) approach to calculate nuclear magnetic resonance chemical shifts for macromolecules. J Chem Theory Comput.

[54] Wang J., Wolf R.M., Caldwell J.W., Kollman P.A., Case D.A. (2004). Development and testing of a general amber force field. J Comput Chem.

[55] Price D.J., Brooks C.L. (2004). A modified TIP3P water potential for simulation with Ewald summation. J Chem Phys.

[56] Jorgensen W.L., Chandrasekhar J., Madura J.D., Impey R.W., Klein M.L. (1983). Comparison of simple potential functions for simulating liquid water. J Chem Phys.

[57] Chen B.H., Lin L., Lu Y.W., Peng J.J., Zheng H.Y., Yang Q.K. (2020). Ubiquitin-Like protein 5 interacts with the silencing suppressor p3 of *rice stripe virus* and mediates its degradation through the 26S proteasome pathway. PLOS Pathog.

[58] Jiang S.S., Lu Y.W., Li K.F., Lin L., Zheng H.Y., Yan F. (2014). Heat shock protein 70 is necessary for rice stripe virus infection in plants. Mol Plant Pathol.

[59] Etibor T., Yamauchi Y., Amorim M. (2021). Liquid biomolecular condensates and viral lifecycles: review and perspectives. Viruses.

[60] Li H., Ernst C., Kolonko-Adamska M., Greb-Markiewicz B., Man J., Parissi V. (2022). Phase separation in viral infections. Trends Microbiol.

[61] De Sancho D. (2022). Phase separation in amino acid mixtures is governed by composition. Biophys J.

[62] Lin Y., Currie S.L., Rosen M.K. (2017). Intrinsically disordered sequences enable modulation of protein phase separation through distributed tyrosine motifs. J Biol Chem.

[63] Pak C.W., Kosno M., Holehouse A.S., Padrick S.B., Mittal A., Ali R. (2016). Sequence determinants of intracellular phase separation by complex coacervation of a disordered protein. Mol Cell.

[64] Masuta C., Tanaka H., Uehara K., Kuwata S., Koiwai A., Noma M. (1995). Broad resistance to plant viruses in transgenic plants conferred by antisense inhibition of a host gene essential in S-adenosylmethionine-dependent transmethylation reactions. Proc Natl Acad Sci.

[65] Lim Y.S., Mai H.N., Nguyen L.P., Kang S.M., Tark D., Hwang S.B. (2021). Adenosylhomocysteinase like 1 interacts with nonstructural 5A and regulates *hepatitis C virus* propagation. J Microbiol.

[66] Denolly S., Stukalov A., Barayeu U., Rosinski A.N., Kritsiligkou P., Joecks S. (2023). *Zika virus* remodelled ER membranes contain proviral factors involved in redox and methylation pathways. Nat Commun.

