# Supplementary material for: Antiviral activity and mechanism of purine morpholine nucleoside analogues incorporating a sulfonamide fragment
Source: J Adv Res. 2025 Jun 1;81:43–56. doi: 10.1016/j.jare.2025.05.062 (PMC12957839; doi:10.1016/j.jare.2025.05.062)
Supplement: Supplementary Data 1 [file mmc1.docx]

**Supporting Information**

**Antiviral activity and mechanism of** **purine morpholine nucleoside analogues incorporating a** **sulfonamide fragment**

Yuyuan Yang^1^, Runjiang Song^1^, Shaobo Wang^1^, Guangcheng, Zu^1^, Baoan Song^1,^ *

^1^Current address: State Key Laboratory of Green Pesticide, Center for R&D of Fine Chemicals of Guizhou University, Guiyang 550025, P. R. China.

*Address of the corresponding author

Fax: 0086-851-83622211; E-mail: [basong@gzu.edu.cn](mailto:basong@gzu.edu.cn).

**Contents**

[1. Synthetic part	3](#_Toc198040432)

[1.1 General information	3](#_Toc198040433)

[1.1.1 Chemicals and instruments.	3](#_Toc198040434)

[1.2 Chemistry	3](#_Toc198040435)

[1.2.1 General procedures for the preparation of intermediates **a**-**e**.	4](#_Toc198040436)

[1.2.2 General procedure for the preparation of intermediate **h**.	5](#_Toc198040437)

[1.2.3 General procedure for the preparation of target compounds **C1**, **C3**-**C38**.	5](#_Toc198040438)

[1.2.4 General procedure for the preparation of target compounds **C2**.	5](#_Toc198040439)

[1.3 Characterization of target compounds	7](#_Toc198040440)

[1.4 ^1^H NMR, ^13^C NMR, ^19^F NMR and HRMS of target compounds **C1**-**C38**.	22](#_Toc198040441)

[2. Antiviral activities against PMMoV	86](#_Toc198040442)

[3. Mechanism of action	86](#_Toc198040443)

[3.1 Searching for the target protein of **C1** acted on PMMoV	86](#_Toc198040444)

[3.2 Determination of the dissociation constants of **C1** with PMMoV CP	87](#_Toc198040445)

[3.3 Molecular *modeling*	87](#_Toc198040446)

[3.3.1 Homology modeling	87](#_Toc198040447)

[3.3.2 Molecular docking	87](#_Toc198040448)

[3.3.3 Molecular Dynamics (MD) Simulations	88](#_Toc198040449)

[3.4 Construction of infectious clones	88](#_Toc198040450)

[3.5 Transient expression of agrobacterium tumefaciens	89](#_Toc198040451)

[3.6 Co-immunoprecipitation mass spectrometry (Co-IP MS)	89](#_Toc198040452)

[3.6.1 Vector construction and material preparation	89](#_Toc198040453)

[3.6.2 Beads binding antibody	89](#_Toc198040454)

[3.6.3 Antibody binding bait protein	90](#_Toc198040455)

[3.7 RNA sequencing (RNA-seq)	90](#_Toc198040456)

[3.8 Split-luciferase complementation assay (LCA)	90](#_Toc198040457)

[3.9 Bimolecular fluorescence complementation (BiFC)	91](#_Toc198040458)

[3.10 Virus-induced gene silencing (VIGS)	91](#_Toc198040459)

[3.11 Cell viability assay	92](#_Toc198040460)

# 1. Synthetic part

## 1.1 General information

1.1.1 Chemicals and instruments. The solvents and reagents used throughout the experiments were procured from commercial suppliers and used without further processing. ^1^H NMR, ^13^C NMR, and ^19^F NMR were obtained with a Bruker Ascend-400 spectrometer (Bruker, Germany) or a JEOL ECX-500 spectrometer (JEOL, Tokyo, Japan) in CDCl_3_ or MeOD. High-resolution mass spectra (HRMS) were acquired on an LTQ Orbitrap XL (Thermo Fisher Scientific, Waltham, MA). Melting points of the compounds were identified via a WRX-4 melting point apparatus (YiCe Apparatus & Equipment Co., Ltd., Shanghai, China). Single-crystal X-ray diffraction analysis of compound **C9** was carried out with a Bruker D8 VENTURE (Bruker, Germany).

## 1.2 Chemistry

**Scheme 1** Design of the title compounds

1.2.1 General procedures for the preparation of intermediates a-e. The intermediates **a-e** were synthesized according to the methods reported in literatures (Org Lett 2010;12:2630-2633, J Radioanal Nucl Chem 2011;287:913-920, Eur J Med Chem 2019;167:454-471, Chin Chem Lett 2019;30:587-591, J Agric Food Chem 2023;71:7977-7987). Firstly, Ɒ-glucosamine hydrochloride (10.0 g, 46.4 mmol) was dissolved in a stirred aqueous sodium hydroxide solution (47.0 mL, 1M, 47.0 mmol) at 0 °C, and benzaldehyde (5.7 g, 5.4 mL, 53.3 mmol) was added dropwise to the mixture after the solution became clear. Upon formation of a large amount of white solid, stirring was continued for 30 min, and the mixture was maintained at 0 °C for an additional 12 h. The crude product was filtered and washed with water and cold EtOH to afford intermediate **a**. Next, Ac_2_O (39.9 g, 40.0 mL, 391.0 mmol) was slowly added to a stirring mixture of **a** (11.0 g, 41.2 mmol) in anhydrous pyridine (63.5 g, 64.8 mL, 802.5 mmol) at 0 °C. The reaction was then heated to 40 °C for 6 h until completion. Approximately half of the solvent was removed under reduced pressure at 30 °C, and the residue was poured into ice water. After stirring for 1 h, the mixture was filtered to yield product **b**. Then, a transparent solution of **b** (5.0 g, 11.5 mmol) in EtOH at 50-60 °C was treated with 200 *µ*L of conc. HCl. Upon precipitation of white solids, heating was stopped, and the mixture was stirred in an ice bath for an additional hour. Intermediate **c** was isolated by filtration. Furthermore, various substituted sulfonyl chloride (4.4 mmol) dissolved in DCM (20 mL) was reacted with **c** (1.5 g, 3.9 mmol) using Et_3_N (9.8 mmol) as a base at rt for 12-24 h. The mixture was then diluted with DCM (20 mL) and washed with water (3 × 50 mL). The organic layer was dried over anhydrous MgSO₄, filtered, and concentrated under reduced pressure. The crude product was recrystallized from a DCM/hexane mixture to afford intermediates **d1**-**d18**. Finally, 33% HBr (8.0 mL) was added to a solution of **d** (997.0 *µ*mol) in anhydrous DCM (20 mL). The reaction was sealed and maintained at 0 °C for 6 h. After dilution with DCM (20 mL), the mixture was washed with water (3 × 50 mL). The organic layer was dried over anhydrous MgSO_4_, filtered, and concentrated under reduced pressure. The residue was purified by column chromatography (hexane/ethyl acetate, 3:1 v/v) to yield products **e1-e18**.

1.2.2 General procedure for the preparation of intermediate h. To the mixture of purine derivative **g** (323.5 *µ*mol) and sodium hydride (8.2 mg, 339.7 *µ*mol) in anhydrous acetonitrile (10 mL), 2-(acetoxymethyl)-6-bromo-5-((substituted phenyl)sulfonamidyl) tetrahydro-2*H*-pyran-3,4-diacetyldiacetate **e** (148.0 mg, 291.2 *µ* mol) in acetonitrile (5 mL) was slowly added at rt. After the reaction was complete, the mixture was quenched with ethanol. The solvent was then concentrated under reduced pressure. The residue was dissolved in DCM (20 mL) and washed with water (3 × 50 mL). Finally, intermediate **h** was separated and purified by column chromatography (DCM/EA, 1:1 v/v) (Tetrahedron Lett 2013;54:6557-6561).

1.2.3 General procedure for the preparation of target compounds C1, C3-C38. To the mixture of morpholine (20.0 mg, 229.6 *µ*mol) and triethylamine (69.7 mg, 688.7 *µ*mol) in ethanol (10 mL), 2-(acetoxymethyl)-5-((substituted phenyl)sulfonamidyl)-6-(2-substituted-6-chloro-9*H*-purin-9-yl)tetrahydro-2*H*-pyran-3,4-dicarboxylic acid dihydrate **h** (183.7 *µ*mol) dissolved in ethanol (5 mL) was slowly added, and the mixture was heated to reflux. The reaction was monitored by TLC until full completion, and the solvent was removed by rotary evaporation. Then the residue was dissolved in DCM (20 mL) and washed with water (3 × 20 mL). The organic layer was dried over anhydrous MgSO_4_, filtered, and concentrated under reduced pressure. Finally, the crude product **C1**, **C3**-**C35** were purified by column chromatography (DCM/EA, 1:1 v/v) (Bioorg Med Chem Lett 2016;24:5941-5952, Bioorg Med Chem Lett 2018;28:1621-1628).

Randomly selected compounds **C1**, **C25**, and **C34** (146.8 mol) in anhydrous methanol (10 mL), and added methanol/sodium methoxide solution dropwise until the reaction mixture was basic (pH = 10). The mixture was agitated at rt until the reaction was complete. It was then concentrated under reduced pressure, and the residue was purified by column chromatography (DCM/MeOH, 10:1 v/v) to acquire compounds **C36**-**C38** (Org Biomol Chem 2005;3:462-470, Molecules 2014;19:15754-15767, J Agric Food Chem 2023;71:7977-7987).

1.2.4 General procedure for the preparation of target compounds C2. To the solution of piperazine (82.4 mg, 95.7 *µ*mol) and triethylamine (32.3 mg, 323.5 *µ*mol) in ethanol (10 mL), 2-(acetoxymethyl)-5-((4-nitrophenyl)sulfonylamino)-6-(6-chloro-9*H*-purin-9-yl)tetrahydro-2*H*-pyran-3,4-dicarboxylic acid dihydrate (200 mg, 319.0 *µ*mol) in ethanol (5 mL) was slowly added and then heated the mixture to reflux. The reaction was monitored by TLC (DCM/MeOH, 10:1 v/v) until completion. The residue was then dissolved in DCM, washed with water (3 × 50 mL), dried over anhydrous MgSO₄, and concentrated under reduced pressure. Finally, the residue was purified by column chromatography (DCM/EA, 1:1 v/v) to afford intermediate **i** (Bioorg Med Chem 2016;24(22):5941-5952).

To the mixture of propargyl bromide (35.2 mg, 295.6 *µ*mol) and potassium carbonate (30.6 mg, 221.7 *µ*mol) in acetone (10 mL), 2-(acetoxymethyl)-5-((4-nitrophenyl)sulfonylamino)-6-(6-(piperazine-1-yl)-9*H*-purine-9-yl)tetrahydro-2*H*-pyrano-3,4-diacetyldiacetate (100 mg, 147.8 *µ*mol) dissolved in acetone (5 mL) was dropwise added at rt. The mixture was concentrated under reduced pressure after the reaction was finished. And the residue was dissolved in DCM (20 mL) and washed with water (3 × 20 mL). The organic layer was dried over anhydrous MgSO_4_, filtered, and concentrated under reduced pressure. Finally, the residue was purified by column chromatography (DCM/EA, 1:1 v/v) to yield the target compound **C2**.

**Scheme 2** Synthesis of target compounds **C1**-**C38**

## 1.3 Characterization of target compounds

*2-(acetoxymethyl)-6-(6-morpholino-9H-purin-9-yl)-5-((4-nitrophenyl)sulfonamido) tetrahydro-2H-pyran-3,4-diyl diacetate* (**C1**). White solid; m. p. 140.1-141.0 °C; yield 65.2%; ^1^H NMR (400 MHz, CDCl_3_) *δ*: 8.27 (s, 1H, purine-8-H), 7.80 (s, 1H, purine-2-H), 7.21 (d, *J* = 8.7 Hz, 2H, Ar-2,6-H), 7.04 (d, *J* = 8.7 Hz, 2H, Ar-3,5-H), 6.51 (d, *J* = 7.0 Hz, 1H, -SO_2_NH-), 5.79 (d, *J* = 9.8 Hz, 1H, 6’-H), 5.41 - 5.29 (m, 2H, 4’, 3’-H), 4.34 - 4.13 (m, 5H, 2’-H, morpholine-2,6-H), 4.12 - 4.08 (m, 1H, -CH_2_-), 3.98 (ddd, *J* = 9.8, 4.9, 2.1 Hz, 1H, -CH_2_-), 3.86 (s, 4H morpholine-3,5-H), 2.65 (s, 1H, 5’-H), 2.15 (s, 3H, -COCH_3_), 2.09 (s, 3H, -COCH_3_), 2.04 (s, 3H, -COCH_3_); ^13^C NMR (100 MHz, CDCl_3_, ppm) *δ*: 171.61, 170.60, 169.41, 153.34, 152.32, 150.00, 148.95, 145.83, 135.20, 126.46, 123.39, 118.88, 80.93, 75.10, 73.09, 67.65, 66.72, 61.72, 60.49, 59.18, 20.87, 20.73, 20.67; HRMS (ESI): Calculated for C_27_H_31_N_7_O_12_S [M-H]^-^: 676.16677, found: 676.16754.

*2-(acetoxymethyl)-5-((4-nitrophenyl)sulfonamido)-6-(6-(4-(prop-2-yn-1-yl) piperazin-1-yl)-9H-purin-9-yl)tetrahydro-2H-pyran-3,4-diyl diacetate* (**C2**). White solid; m. p. 113.4-114.3 °C; yield 68.7%; ^1^H NMR (400 MHz, CDCl_3_) *δ*: 8.13 (s, 1H, purine-8-H), 7.82 (d, *J* = 8.9 Hz, 2H, Ar-2,6-H), 7.69 (s, 1H, purine-2-H), 7.35 (d, *J* = 8.9 Hz, 2H, Ar-3,5-H), 6.84 (d, *J* = 78.8 Hz, 1H, -SO_2_NH-), 5.69 (d, *J* = 9.8 Hz, 1H, 6’-H), 5.30 (dt, *J* = 32.9, 9.6 Hz, 2H, -CH_2_-), 4.63 (s, 1H, 4’-H), 4.21 - 4.02 (m, 5H, 3’-H, morpholine-2,6-H), 3.91 (ddd, *J* = 9.8, 5.0, 2.0 Hz, 1H, 2’-H), 3.61 (dd, *J* = 13.4, 6.7 Hz, 1H, 5’-H), 3.31 (d, *J* = 2.2 Hz, 2H, Alkynyl-CH_2_-), 2.58 (s, 5H, Alkynyl-CH-, morpholine-3,5-H), 2.15 (s, 3H, -COCH_3_), 2.03 (s, 3H, -COCH_3_), 1.98 (s, 3H, -COCH_3_); ^13^C NMR (100 MHz, CDCl_3_, ppm) *δ*: 171.6, 170.60, 169.41, 153.15, 152.34, 149.92, 149.00, 145.75, 134.95, 126.45, 123.38, 118.80, 80.89, 78.28, 75.10, 73.70, 73.09, 67.64, 61.72, 59.21, 51.60, 46.87, 20.86, 20.73, 20.68; HRMS (ESI): Calculated for C_30_H_34_N_8_O_11_S [M+H]^+^: 715.21405, found: 715.21289.

*2-(acetoxymethyl)-6-(2-fluoro-6-morpholino-9H-purin-9-yl)-5-((4-nitrophenyl) sulfonamido)tetrahydro-2H-pyran-3,4-diyl diacetate* (**C3**). White solid; m. p. 151.4-152.2 °C; yield 65.3%; ^1^H NMR (500 MHz, CDCl_3_) *δ*: 7.98 (d, *J* = 8.8 Hz, 2H, Ar-2,6-H), 7.72 (s, 1H, purine-8-H), 7.50 (d, *J* = 8.9 Hz, 2H, Ar-3,5-H), 6.30 (d, *J* = 9.1 Hz, 1H, -SO_2_NH-), 5.65 (d, *J* = 9.8 Hz, 1H, 6’-H), 5.34 (dd, *J* = 11.4, 9.7 Hz, 2H, -CH_2_-), 4.67 (s, 1H, 4’-H), 4.23 (dd, *J* = 12.6, 5.1 Hz, 2H, 3’, 2’-H), 4.10 (dt, *J* = 23.9, 16.5 Hz, 4H, morpholine-2,6-H), 3.96 - 3.92 (m, 1H, 5’-H), 3.86 (s, 2H, morpholine-3,5-H), 3.70 (s, 2H, morpholine-3,5-H), 2.19 (s, 3H, -COCH_3_), 2.11 (s, 3H, -COCH_3_), 2.05 (s, 3H, -COCH_3_); ^13^C NMR (125 MHz, CDCl_3_) *δ*: 171.81, 170.67, 169.53, 157.82, 154.36, 151.68, 149.09, 146.14, 135.54, 126.70, 123.55, 120.40, 117.09, 80.91, 75.06, 72.98, 67.67, 66.70, 61.77, 59.38, 20.90, 20.79, 20.76; ^19^F NMR (471 MHz, CDCl_3_) *δ*: -49.65; HRMS (ESI): Calculated for C_27_H_29_FN_7_O_12_S [M-H]^-^: 694.15734, found: 694.15918.

*2-(acetoxymethyl)-6-(2-chloro-6-morpholino-9H-purin-9-yl)-5-((4-nitrophenyl) sulfonamido)tetrahydro-2H-pyran-3,4-diyl diacetate* (**C4**). White solid; m. p. 173.3-174.1 °C; yield 68.2%; ^1^H NMR (400 MHz, CDCl_3_) *δ*: 7.98 (d, *J* = 8.9 Hz, 2H, Ar-2,6-H), 7.73 (s, 1H, purine-8-H), 7.50 (d, *J* = 8.9 Hz, 2H, Ar-3,5-H), 6.23 (d, *J* = 9.4 Hz, 1H, -SO_2_NH-), 5.64 (d, *J* = 9.8 Hz, 1H, 6’-H), 5.38 - 5.30 (m, 2H, -CH_2_-), 4.61 (s, 1H, 4’-H), 4.31 - 4.05 (m, 6H, 3’, 2’-H, morpholine-2,6-H), 3.95 - 3.91 (m, 1H, 5’-H), 3.70 (s, 4H, morpholine-3,5-H), 2.19 (s, 3H, -COCH_3_), 2.11 (s, 3H, -COCH_3_), 2.07 (s, 3H, -COCH_3_); ^13^C NMR (100 MHz, CDCl_3_) *δ*: 171.53, 170.54, 169.35, 154.00, 153.26, 151.21, 149.03, 145.91, 135.27, 126.60, 123.49, 117.54, 81.01, 75.37, 72.77, 67.46, 66.68, 61.64, 59.36, 20.82, 20.74, 20.66; HRMS (ESI): Calculated for C_27_H_29_ClN_7_O_12_S [M-H]^-^: 710.12779, found: 710.12958.

*2-(acetoxymethyl)-6-(6-morpholino-9H-purin-9-yl)-5-((3-nitrophenyl)sulfonamido) tetrahydro-2H-pyran-3,4-diyl diacetate* (**C5**). White solid; m. p. 130.5-131.4 °C; yield 64.7%; ^1^H NMR (400 MHz, CDCl_3_) *δ*: 8.19 (s, 1H, Ar-4-H), 8.05 - 8.00 (m, 2H, Ar-2-H, purine-8-H), 7.69 (s, 1H, Ar-6-H), 7.62 - 7.56 (m, 1H, purine-2-H), 7.27 - 7.23 (m, 1H, Ar-5-H), 6.93 (d, *J* = 9.3 Hz, 1H, -SO_2_NH-), 5.80 (d, *J* = 9.8 Hz, 1H, 6’-H), 5.42 (t, *J* = 9.7 Hz, 1H, -CH_2_-), 5.29 (t, *J* = 9.7 Hz, 1H, -CH_2_-), 4.26 - 3.95 (m, 7H, 4, 3, 2’-H, and morpholine-2,6-H), 3.79 (s, 4H, morpholine-3,5-H), 2.35 (d, *J* = 2.6 Hz, 1H, 5’-H), 2.19 (s, 3H, -COCH_3_), 2.03 (s, 3H, -COCH_3_), 1.95 (s, 3H, -COCH_3_); ^13^C NMR (100 MHz, CDCl_3_) *δ*: 171.96, 170.61, 169.49, 153.34, 152.68, 149.99, 147.04, 142.49, 135.29, 130.87, 129.46, 126.13, 120.62, 118.70, 80.73, 74.81, 73.23, 67.54, 66.95, 61.67, 59.20, 20.91, 20.72; HRMS (ESI): Calculated for C_27_H_30_N_7_O_12_S [M-H]^-^: 676.16677, found: 676.16870.

*2-(acetoxymethyl)-6-(6-morpholino-9H-purin-9-yl)-5-((2-nitrophenyl)sulfonamido) tetrahydro-2H-pyran-3,4-diyl diacetate* (**C6**). White solid; m. p. 148.4-149.2 °C; yield 65.4%; ^1^H NMR (400 MHz, CDCl_3_) *δ*: 8.20 (s, 1H, Ar-3-H), 7.90 (s, 1H, purine-8-H), 7.64 (ddd, *J* = 14.0, 7.8, 1.4 Hz, 2H, purine-2-H, Ar-6-H), 7.52 - 7.43 (m, 2H, Ar-4,5-H), 7.07 (s, 1H, -SO_2_NH-), 5.92 (d, *J* = 9.7 Hz, 1H, 6’-H), 5.56 (t, *J* = 9.8 Hz, 1H, -CH_2_-), 5.33 (t, *J* = 9.8 Hz, 1H, -CH_2_-), 4.51 (s, 1H, 4’-H), 4.32 - 4.15 (m, 5H, morpholine-2,6-H, 3’-H), 4.06 (ddd, *J* = 10.1, 5.0, 2.1 Hz, 1H, 2’-H), 3.84 (t, *J* = 4.7 Hz, 4H, morpholine-3,5-H), 2.94 (s, 1H, 5’-H), 2.10 (s, 3H, -COCH_3_), 2.07 (s, 3H, -COCH_3_), 1.98 (s, 3H, -COCH_3_); ^13^C NMR (100 MHz, CDCl_3_) *δ*: 170.94, 170.72, 169.71, 153.44, 152.30, 150.38, 146.23, 136.40, 134.66, 132.76, 132.84, 129.19, 124.97, 119.14, 81.44, 74.93, 72.92, 68.15, 67.02, 61.93, 58.48, 20.74, 20.63, 20.49; HRMS (ESI): Calculated for C_27_H_30_N_7_O_12_S [M-H]^-^: 676.16677, found: 676.16858.

*2-(acetoxymethyl)-5-((4-cyanophenyl)sulfonamido)-6-(6-morpholino-9H-purin-9-yl)tetrahydro-2H-pyran-3,4-diyl diacetate* (**C7**). White solid; m. p. 145.1-146.0 °C; yield 62.8%; ^1^H NMR (400 MHz, CDCl_3_) *δ*: 8.22 (s, 1H, purine-8-H), 7.76 (s, 1H, purine-2-H), 7.55 (ddd, *J* = 16.3, 5.7, 3.0 Hz, 1H, Ar-2-H), 7.37 (d, *J* = 5.5 Hz, 3H, Ar-3,5,6-H), 6.61 (s, 1H, -SO_2_NH-), 5.79 (d, *J* = 9.8 Hz, 1H, 6’-H), 5.42 - 5.26 (m, 2H, -CH_2_-), 4.38 - 4.08 (m, 6H, 3’,4’-H, morpholine-2,6-H), 4.03 - 3.74 (m, 5H, 2’-H, morpholine-3,5-H), 2.20 (s, 1H, 5’-H), 2.15 - 2.00 (m, 9H, -COCH_3_); ^13^C NMR (100 MHz, CDCl_3_) *δ*: 171.35, 170.56, 169.37, 153.41, 151.93, 144.32, 142.01, 135.18, 131.96, 129.36, 125.99, 117.36, 115.56, 81.18, 75.28, 72.99, 67.58, 66.92, 61.67, 59.22, 20.74, 20.65; HRMS (ESI): Calculated for C_28_H_30_N_7_O_10_S [M-H]^-^: 656.17694, found: 656.17853.

*2-(acetoxymethyl)-6-(2-chloro-6-morpholino-9H-purin-9-yl)-5-((4-cyanophenyl) sulfonamido)tetrahydro-2H-pyran-3,4-diyl diacetate* (**C8**). White solid; m. p. 179.2-180.0 °C; yield 65.3%; ^1^H NMR (400 MHz, CDCl_3_) *δ*: 7.73 (s, 1H, purine-8-H), 7.44 (d, *J* = 2.2 Hz, 4H, Ar-2,6,3,5-H), 6.09 (s, 1H, -SO_2_NH-), 5.65 (d, *J* = 9.8 Hz, 1H, 6’-H), 5.37 - 5.27 (m, 2H, -CH_2_-), 4.76 (s, 1H, 4’-H), 4.33 - 4.10 (m, 5H, 3’-H, morpholine-2,6-H), 4.02 - 3.74 (m, 6H, 2,5’-H, morpholine-3,5-H), 2.17 (s, 3H, -COCH_3_), 2.12 (s, 3H, -COCH_3_), 2.08 (s, 3H, -COCH_3_); ^13^C NMR (100 MHz, CDCl_3_) *δ*: 171.64, 170.58, 169.38, 154.03, 153.37, 151.28, 144.33, 135.28, 131.98, 126.06, 117.60, 117.33, 115.65, 80.96, 75.29, 72.76, 67.47, 66.87, 61.65, 59.37, 20.84, 20.77, 20.70; HRMS (ESI): Calculated for C_28_H_29_ClN_7_O_10_S [M-H]^-^: 690.13796, found: 690.14026.

*2-(acetoxymethyl)-5-((4-chlorophenyl)sulfonamido)-6-(6-morpholino-9H-purin-9-yl)tetrahydro-2H-pyran-3,4-diyl diacetate* (**C9**). White solid; m. p. 127.7-128.5 °C; yield 68.1%; ^1^H NMR (400 MHz, CDCl_3_) *δ*: 8.27 (s, 1H, purine-8-H), 7.80 (s, 1H, purine-2-H), 7.24 - 7.18 (m, 2H, Ar-2,6-H), 7.07 - 7.01 (m, 2H, Ar-3,5-H), 6.51 (d, *J* = 7.0 Hz, 1H, -SO_2_NH-), 5.79 (d, *J* = 9.8 Hz, 1H, 6’-H), 5.35 (dt, *J* = 28.3, 9.5 Hz, 2H, -CH_2_-), 4.34 - 4.08 (m, 6H, 3’,4’-H, morpholine-2,6-H), 3.98 (ddd, *J* = 9.8, 4.9, 2.1 Hz, 1H, 2’-H), 3.86 (s, 4H, morpholine-3,5-H), 2.65 (s, 1H, 5’-H), 2.15 (s, 3H, -COCH_3_), 2.09 (s, 3H, -COCH_3_), 2.04 (s, 3H, -COCH_3_); ^13^C NMR (100 MHz, CDCl_3_) *δ*: 171.56, 170.62, 169.43, 153.44, 152.34, 150.23, 139.02, 138.30, 135.45, 128.52, 126.90, 119.10, 81.02, 74.95, 73.22, 67.72, 66.95, 61.76, 60.47, 58.75, 20.79, 20.72, 20.66; HRMS (ESI): Calculated for C_27_H_30_N_6_O_10_ClS [M-H]^-^: 665.14272, found: 665.14459. **C9** was dissolved in a mixed solution of DCM and MeOH, and a single crystal of **C9** were obtained by slow crystallized at RT. Its fine structure was further confirmed by X-ray spectroscopy (Figure 1), colorless crystal. Crystal data for C_27_H_31_ClN_6_O_10_S (M = 667.09 g/mol): orthorhombic, space group P2_1_2_1_2_1_ a = 11.1236 (2) Å, b = 16.5869 (3) Å, c = 17.7649 (4) Å, *α* = 90 °, *β* = 90 °, *γ* = 90 °, V =	3277.73 (11) Å3, Z =	4, T = 273.15 K, *μ*(CuK*α*) = 1.54178 mm^-1^, ρ_calc_ = 1.352 g/cm^3^, 36847 reflections measured (7.292° ≤ 2Θ ≤ 136.696°),	5991 unique (Rint = 0.0441, Rsigma = 0.0357) which were used in all calculations. The final R_1_ was 0.0538 (I > 2σ(I)) and wR_2_ was 0.1579 (all data).

*2-(acetoxymethyl)-5-((3-chlorophenyl)sulfonamido)-6-(6-morpholino-9H-purin-9-yl)tetrahydro-2H-pyran-3,4-diyl diacetate* (**C10**). White solid; m. p. 124.5-125.3 °C; yield 56.2%; ^1^H NMR (400 MHz, CDCl_3_) *δ*: 8.25 (s, 1H, purine-8-H), 7.79 (s, 1H, purine-2-H), 7.31 - 7.20 (m, 4H, Ar-2,3,4,5-H), 7.09 (t, *J* = 7.8 Hz, 1H, -SO_2_NH-), 6.14 (s, 1H, 6’-H), 5.74 (d, *J* = 9.7 Hz, 1H, 4’-H), 5.31 (d, *J* = 9.4 Hz, 2H, -CH_2_-), 4.41 - 4.07 (m, 6H, 3,2’-H, morpholine-2,6-H), 3.90 (d, *J* = 31.4 Hz, 5H, 5’-H, morpholine-3,5-H), 2.10 - 2.06 (m, 6H, -COCH_3_), 1.86 (s, 3H, -COCH_3_); ^13^C NMR (100 MHz, CDCl_3_) *δ*: 171.36, 170.58, 169.36, 153.64, 152.39, 150.26, 142.00, 135.30, 134.66, 132.20, 129.71, 125.71, 123.92, 119.10, 81.19, 75.28, 73.06, 67.62, 67.03, 61.70, 58.93, 20.74, 20.68, 20.64; HRMS (ESI): Calculated for C_27_H_30_N_6_O_10_ClS [M-H]^-^: 665.14272, found: 665.14471.

*2-(acetoxymethyl)-6-(2-amino-6-morpholino-9H-purin-9-yl)-5-((3-chlorophenyl) sulfonamido)tetrahydro-2H-pyran-3,4-diyl diacetate* (**C11**). White solid; m. p. 126.3-127.1 °C; yield 58.9%; ^1^H NMR (400 MHz, CDCl_3_) *δ*: 7.66 (s, 1H, purine-8-H), 7.53 - 7.44 (m, 2H, Ar-6,5-H), 7.37 (d, *J* = 8.3 Hz, 1H, Ar-2-H), 7.29 (d, *J* = 8.1 Hz, 1H, Ar-3-H), 6.86 (s, 1H, -SO_2_NH-), 5.67 (d, *J* = 9.5 Hz, 1H, 6’-H), 5.33 - 5.20 (m, 2H, -CH_2_-), 4.21 (ddd, *J* = 14.4, 12.6, 3.5 Hz, 6H, 4’,3’-H, morpholine-2,6-H), 3.95 (ddd, *J* = 8.8, 7.8, 6.9 Hz, 3H, 2’-H, -NH_2_-), 3.94 - 3.63 (m, 5H, 5’-H, morpholine-3,5-H), 2.07 (d, *J* = 9.4 Hz, 6H, -COCH_3_), 1.81 (s, 3H, -COCH_3_); ^13^C NMR (100 MHz, CDCl_3_) *δ*: 170.53, 170.13, 169.50, 153.92, 142.86, 134.91, 133.14, 132.32, 130.22, 125.84, 124.27, 117.73, 114.84, 113.77, 82.50, 75.51, 72.43, 67.73, 66.84, 61.64, 59.18, 20.71, 20.64, 20.24; HRMS (ESI): Calculated for C_27_H_31_ClN_7_O_10_S [M-H]^-^: 680.15361, found: 680.15361.

*2-(acetoxymethyl)-5-((4-methoxyphenyl)sulfonamido)-6-(6-morpholino-9H-purin-9-yl)tetrahydro-2H-pyran-3,4-diyl diacetate* (**C12**). White solid; m. p. 127.4-128.2 °C; yield 65.2%; ^1^H NMR (400 MHz, CDCl3) *δ*: 8.25 (s, 1H, purine-8-H), 7.81 (s, 1H, purine-2-H), 7.21 (d, *J* = 8.9 Hz, 2H, Ar-2,6-H), 6.56 (d, *J* = 9.0 Hz, 2H, Ar-3,5-H), 5.87 (d, *J* = 8.5 Hz, 1H, -SO_2_NH-), 5.74 (d, *J* = 9.8 Hz, 1H, 6’-H), 5.31 (dd, *J* = 6.0, 3.7 Hz, 2H, 4’, 3’-H), 4.37 - 4.07 (m, 7H, 2’-H, -CH_2_-, and morpholine-2,6-H), 3.97 - 3.92 (m, 1H, 5’-H), 3.85 (t, *J* = 4.6 Hz, 4H, morpholine-3,5-H), 3.79 (s, 3H, Ar-OCH_3_), 2.08 (s, 6H, -COCH_3_), 2.05 (s, 3H, -COCH_3_); ^13^C NMR (100 MHz, CDCl_3_) *δ*: 171.50, 170.61, 169.39, 162.31, 153.55, 152.30, 150.44, 135.63, 132.07, 127.77, 119.27, 113.54, 81.32, 75.06, 73.20, 67.68, 66.98, 61.75, 58.58, 55.57, 20.75, 20.74, 20.65; HRMS (ESI): Calculated for C_28_H_33_N_6_O_11_S [M-H]^-^: 661.19225, found: 661.19482.

*2-(acetoxymethyl)-6-(2-fluoro-6-morpholino-9H-purin-9-yl)-5-((4-methoxyphenyl) sulfonamido)tetrahydro-2H-pyran-3,4-diyl diacetate* (**C13**). White solid; m. p. 129.3-130.0 °C; yield 63.7%; ^1^H NMR (400 MHz, CDCl_3_) *δ*: 7.38 (s, 1H, purine-8-H), 7.17 (d, *J* = 8.8 Hz, 2H, Ar-2,6-H), 6.56 (d, *J* = 8.9 Hz, 2H, Ar-3,5-H), 5.80(s, 1H, -SO_2_NH-), 5.46 (d, *J* = 9.6 Hz, 1H, 6’-H), 5.26 (dd, *J* = 12.6, 9.5 Hz, 2H, -CH_2_-), 4.25 - 4.22 (m, 1H, 4’-H), 4.10 (dd, *J* = 12.5, 2.0 Hz, 4H, 3’-H, Ar-OCH_3_), 3.97 (s, 1H, 2’-H), 3.87 (dd, *J* = 9.5, 3.0 Hz, 1H, 5’-H), 3.81 (s, 2H, morpholine-2,6-H), 3.78 (s, 2H, morpholine-2,6-H), 3.71 - 3.68 (m, 4H, morpholine-3,5-H), 2.11 (s, 3H, -COCH_3_), 2.05 (d, *J* = 8.0 Hz, 6H, -COCH_3_); ^13^C NMR (100 MHz, CDCl_3_) *δ*: 171.36, 170.57, 169.39, 162.40, 158.32, 153.48, 152.43, 150.06, 132.92, 131.46, 127.86, 113.66, 100.00, 81.45, 75.32, 73.57, 67.77, 66.99, 66.92, 61.83, 55.51, 44.97, 20.87, 20.73, 20.63; ^19^F NMR (376 MHz, CDCl_3_) *δ*: -50.57; HRMS (ESI): Calculated for C_28_H_33_FN_6_O_11_S [M-H]^-^: 679.18283, found: 679.18463.

*2-(acetoxymethyl)-6-(2-amino-6-morpholino-9H-purin-9-yl)-5-((4-methoxyphenyl) sulfonamido)tetrahydro-2H-pyran-3,4-diyl diacetate* (**C14**). White solid; m. p. 126.1-127.0 °C; yield 66.2%; ^1^H NMR (400 MHz, CDCl_3_) *δ*: 7.38 (s, 1H, purine-8-H), 7.23 (s, 1H, Ar-2-H), 7.21 (s, 1H, Ar-6-H), 6.55 (s, 1H, Ar-3-H), 6.53 (s, 1H, Ar-5-H), 5.51 (d, *J* = 9.7 Hz, 1H, -SO_2_NH-), 5.29 (d, *J* = 9.8 Hz, 1H, 6’-H), 5.23 - 5.19 (m, 1H, 4’-H), 4.74 (s, 2H, -CH_2_-), 4.17 - 4.00 (m, 7H, -NH_2_, 3’-H, morpholine-2,6-H), 3.89 (ddd, *J* = 9.9, 4.9, 2.1 Hz, 1H, 2’-H), 3.73 (s, 8H, 5’-H, Ar-OCH_3_, morpholine-3,5-H), 2.02 (s, 3H, -COCH_3_), 2.00 (s, 3H, -COCH_3_), 1.99 (s, 3H, -COCH_3_); ^13^C NMR (100 MHz, CDCl3) *δ*: 170.27, 169.62, 168.44, 161.24, 158.03, 152.87, 151.26, 131.95, 131.06, 126.77, 113.19, 112.61, 80.12, 73.89, 72.38, 66.82, 65.95, 60.85, 57.58, 54.50, 19.81, 19.71, 19.60; HRMS (ESI): Calculated for C_28_H_34_N_7_O_11_S [M-H]^-^: 676.20315, found: 676.20477.

*2-(acetoxymethyl)-5-((4-bromophenyl)sulfonamido)-6-(6-morpholino-9H-purin-9-yl)tetrahydro-2H-pyran-3,4-diyl diacetate* (**C15**). White solid; m. p. 130.4-131.2 °C; yield 65.2%; ^1^H NMR (500 MHz, CDCl_3_) *δ*: 8.23 (s, 1H, purine-8-H), 7.78 (s, 1H, purine-2-H), 7.18 (d, *J* = 8.7 Hz, 2H, Ar-3,5-H), 7.11 (d, *J* = 8.7 Hz, 2H, Ar-2,6-H), 6.55 (d, *J* = 9.3 Hz, 1H, -SO_2_NH-), 5.75 (d, *J* = 9.8 Hz, 1H, 6’-H), 5.35 (dd, *J* = 17.3, 7.6 Hz, 1H, 4’-H), 5.31 - 5.25 (m, 1H, -CH_2_-), 4.36 - 4.13 (m, 5H, -CH_2_-, morpholine-2,6-H), 4.07 (dd, *J* = 12.6, 2.1 Hz, 1H, 3’-H), 3.95 (ddd, *J* = 10.0, 5.0, 2.1 Hz, 1H, 2’-H), 3.82 (s, 4H, morpholine-3,5-H), 2.68 (s, 1H, 5’-H), 2.09 (s, 3H, -COCH_3_), 2.05 (s, 3H, -COCH_3_), 2.01 (s, 3H, -COCH_3_); ^13^C NMR (125 MHz, CDCl_3_) *δ*: 171.61, 170.70, 169.51, 153.53, 152.39, 150.28, 139.65, 135.59, 131.57, 127.08, 126.94, 119.19, 81.11, 74.99, 73.26, 67.81, 67.04, 61.83, 58.73, 20.84, 20.80, 20.74; HRMS (ESI): Calculated for C_27_H_32_BrN_6_O_10_S [M+H]^+^: 711.10785, found: 711.10663.

*2-(acetoxymethyl)-5-((3-bromophenyl)sulfonamido)-6-(6-morpholino-9H-purin-9-yl)tetrahydro-2H-pyran-3,4-diyl diacetate* (**C16**). White solid; m. p. 129.1-129.8 °C; yield 68.3%; ^1^H NMR (500 MHz, CDCl_3_) *δ*: 8.25 (s, 1H, purine-8-H), 7.79 (s, 1H, purine-2-H), 7.43 (dd, *J* = 3.5, 1.7 Hz, 2H, Ar-2,4-H), 7.27 (s, 1H, Ar-6-H), 7.04 (d, *J* = 8.2 Hz, 1H, Ar-5-H), 6.01 (d, *J* = 8.9 Hz, 1H, -SO_2_NH-), 5.75 (d, *J* = 9.7 Hz, 1H, 6’-H), 5.31 (dd, *J* = 6.4, 3.5 Hz, 2H, -CH_2_-), 4.35 - 4.11 (m, 7H, 2,3,4’-H, morpholine-2,6-H), 3.94 (s, 1H, 5’-H), 3.86 (s, 4H, morpholine-3,5-H), 2.09 (s, 3H, -COCH_3_), 2.07 (s, 3H, -COCH_3_), 2.06 (s, 3H, -COCH_3_); ^13^C NMR (125 MHz, CDCl_3_) *δ*: 171.31, 170.67, 169.49, 153.72, 152.44, 150.32, 142.56, 135.74, 134.96, 129.90, 128.56, 124.28, 122.46, 119.26, 81.25, 75.06, 73.23, 67.89, 67.11, 61.81, 58.50, 20.79, 20.76, 20.71; HRMS (ESI): Calculated for C_27_H_30_BrN_6_O_10_S [M-H]^-^: 709.09220, found: 709.093933.

*2-(acetoxymethyl)-5-((2-bromophenyl)sulfonamido)-6-(6-morpholino-9H-purin-9-yl)tetrahydro-2H-pyran-3,4-diyl diacetate* (**C17**). White solid; m. p. 145.2-146.1 °C; yield 64.8%; ^1^H NMR (400 MHz, CDCl_3_) *δ*: 8.27 (s, 1H, purine-8-H), 7.73 (s, 1H, purine-2-H), 7.65 (dd, *J* = 7.8, 1.4 Hz, 1H, Ar-3-H), 7.32 (d, *J* = 7.8 Hz, 1H, Ar-4-H), 7.21 (t, *J* = 7.2 Hz, 1H, Ar-6-H), 7.16 - 7.11 (m, 1H, Ar-5-H), 6.00 (d, *J* = 9.3 Hz, 1H, -SO_2_NH-), 5.77 (d, *J* = 9.8 Hz, 1H, 6’-H), 5.33 (dd, *J* = 22.2, 9.8 Hz, 2H, -CH_2_-), 4.43 - 4.10 (m, 7H, 2,3,4’-H, morpholine-2,6-H), 3.93 (dd, *J* = 9.9, 2.8 Hz, 1H, 5’-H), 3.84 (t, *J* = 4.6 Hz, 4H, morpholine-3,5-H), 2.09 - 2.02 (m, 9H, -COCH_3_); ^13^C NMR (100 MHz, CDCl_3_) *δ*: 152.44, 135.72, 134.36, 132.86, 128.96, 127.22, 119.04, 77.37, 77.05, 76.73, 75.10, 72.76, 67.83, 67.06, 58.30, 20.74, 20.65, 20.57; HRMS (ESI): Calculated for C_27_H_31_BrN_6_O_10_S [M+H]^+^: 711.10785, found: 711.10638.

*2-(acetoxymethyl)-6-(6-morpholino-9H-purin-9-yl)-5-(phenylsulfonamido) tetrahydro-2H-pyran-3,4-diyl diacetate* (**C18**). White solid; m. p. 123.4-124.2 °C; yield 69.4%; ^1^H NMR (400 MHz, CDCl_3_) *δ*: 8.25 (s, 1H, purine-8-H), 7.81 (s, 1H, purine-2-H), 7.32 (dd, *J* = 6.1, 3.0 Hz, 3H, Ar-2,4,6-H), 7.16 (t, *J* = 7.8 Hz, 2H, Ar-3,5-H), 5.92 (d, *J* = 8.9 Hz, 1H, -SO_2_NH-), 5.75 (d, *J* = 9.7 Hz, 1H, 6’-H), 5.33 - 5.27 (m, 2H, -CH_2_-), 4.35 - 4.09 (m, 7H, 2,3,4’-H, morpholine-2,6-H), 3.96 - 3.92 (m, 1H, 5’-H), 3.86 (t, *J* = 4.7 Hz, 4H, morpholine-3,5-H), 2.06 (s, 3H, -COCH_3_), 1.98 (s, 3H, -COCH_3_), 1.92 (s, 3H, -COCH_3_); ^13^C NMR (100 MHz, CDCl_3_) *δ*: 171.32, 170.60, 169.38, 153.63, 152.29, 150.40, 140.48, 135.53, 132.09, 128.53, 125.73, 119.25, 81.33, 75.21, 73.07, 67.70, 67.08, 61.73, 58.80, 20.75, 20.64, 20.61; HRMS (ESI): Calculated for C_27_H_30_N_6_O_10_S [M-H]^-^: 631.18169, found: 631.18286.

*2-(acetoxymethyl)-6-(2-fluoro-6-morpholino-9H-purin-9-yl)-5-(phenylsulfonamido) tetrahydro-2H-pyran-3,4-diyl diacetate* (**C19**). White solid; m. p. 124.2-125.0 °C; yield 57.3%; ^1^H NMR (400 MHz, CDCl_3_) *δ*: 7.34 (s, 1H, purine-8-H), 7.22 (d, *J* = 8.1 Hz, 3H, Ar-2,6,4-H), 7.05 (t, *J* = 7.8 Hz, 2H, Ar-3,5-H), 6.13 (s, 1H, -SO_2_NH-), 5.47 (d, *J* = 9.6 Hz, 1H, 6’-H), 5.29 (t, *J* = 9.7 Hz, 1H, 4’-H), 5.18 (t, *J* = 9.7 Hz, 1H, 3’-H), 4.21 - 4.16 (m, 1H, 2’-H), 4.05 (d, *J* = 10.5 Hz, 2H, -CH_2_-), 3.88 - 3.84 (m, 1H, 5’-H), 3.75 (s, 2H, morpholine-2-H), 3.71 (s, 2H, morpholine-6-H), 3.64 (d, *J* = 3.7 Hz, 4H, morpholine-3,5-H), 2.04 - 1.98 (m, 9H, -COCH_3_); ^13^C NMR (100 MHz, CDCl_3_) *δ*: 171.33, 170.62, 169.42, 158.27, 153.46, 152.40, 140.05, 132.99, 132.01, 128.47, 125.68, 113.65, 81.34, 75.01, 73.67, 67.90, 67.08, 66.91, 61.87, 58.65, 44.93, 20.90, 20.70, 20.64; ^19^F NMR (376 MHz, CDCl3) *δ*: -49.03; HRMS (ESI): Calculated for C_27_H_30_FN_6_O_10_S [M-H]^-^: 649.17227, found: 649.17358.

*2-(acetoxymethyl)-6-(2-chloro-6-morpholino-9H-purin-9-yl)-5-(phenylsulfonamido) tetrahydro-2H-pyran-3,4-diyl diacetate* (**C20**). White solid; m. p. 131.1-132.0 °C; yield 65.4%; ^1^H NMR (500 MHz, CDCl_3_) *δ*: 7.79 (s, 1H, purine-8-H), 7.36 (dd, *J* = 8.3, 1.1 Hz, 2H, Ar-2,6-H), 7.29 (d, *J* = 7.5 Hz, 1H, Ar-4-H), 7.18 (t, *J* = 7.8 Hz, 2H, Ar-3,5-H), 5.92 (d, *J* = 8.6 Hz, 1H, -SO_2_NH-), 5.67 (d, *J* = 9.7 Hz, 1H, 6’-H), 5.33 (t, *J* = 9.8 Hz, 1H, -CH_2_-), 5.24 (t, *J* = 9.8 Hz, 1H, -CH_2_-), 4.16 (ddd, *J* = 17.4, 8.9, 4.1 Hz, 5H, 4’-H, morpholine-2,6-H), 3.96 - 3.93 (m, 1H, 3’-H), 3.82 (s, 5H, 2’-H, morpholine-3,5-H), 2.38 (s, 1H, 5’-H), 2.06 (s, 3H, -COCH_3_), 2.04 (s, 3H, -COCH_3_), 1.96 (s, 3H, -COCH_3_); ^13^C NMR (125 MHz, CDCl_3_) *δ*: 171.62, 170.73, 169.56, 153.87, 153.69, 151.72, 140.77, 136.04, 132.13, 128.58, 125.78, 118.00, 81.20, 75.09, 72.91, 67.88, 67.03, 61.85, 58.68, 20.84, 20.76, 20.73; HRMS (ESI): Calculated for C_27_H_30_ClN_6_O_10_S [M-H]^-^: 665.14272, found: 665.14502.

*2-(acetoxymethyl)-6-(6-morpholino-9H-purin-9-yl)-5-((2,4,6-trimethylphenyl) sulfonamido)tetrahydro-2H-pyran-3,4-diyl diacetate* (**C21**). White solid; m. p. 129.5-130.4 °C; yield 66.3%; ^1^H NMR (500 MHz, CDCl_3_) *δ*: 8.21 (s, 1H, purine-8-H), 7.76 (s, 1H, purine-2-H), 6.57 (s, 2H, Ar-3,5-H), 5.71 (d, *J* = 9.8 Hz, 1H, -SO_2_NH-), 5.45 (d, *J* = 8.9 Hz, 1H, 6’-H), 5.30 - 5.27 (m, 2H, -CH_2_-), 4.29 - 4.17 (m, 5H, 4’-H, morpholine-2,6-H), 4.11 - 4.07 (m, 1H, 3’-H), 3.89 (dd, *J* = 6.4, 3.5 Hz, 1H, 2’-H), 3.80 (s, 5H, 5’-H, morpholine-3,5-H), 2.34 (s, 6H, Ar-CH_3_), 2.16 (s, 3H, Ar-CH_3_), 2.06 (s, 3H, -COCH_3_), 2.04 (s, 3H, -COCH_3_), 1.97 (s, 3H, -COCH_3_); ^13^C NMR (125 MHz, CDCl_3_) *δ*: 171.70, 170.67, 169.41, 153.48, 152.37, 150.44, 141.62, 137.57, 135.51, 135.01, 131.45, 119.35, 81.41, 75.16, 73.16, 67.72, 67.06, 61.81, 58.63, 22.88, 21.07, 20.82, 20.72, 20.57; HRMS (ESI): Calculated for C_30_H_37_N_6_O_10_S [M-H]^-^: 673.22864, found: 673.23132.

*2-(acetoxymethyl)-6-(2-amino-6-morpholino-9H-purin-9-yl)-5-((2,4,6-trimethylphenyl)sulfonamido)tetrahydro-2H-pyran-3,4-diyl diacetate* (**C22**). White solid; m. p. 135.1-136.0 °C; yield 55.3%; ^1^H NMR (400 MHz, CDCl_3_) *δ*: 7.45 (s, 1H, purine-8-H), 6.62 (s, 1H, Ar-3-H), 5.49 (d, *J* = 9.8 Hz, 1H, Ar-5-H), 5.40 (d, *J* = 9.5 Hz, 1H, -SO_2_NH-), 5.25 (d, *J* = 9.7 Hz, 2H, 6’,4’-H), 4.58 (s, 2H, -CH_2_-), 4.19 (dd, *J* = 12.4, 5.0 Hz, 3H, 3’-H, -NH_2_), 4.08 (d, *J* = 12.5 Hz, 4H, morpholine-2,6-H), 3.85 (s, 1H, 2’-H), 3.76 (s, 5H, 5’-H, morpholine-3,5-H), 2.38 (s, 6H, Ar-CH_3_), 2.17 (s, 3H, Ar-CH_3_), 2.05 (d, *J* = 5.8 Hz, 6H, -COCH_3_), 1.97 (s, 3H, -COCH_3_); ^13^C NMR (100 MHz, CDCl_3_) *δ*: 171.36, 170.69, 169.42, 158.91, 153.87, 152.24, 141.46, 137.65, 135.14, 133.09, 131.46, 114.22, 81.16, 77.27, 74.81, 73.27, 67.94, 67.02, 61.89, 58.29, 22.80, 21.02, 20.76, 20.63, 20.45; HRMS (ESI): Calculated for C_30_H_38_N_7_O_10_S [M-H]^-^: 688.23954, found: 688.24231.

*2-(acetoxymethyl)-6-(2-chloro-6-morpholino-9H-purin-9-yl)-5-((2,4,6-trimethylphenyl)sulfonamido)tetrahydro-2H-pyran-3,4-diyl diacetate* (**C23**). White solid; m. p. 130.9-131.8 °C; yield 64.8%; ^1^H NMR (500 MHz, CDCl_3_) *δ*: 7.76 (s, 1H, purine-8-H), 6.60 (s, 2H, Ar-3,5-H), 5.67 (dd, *J* = 14.7, 9.5 Hz, 2H, -SO_2_NH-, 6’-H), 5.39 (t, *J* = 9.9 Hz, 1H, -CH_2_-), 5.26 (t, *J* = 9.8 Hz, 1H, -CH_2_-), 4.76 (s, 1H, 4’-H), 4.22 - 4.10 (m, 5H, 3’-H, morpholine-2,6-H), 3.95 - 3.91 (m, 1H, 2’-H), 3.77 (d, *J* = 38.3 Hz, 5H, 5’-H, morpholine-3,5-H), 2.37 (s, 6H, Ar-CH_3_), 2.16 (s, 3H, Ar-CH_3_), 2.09 (s, 3H, -COCH_3_), 2.02 (d, *J* = 2.3 Hz, 6H, -COCH_3_); ^13^C NMR (125 MHz, CDCl_3_) *δ*: 172.05, 170.70, 169.50, 153.87, 153.46, 151.55, 141.49, 137.41, 135.75, 135.37, 131.36, 118.04, 81.17, 75.07, 73.04, 67.91, 66.96, 61.92, 60.57, 58.68, 22.77, 21.11, 20.85, 20.71, 20.61; HRMS (ESI): Calculated for C_30_H_36_ClN_6_O_10_S [M-H]^-^: 707.18967, found: 707.19116.

*2-(acetoxymethyl)-5-((4-methylphenyl)sulfonamido)-6-(6-morpholino-9H-purin-9-yl)tetrahydro-2H-pyran-3,4-diyl diacetate* (**C24**). White solid; m. p. 129.3-130.1 °C; yield 68.2%; ^1^H NMR (400 MHz, CDCl_3_) *δ*: 8.18 (s, 1H, purine-8-H), 7.85 (s, 1H, purine-2-H), 7.14 (d, *J* = 8.3 Hz, 2H, Ar-2,6-H), 6.83 (d, *J* = 8.2 Hz, 2H, Ar-3,5-H), 6.52 (s, 1H, -SO_2_NH-), 5.74 (d, *J* = 9.8 Hz, 1H, 6’-H), 5.25 (dt, *J* = 35.3, 9.6 Hz, 2H, -CH_2_-), 4.27 - 4.10 (m, 5H, 4’-H, morpholine-2,6-H), 4.02 (dd, *J* = 12.5, 1.9 Hz, 1H, 3’-H), 3.92 (ddd, *J* = 9.9, 4.8, 2.0 Hz, 1H, 2’-H), 3.77 (t, *J* = 4.5 Hz, 4H, morpholine-3,5-H), 2.92 (s, 1H, 5’-H), 2.21 (s, 3H, Ar-CH_3_), 1.98 (d, *J* = 7.9 Hz, 6H, -COCH_3_), 1.92 (s, 3H, -COCH_3_); ^13^C NMR (100 MHz, CDCl_3_) *δ*: 171.36, 170.66, 169.49, 153.61, 152.22, 150.48, 142.69, 137.89, 136.16, 128.97, 125.65, 119.33, 81.21, 74.73, 73.24, 67.91, 66.99, 61.78, 58.16, 21.50, 20.70, 20.62, 20.61; HRMS (ESI): Calculated for C_28_H_33_N_6_O_10_S [M-H]^-^: 645.19734, found: 645.19891.

*2-(acetoxymethyl)-6-(2-chloro-6-morpholino-9H-purin-9-yl)-5-((4-methylphenyl) sulfonamido)tetrahydro-2H-pyran-3,4-diyl diacetate* (**C25**). White solid; m. p. 128.4-129.2 °C; yield 65.9%; ^1^H NMR (400 MHz, CDCl_3_) *δ*: 7.86 (s, 1H, purine-8-H), 7.27 (d, *J* = 8.6 Hz, 2H, Ar-2,6-H), 6.99 (d, *J* = 8.1 Hz, 2H, Ar-3,5-H), 5.79 (d, *J* = 9.1 Hz, 1H, -SO_2_NH-), 5.68 (d, *J* = 9.7 Hz, 1H, 6’-H), 5.30 (dt, *J* = 24.7, 9.6 Hz, 2H, -CH_2_-), 4.69 (s, 1H, 4’-H), 4.40 - 4.08 (m, 5H, 3’-H, morpholine-2,6-H), 3.94 (ddd, *J* = 47.6, 23.9, 20.9 Hz, 6H, 2’,5’-H, morpholine-3,5-H), 2.31 (s, 3H, Ar-CH_3_), 2.08 (d, *J* = 9.8 Hz, 6H, -COCH_3_), 1.99 (s, 3H, -COCH_3_); ^13^C NMR (100 MHz, CDCl_3_) *δ*: 171.59, 170.64, 169.46, 153.81, 153.61, 151.78, 142.93, 137.77, 136.26, 129.06, 125.76, 118.05, 81.28, 74.96, 72.87, 67.78, 66.93, 61.75, 58.29, 21.57, 20.75, 20.68, 20.62; HRMS (ESI): Calculated for C_28_H_32_ClN_6_O_10_S [M-H]^-^: 679.15837, found: 679.15961.

*2-(acetoxymethyl)-6-(6-morpholino-9H-purin-9-yl)-5-((4-(trifluoromethyl)phenyl) sulfonamido)tetrahydro-2H-pyran-3,4-diyl diacetate* (**C26**). White solid; m. p. 127.4-128.2 °C; yield 68.1%; ^1^H NMR (400 MHz, CDCl_3_) *δ*: 8.22 (s, 1H, purine-8-H), 7.82 (s, 1H, purine-2-H), 7.41 (s, 2H, Ar-3,5-H), 7.36 (s, 2H, Ar-2,6-H), 6.50 (d, *J* = 8.1 Hz, 1H, -SO_2_NH-), 5.78 (d, *J* = 9.8 Hz, 1H, 6’-H), 5.34 (t, *J* = 9.8 Hz, 1H, -CH_2_-), 5.28 (t, *J* = 9.7 Hz, 1H, -CH_2_-), 4.21 (dd, *J* = 12.6, 5.0 Hz, 3H, 4’-H, morpholine-2,6-H), 4.08 (dd, *J* = 12.6, 1.9 Hz, 1H, 3’-H), 3.97 - 3.94 (m, 1H, 2’-H), 3.74 (d, *J* = 52.0 Hz, 6H, morpholine-2,6-H, morpholine-3,5-H), 2.49 (s, 1H, 5’-H), 2.05 (s, 3H, -COCH_3_), 2.02 (d, *J* = 6.3 Hz, 6H, -COCH_3_); ^13^C NMR (100 MHz, CDCl_3_) *δ*: 171.47, 170.60, 169.40, 153.54, 152.40, 150.37, 143.99, 135.60, 133.91, 133.58, 126.18, 125.54, 125.57, 124.40, 121.68, 119.13, 81.09, 75.01, 73.15, 67.74, 67.10, 66.90, 61.69, 58.59, 20.71, 20.64; ^19^F NMR (376 MHz) *δ*: -63.00; HRMS (ESI): Calculated for C_28_H_31_F_3_N_6_O_10_S [M-H]^-^: 699.16907, found: 699.17047.

*2-(acetoxymethyl)-6-(2-chloro-6-morpholino-9H-purin-9-yl)-5-((4-(trifluoromethyl) phenyl)sulfonamido)tetrahydro-2H-pyran-3,4-diyl diacetate* (**C27**). White solid; m. p. 120.3-121.2 °C; yield 65.7%; ^1^H NMR (500 MHz, CDCl_3_) *δ*: 7.79 (s, 1H, purine-8-H), 7.50 (d, *J* = 8.3 Hz, 2H, Ar-3,5-H), 7.43 (d, *J* = 8.5 Hz, 2H, Ar-2,6-H), 6.21 (d, *J* = 9.2 Hz, 1H, -SO_2_NH-), 5.69 (d, *J* = 9.8 Hz, 1H, 6’-H), 5.37 (t, *J* = 9.9 Hz, 1H, -CH_2_-), 5.25 (t, *J* = 9.8 Hz, 1H, -CH_2_-), 4.65 (s, 1H, 4’-H), 4.29 - 4.08 (m, 5H, 3’-H, morpholine-2,6-H), 3.98 - 3.94 (m, 1H, 2’-H), 3.76 (d, *J* = 7.0 Hz, 4H, morpholine-3,5-H), 2.40 (s, 1H, 5’-H), 2.08 (s, 3H, -COCH_3_), 2.05 (s, 3H, -COCH_3_), 2.01 (s, 3H, -COCH_3_); ^13^C NMR (125 MHz, CDCl_3_) *δ*: 171.72, 170.70, 169.54, 154.02, 153.54, 151.70, 144.18, 135.92, 134.01, 133.75, 126.31, 125.70, 125.67, 124.19, 122.02, 117.89, 81.13, 75.12, 72.93, 67.86, 66.88, 61.78, 58.70, 2082, 20.76, 20.74; ^19^F NMR (470 MHz) *δ*: -62.91; HRMS (ESI): Calculated for C_28_H_29_ClF_3_N_6_O_10_S [M-H]^-^: 733.13010, found: 733.13245.

*2-(acetoxymethyl)-6-(2-fluoro-6-morpholino-9H-purin-9-yl)-5-((4-(trifluoromethyl) phenyl)sulfonamido)tetrahydro-2H-pyran-3,4-diyl diacetate* (**C28**). White solid; m. p. 121.3-122.1 °C; yield 67.3%; ^1^H NMR (500 MHz, CDCl_3_) *δ*: 7.78 (s, 1H, purine-8-H), 7.48 (d, *J* = 8.3 Hz, 2H, Ar-3,5-H), 7.42 (d, *J* = 8.5 Hz, 2H, Ar-2,6-H), 6.46 (d, *J* = 9.6 Hz, 1H, -SO_2_NH-), 5.72 (d, *J* = 9.8 Hz, 1H, 6’-H), 5.38 (t, *J* = 9.8 Hz, 1H, -CH_2_-), 5.26 (t, *J* = 9.8 Hz, 1H, -CH_2_-), 4.70 (s, 1H, 4’-H), 4.29 - 4.04 (m, 5H, 3’-H, morpholine-2,6-H), 3.96 (ddd, *J* = 10.1, 5.0, 2.1 Hz, 1H, 2’-H), 3.77 (d, *J* = 43.0 Hz, 4H, morpholine-3,5-H), 2.37 (s, 1H, 5’-H), 2.07 (s, 3H, -COCH_3_), 2.02 (s, 3H, -COCH_3_), 2.01 (s, 3H, -COCH_3_); ^13^C NMR (125 MHz, CDCl_3_) *δ*: 171.79, 170.69, 169.57, 159.54, 157.89, 154.58, 154.42, 152.25, 152.09, 144.28, 136.01, 133.96, 133.69, 126.27, 125.68, 125.65, 124.20, 122.02, 117.36, 81.08, 74.93, 73.06, 67.85, 66.84, 61.80, 58.66, 20.76, 20.74, 20.69; ^19^F NMR (470 MHz, ) *δ*: -50.05, -62.90; HRMS (ESI): Calculated for C_28_H_29_F_4_N_6_O_10_S [M-H]^-^: 717.15965, found: 717.16205.

*2-(acetoxymethyl)-6-(6-morpholino-9H-purin-9-yl)-5-((3-(trifluoromethyl)phenyl) sulfonamido)tetrahydro-2H-pyran-3,4-diyl diacetate* **(C29)**. White solid; m. p. 121.0-121.8 °C; yield 65.6%; ^1^H NMR (400 MHz, CDCl_3_) *δ*: 8.19 (s, 1H, purine-8-H), 7.76 (s, 1H, purine-2-H), 7.57 (s, 1H, Ar-2-H), 7.46 (d, *J* = 7.8 Hz, 1H, Ar-4-H), 7.41 (d, *J* = 8.0 Hz, 1H, Ar-6-H), 7.21 - 7.15 (m, 1H, Ar-5-H), 6.68 (s, 1H, -SO_2_NH-), 5.75 (d, *J* = 9.8 Hz, 1H, 6’-H), 5.28 (dd, *J* = 20.3, 9.8 Hz, 2H, -CH_2_-), 4.22 - 4.01 (m, 6H, 4’, 3’-H, morpholine-2,6-H), 3.93 - 3.89 (m, 1H, 2’-H), 3.77 (t, *J* = 4.5 Hz, 4H, morpholine-3,5-H), 2.49 (s, 1H, 5’-H), 2.01 (s, 6H, -COCH_3_), 1.97 (s, 3H, -COCH_3_); ^13^C NMR (100 MHz, CDCl_3_) *δ*: 171.55, 170.62, 169.44, 153.54, 152.40, 150.24, 141.84, 135.55, 131.27, 130.94, 129.17, 128.58 (d, *J* = 17.4 Hz), 124.25, 122.91 (d, *J* = 3.7 Hz), 121.54, 119.07, 80.97, 74.91, 73.20, 67.74, 67.01, 61.70, 58.64, 20.70, 20.67, 20.63; ^19^F NMR (376 MHz, CDCl_3_) *δ*: -62.68; HRMS (ESI): Calculated for C_28_H_30_F_3_N_6_O_10_S [M-H]^-^: 699.16907, found: 699.17114.

*2-(acetoxymethyl)-5-((4-fluorophenyl)sulfonamido)-6-(6-morpholino-9H-purin-9-yl)tetrahydro-2H-pyran-3,4-diyl diacetate* (**C30**). White solid; m. p. 125.9-126.7 °C; yield 67.2%; ^1^H NMR (500 MHz, CDCl_3_) *δ*: 8.19 (s, 1H, purine-8-H), 7.71 (s, 1H, purine-2-H), 7.21 (t, *J* = 6.5 Hz, 2H, Ar-2,6-H), 6.68 (t, *J* = 8.4 Hz, 2H, Ar-3,5-H), 6.22 (d, *J* = 75.6 Hz, 1H, -SO_2_NH-), 5.71 (d, *J* = 9.7 Hz, 1H, 6’-H), 5.27 (dd, *J* = 16.3, 9.7 Hz, 2H, -CH_2_-), 4.12 (ddd, *J* = 35.5, 19.3, 8.5 Hz, 7H, 4’,3’,2’-H, morpholine-2,6-H), 3.94 - 3.89 (m, 1H, 5’-H), 3.79 (s, 4H, morpholine-3,5-H), 2.08 (s, 3H, -COCH_3_), 2.02 (s, 3H, -COCH_3_), 1.97 (s, 3H, -COCH_3_); ^13^C NMR (125 MHz, CDCl_3_) *δ*: 171.59, 170.70, 169.51, 165.37, 163.34, 153.52, 152.37, 150.21, 136.69, 135.50, 128.24, 128.17 119.14, 115.68, 115.60, 115.68, 115.50, 81.13, 75.04, 73.29, 67.80, 66.96, 61.85, 58.93, 20.88, 20.80, 20.73; ^19^F NMR (471 MHz, CDCl_3_) *δ*: -105.24; HRMS (ESI): Calculated for C_27_H_30_FN_6_O_10_S [M-H]^-^: 649.17227, found: 649.17395.

*2-(acetoxymethyl)-6-(2-fluoro-6-morpholino-9H-purin-9-yl)-5-((4-fluorophenyl) sulfonamido)tetrahydro-2H-pyran-3,4-diyl diacetate* (**C31**). White solid; m. p. 124.7-125.5 °C; yield 68.3%; ^1^H NMR (400 MHz, CDCl_3_) *δ*: 7.68 (s, 1H, purine-8-H), 7.27 (dd, *J* = 8.8, 5.0 Hz, 2H, Ar-2,6-H), 6.74 (t, *J* = 8.5 Hz, 2H, Ar-3,5-H), 6.41 (d, *J* = 9.3 Hz, 1H, -SO_2_NH-), 5.68 (d, *J* = 9.7 Hz, 1H, 6’-H), 5.35 (t, *J* = 9.8 Hz, 1H, -CH_2_-), 5.23 (t, *J* = 9.8 Hz, 1H, -CH_2_-), 4.45 (d, *J* = 83.5 Hz, 2H, 4’,3’-H), 4.09 (ddd, *J* = 23.2, 12.6, 3.4 Hz, 4H, morpholine-2,6-H), 3.94 - 3.90 (m, 1H, 2’-H), 3.78 (d, *J* = 14.7 Hz, 4H, morpholine-3,5-H), 2.43 (s, 1H, 5’-H), 2.10 (s, 3H, -COCH_3_), 2.03 (s, 3H, -COCH_3_), 1.96 (s, 3H, -COCH_3_); ^13^C NMR (100 MHz, CDCl_3_) *δ*: 171.72, 170.66, 169.54, 165.53, 163.00, 159.63, 157.57, 154.32 (d, *J* = 20.0 Hz), 151.82 (d, *J* = 19.2 Hz), 136.88, 135.70, 128.14 (d, *J* = 9.3 Hz), 117.16, 115.64, 115.42, 80.91, 74.86, 73.02, 67.72, 66.73, 61.82, 59.00,20.81, 20.71, 20.70; ^19^F NMR (471 MHz, CDCl_3_) *δ*: -49.98, -105.26; HRMS (ESI): Calculated for C_27_H_29_F_2_N_6_O_10_S [M-H]^-^: 667.16284, found: 667.16473.

*2-(acetoxymethyl)-6-(2-chloro-6-morpholino-9H-purin-9-yl)-5-((4-fluorophenyl) sulfonamido)tetrahydro-2H-pyran-3,4-diyl diacetate* (**C32**). White solid; m. p. 141.2-142.0 °C; yield 62.4%; ^1^H NMR (500 MHz, CDCl_3_) *δ*: 7.72 (s, 1H, purine-8-H), 7.34 - 7.30 (m, 2H, Ar-2,6-H), 6.80 - 6.76 (m, 2H, Ar-3,5-H), 6.11 (d, *J* = 8.8 Hz, 1H, -SO_2_NH-), 5.66 (d, *J* = 9.8 Hz, 1H, 6’-H), 5.37 (t, *J* = 9.8 Hz, 1H, -CH_2_-), 5.27 (t, *J* = 9.8 Hz, 1H, -CH_2_-), 4.54 - 4.08 (m, 5H, 4’-H, morpholine-2,6-H), 3.97 - 3.94 (m, 1H, 3’-H), 3.82 (s, 5H, 2’-H, morpholine-3,5-H), 2.54 (s, 1H, 5’-H), 2.13 (s, 3H, -COCH_3_), 2.08 (s, 3H, -COCH_3_), 2.04 (s, 3H, -COCH_3_); ^13^C NMR (125 MHz, CDCl_3_) *δ*: 171.75, 170.74, 169.56, 165.38, 163.36, 153.94, 153.48, 151.48, 136.81, 135.68, 128.27 (d, *J* = 9.3 Hz), 117.79, 115.70, 115.52, 81.06, 75.11, 73.00, 67.80, 66.85, 61.87, 59.06, 20.95, 20.84, 20.78; ^19^F NMR (471 MHz, CDCl_3_) *δ*: -105.18; HRMS (ESI): Calculated forC_27_H_29_ClFN_6_O_10_S [M-H]^-^: 683.13329, found: 683.13519.

*2-(acetoxymethyl)-6-(6-morpholino-9H-purin-9-yl)-5-((4-(trifluoromethoxy)phenyl) sulfonamido)tetrahydro-2H-pyran-3,4-diyl diacetate* (**C33**). White solid; m. p. 125.7-126.4 °C; yield 64.3%; ^1^H NMR (500 MHz, CDCl_3_) *δ*: 8.22 (s, 1H, purine-8-H), 7.80 (s, 1H, purine-2-H), 7.27 (d, *J* = 8.8 Hz, 2H, Ar-2,6-H), 6.86 (d, *J* = 8.4 Hz, 2H, Ar-3,5-H), 6.43 (d, *J* = 8.9 Hz, 1H, -SO_2_NH-), 5.76 (d, *J* = 9.8 Hz, 1H, 6’-H), 5.27 (dd, *J* = 22.2, 9.8 Hz, 2H, -CH_2_-), 4.29 - 4.11 (m, 5H, 4’-H, morpholine-2,6-H), 4.04 (d, *J* = 10.9 Hz, 1H, 3’-H), 3.95 - 3.91 (m, 1H, 2’-H), 3.77 (s, 4H, morpholine-3,5-H), 2.73 (s, 1H, 5’-H), 2.02 (d, *J* = 6.5 Hz, 6H, -COCH_3_), 1.96 (s, 3H, -COCH_3_); ^13^C NMR (125 MHz, ) *δ*: 171.62, 170.69, 169.51, 153.56, 152.48, 151.44, 150.42, 139.06, 135.75, 127.82, 121.19, 120.44, 119.15, 81.06, 74.93, 73.27, 67.85, 66.96, 61.82, 58.69, 20.75, 20.71; ^19^F NMR (471 MHz, CDCl3) *δ*: -57.48; HRMS (ESI): Calculated for C_28_H_30_F_3_N_6_O_11_S [M-H]^-^: 715.16399, found: 715.16620.

*2-(acetoxymethyl)-6-(2-fluoro-6-morpholino-9H-purin-9-yl)-5-((4-(trifluoromethoxy)phenyl)sulfonamido)tetrahydro-2H-pyran-3,4-diyl diacetate* (**C34**). White solid; m. p. 121.3-122.1 °C; yield 67.4%; ^1^H NMR (400 MHz, CDCl_3_) *δ*: 7.75 (d, *J* = 2.7 Hz, 1H, purine-8-H), 7.35 (dd, *J* = 8.7, 2.0 Hz, 2H, Ar-2,6-H), 6.93 (d, *J* = 7.0 Hz, 2H, Ar-3,5-H), 6.50 (d, *J* = 8.7 Hz, 1H, -SO_2_NH-), 5.70 (d, *J* = 7.3 Hz, 1H, 6’-H), 5.35 (td, *J* = 9.8, 2.9 Hz, 1H, -CH_2_-), 5.22 (td, *J* = 9.8, 2.6 Hz, 1H, -CH_2_-), 4.64 (s, 1H, 4’-H), 4.31 - 4.04 (m, 4H, morpholine-2,6-H), 3.96 - 3.92 (m, 1H, 3’-H), 3.74 (s, 5H, 2’-H, morpholine-3,5-H), 2.57 (s, 1H, 5’-H), 2.02 (d, *J* = 3.9 Hz, 6H, -COCH_3_), 1.96 (d, *J* = 2.1 Hz, 3H, -COCH_3_); ^13^C NMR (100 MHz, CDCl_3_) *δ*: 171.68, 170.67, 169.56, 159.66, 157.61, 154.47, 154.27, 151.99 (d, J = 19.0 Hz), 151.36, 139.26, 135.93, 127.77, 121.39, 120.45, 118.81, 117.24, 80.97, 74.81, 72.98, 67.81, 66.76, 61.81, 58.74, 20.68.; ^19^F NMR (470 MHz, ) *δ*: ^19^F NMR (375 MHz, CDCl3) *δ*: -50.19, -57.59; HRMS (ESI): Calculated for C_28_H_29_F_4_N_6_O_11_S [M-H]^+^: 733.15457, found: 733.15674.

*2-(acetoxymethyl)-6-(6-morpholino-9H-purin-9-yl)-5-((3-(trifluoromethoxy)phenyl) sulfonamido)tetrahydro-2H-pyran-3,4-diyl diacetate* (**C35**). White solid; m. p. 105.6-106.4 °C; yield 68.3%; ^1^H NMR (400 MHz, CDCl_3_) *δ*: 8.27 (s, 1H, purine-8-H), 7.82 (s, 1H, purine-2-H), 7.25 (dt, *J* = 7.3, 1.6 Hz, 1H, Ar-2-H), 7.20 - 7.13 (m, 3H, Ar-4,5,6-H), 6.57 (s, 1H, -SO_2_NH-), 5.81 (d, *J* = 9.8 Hz, 1H, 6’-H), 5.39 - 5.29 (m, 2H, -CH_2_-), 4.31 - 4.08 (m, 6H, 4’,3’-H, morpholine-2,6-H), 4.00 - 3.96 (m, 1H, 2’-H), 3.85 (t, *J* = 4.7 Hz, 4H, morpholine-3,5-H), 2.40 (d, *J* = 7.3 Hz, 1H, 5’-H), 2.08 (s, 3H, -COCH_3_), 2.07 (s, 3H, -COCH_3_), 2.04 (s, 3H, -COCH_3_); ^13^C NMR (100 MHz, CDCl_3_) *δ*: 171.47, 170.61, 169.41, 153.62, 152.40, 150.29, 148.61, 142.57, 135.51, 130.10, 124.36, 123.96, 121.38, 119.15, 118.81, 118.33, 81.01, 75.01, 73.14, 67.72, 67.01, 61.70, 58.69, 20.70, 20.62; ^19^F NMR (376 MHz, CDCl_3_) *δ*: -57.83; HRMS (ESI): Calculated for C_28_H_32_F_3_N_6_O_11_S [M+H]^+^: 717.17964, found: 717.17859.

*N-(2-(2-chloro-6-morpholino-9H-purin-9-yl)-4,5-dihydroxy-6-(hydroxymethyl) tetrahydro-2H-pyran-3-yl)-4-methylbenzenesulfonamide* (**C36**). White solid; m. p. 245.3-246.2 °C; yield 62.1%; ^1^H NMR (400 MHz, MeOD) *δ*: 8.03 (s, 1H, purine-8-H), 7.16 (d, *J* = 8.3 Hz, 2H, Ar-2,6-H), 6.87 (d, *J* = 8.1 Hz, 2H, Ar-3,5-H), 5.39 (d, *J* = 9.8 Hz, 1H, -SO_2_NH-), 5.36 (d, *J* = 9.8 Hz, 1H, 6’-H), 4.21 - 3.84 (m, 4H, morpholine-2,6-H), 3.73 (dd, *J* = 15.6, 3.3 Hz, 5H, 4’-H, morpholine-3,5-H), 3.60 (dd, *J* = 12.2, 5.3 Hz, 1H, 3’-H), 3.49 - 3.43 (m, 2H, -CH_2_-), 3.41 - 3.35 (m, 1H, 2’-H), 3.21 (dd, *J* = 3.2, 1.6 Hz, 1H, 5’-H), 2.19 (s, 3H, Ar-CH_3_); ^13^C NMR (100 MHz, MeOD) *δ*: 153.36, 153.14, 151.57, 141.99, 139.04, 138.32, 128.34, 125.05, 117.79, 81.91, 79.53, 75.37, 69.79, 66.48, 60.87, 59.08, 53.47, 20.30; HRMS (ESI): Calculated for C_22_H_26_ClN_6_O_7_S [M-H]^-^: 553.12667, found: 553.12823.

*N-(4,5-dihydroxy-6-(hydroxymethyl)-2-(6-morpholino-9H-purin-9-yl)tetrahydro-2H-pyran-3-yl)-4-nitrobenzenesulfonamide* (**C37**). White solid; m. p. 212.3-213.1 °C; yield 59.4%; ^1^H NMR (400 MHz, MeOD) *δ*: 8.03 (d, *J* = 11.1 Hz, 2H, purine-2,8-H), 7.89 - 7.82 (m, 2H, Ar-3,5-H), 7.47 - 7.42 (m, 2H, Ar-2,6-H), 5.43 (d, *J* = 9.8 Hz, 1H, -SO_2_NH-), 3.99 (s, 4H, morpholine-2,6-H), 3.73 - 3.62 (m, 5H, 6’-H, morpholine-3,5-H), 3.48 (dd, *J* = 6.3, 3.3 Hz, 2H, -CH_2_-), 3.41 - 3.37 (m, 1H, 4’-H), 3.21 (dt, *J* = 3.2, 1.6 Hz, 3H, 3’,2’,5’-H); ^13^C NMR (100 MHz, MeOD) *δ*: 153.24, 151.61, 150.04, 148.66, 147.40, 137.89, 126.32, 122.95, 118.81, 81.90, 79.76, 75.53, 69.83, 66.28, 64.72, 60.89, 59.29; HRMS (ESI): Calculated for C_21_H_24_N_7_O_9_S [M-H]^-^: 550.13507, found: 550.13647.

*N-(2-(2-fluoro-6-morpholino-9H-purin-9-yl)-4,5-dihydroxy-6-(hydroxymethyl) tetrahydro-2H-pyran-3-yl)-4-(trifluoromethyl)benzenesulfonamide* (**C38**). White solid; m. p. 150.3-151.2 °C; yield 68.2%; ^1^H NMR (400 MHz, MeOD) *δ*: 8.10 (s, 1H, purine-8-H), 7.59 - 7.55 (m, 2H, Ar-2,6-H), 7.15 - 7.11 (m, 2H, Ar-3,5-H), 5.45 (d, *J* = 9.8 Hz, 1H, -SO_2_NH-), 3.97 (s, 5H, 6’-H, morpholine-2,6-H), 3.89 - 3.63 (m, 7H, 4’-H, -CH_2_-, mrpholine-3,5-H), 3.55 - 3.47 (m, 3H, 3’,2’,5’-H); ^13^C NMR (100 MHz, MeOD) *δ*: 159.53, 157.48, 154.46, 154.27, 152.27, 152.09, 150.88, 140.81, 138.31, 127.89, 121.55, 120.07, 117.09, 82.24, 79.70, 75.40, 69.89, 66.35, 60.90, 59.05; ^19^F NMR (376 MHz, CD_3_OD) *δ*: -53.25, -59.16; HRMS (ESI): Calculated for C_22_H_23_F_4_N_6_O_8_S [M-H]^-^: 607.12287, found: 607.12445.

## 1.4 ^1^H NMR, ^13^C NMR, ^19^F NMR and HRMS of target compounds C1-C38.

**Fig**. **S1**. ^1^H NMR for target compound **C1**

**Fig**. **S2**. ^13^C NMR for target compound **C1**

**Fig**. **S3**. HRMS for target compound **C1**

**Fig**. **S4**. ^1^H NMR for target compound **C2**

**Fig**. **S5**. ^13^C NMR for target compound **C2**

**Fig**. **S6**. HRMS for target compound **C2**

**Fig**. **S7**. ^1^H NMR for target compound **C3**

**Fig**. **S8**. ^13^C NMR for target compound **C3**

**Fig**. **S9**. ^19^F NMR for target compound **C3**

**Fig**. **S10**. HRMS for target compound **C3**

**Fig**. **S11**. ^1^H NMR for target compound **C4**

**Fig**. **S12**. ^13^C NMR for target compound **C4**

**Fig**. **S13**. HRMS for target compound **C4**

**Fig**. **S14**. ^1^H NMR for target compound **C5**

**Fig**. **S15**. ^13^C NMR for target compound **C5**

**Fig**. **S16**. HRMS for target compound **C5**

**Fig**. **S17**. ^1^H NMR for target compound **C6**

**Fig**. **S18**. ^13^C NMR for target compound **C6**

**Fig**. **S19**. HRMS for target compound **C6**

**Fig**. **S20**. ^1^H NMR for target compound **C7**

**Fig**. **S21**. ^13^C NMR for target compound **C7**

**Fig**. **S22**. HRMS for target compound **C7**

**Fig**. **S23**. ^1^H NMR for target compound **C8**

**Fig**. **S24**. ^13^C NMR for target compound **C8**

**Fig**. **S25**. HRMS for target compound **C8**

**Fig**. **S26**. ^1^H NMR for target compound **C9**

**Fig**. **S27**. ^13^C NMR for target compound **C9**

**Fig**. **S28**. HRMS for target compound **C9**

**Fig**. **S29**. ^1^H NMR for target compound **C10**

**Fig**. **S30**. ^13^C NMR for target compound **C10**

**Fig**. **S31**. HRMS for target compound **C10**

**Fig**. **S32**. ^1^H NMR for target compound **C11**

**Fig**. **S33**. ^13^C NMR for target compound **C11**

**Fig**. **S34**. HRMS for target compound **C11**

**Fig**. **S35**. ^1^H NMR for target compound **C12**

**Fig**. **S36**. ^13^C NMR for target compound **C12**

**Fig**. **S37**. HRMS for target compound **C12**

**Fig**. **S38**. ^1^H NMR for target compound **C13**

**Fig**. **S39**. ^13^C NMR for target compound **C13**

**Fig**. **S40**. ^19^F NMR for target compound **C13**

**Fig**. **S41**. HRMS for target compound **C13**

**Fig**. **S42**. ^1^H NMR for target compound **C14**

**Fig**. **S43**. ^13^C NMR for target compound **C14**

**Fig**. **S44**. HRMS for target compound **C14**

**Fig**. **S45**. ^1^H NMR for target compound **C15**

**Fig**. **S46**. ^13^C NMR for target compound **C15**

**Fig**. **S47**. HRMS for target compound **C15**

**Fig**. **S48**. ^1^H NMR for target compound **C16**

**Fig**. **S49**. ^13^C NMR for target compound **C16**

**Fig**. **S50**. HRMS for target compound **C16**

**Fig**. **S51**. ^1^H NMR for target compound **C17**

**Fig**. **S52**. ^13^C NMR for target compound **C17**

**Fig**. **S53**. HRMS for target compound **C17**

**Fig**. **S54**. ^1^H NMR for target compound **C18**

**Fig**. **S55**. ^13^C NMR for target compound **C18**

**Fig**. **S56**. HRMS for target compound **C18**

**Fig**. **S57**. ^1^H NMR for target compound **C19**

**Fig**. **S58**. ^13^C NMR for target compound **C19**

**Fig**. **S59**. ^19^F NMR for target compound **C19**

**Fig**. **S60**. HRMS for target compound **C19**

**Fig**. **S61**. ^1^H NMR for target compound **C20**

**Fig**. **S62**. ^13^C NMR for target compound **C20**

**Fig**. **S63**. HRMS for target compound **C20**

**Fig**. **S64**. ^1^H NMR for target compound **C21**

**Fig**. **S65**. ^13^C NMR for target compound **C21**

**Fig**. **S66**. HRMS for target compound **C21**

**Fig**. **S67**. ^1^H NMR for target compound **C22**

**Fig**. **S68**. ^13^C NMR for target compound **C22**

**Fig**. **S69**. HRMS for target compound **C22**

**Fig**. **S70**. ^1^H NMR for target compound **C23**

**Fig**. **S71**. ^13^C NMR for target compound **C23**

**Fig**. **S72**. HRMS for target compound **C23**

**Fig**. **S73**. ^1^H NMR for target compound **C24**

**Fig**. **S74**. ^13^C NMR for target compound **C24**

**Fig**. **S75**. HRMS for target compound **C24**

**Fig**. **S76**. ^1^H NMR for target compound **C25**

**Fig**. **S77**. ^13^C NMR for target compound **C25**

**Fig**. **S78**. HRMS for target compound **C25**

**Fig**. **S79**. ^1^H NMR for target compound **C26**

**Fig**. **S80**. ^13^C NMR for target compound **C26**

**Fig**. **S81**. ^19^F NMR for target compound **C26**

**Fig**. **S82**. HRMS for target compound **C26**

**Fig**. **S83**. ^1^H NMR for target compound **C27**

**Fig**. **S84**. ^13^C NMR for target compound **C27**

**Fig**. **S85**. ^19^F NMR for target compound **C27**

**Fig**. **S86**. HRMS for target compound **C27**

**Fig**. **S87**. ^1^H NMR for target compound **C28**

**Fig**. **S88**. ^13^C NMR for target compound **C28**

**Fig**. **S89**. ^19^F NMR for target compound **C28**

**Fig**. **S90**. HRMS for target compound **C28**

**Fig**. **S91**. ^1^H NMR for target compound **C29**

**Fig**. **S92**. ^13^C NMR for target compound **C29**

**Fig**. **S93**. ^19^F NMR for target compound **C29**

**Fig**. **S94**. HRMS for target compound **C29**

**Fig**. **S95**. ^1^H NMR for target compound **C30**

**Fig**. **S96**. ^13^C NMR for target compound **C30**

**Fig**. **S97**. ^19^F NMR for target compound **C30**

**Fig**. **S98**. HRMS for target compound **C30**

**Fig**. **S99**. ^1^H NMR for target compound **C31**

**Fig**. **S100**. ^13^C NMR for target compound **C31**

**Fig**. **S101**. ^19^F NMR for target compound **C31**

**Fig**. **S102**. HRMS for target compound **C31**

**Fig**. **S103**. ^1^H NMR for target compound **C32**

**Fig**. **S104**. ^13^C NMR for target compound **C32**

**Fig**. **S105**. ^19^F NMR for target compound **C32**

**Fig**. **S106**. HRMS for target compound **C32**

**Fig**. **S107**. ^1^H NMR for target compound **C33**

**Fig**. **S108**. ^13^C NMR for target compound **C33**

**Fig**. **S109**. ^19^F NMR for target compound **C33**

**Fig**. **S110**. HRMS for target compound **C33**

**Fig**. **S111**. ^1^H NMR for target compound **C34**

**Fig**. **S112**. ^13^C NMR for target compound **C34**

**Fig**. **S113**. ^19^F NMR for target compound **C34**

**Fig**. **S114**. HRMS for target compound **C34**

**Fig**. **S115**. ^1^H NMR for target compound **C35**

**Fig**. **S116**. ^13^C NMR for target compound **C35**

**Fig**. **S117**. ^19^F NMR for target compound **C35**

**Fig**. **S118**. HRMS for target compound **C35**

**Fig**. **S119**. ^1^H NMR for target compound **C36**

**Fig**. **S120**. ^13^C NMR for target compound **C36**

**Fig**. **S121**. HRMS for target compound **C36**

**Fig**. **S122**. ^1^H NMR for target compound **C37**

**Fig**. **S123**. ^13^C NMR for target compound **C37**

**Fig**. **S124**. HRMS for target compound **C37**

**Fig**. **S125**. ^1^H NMR for target compound **C38**

**Fig**. **S126**. ^13^C NMR for target compound **C38**

**Fig**. **S127**. ^19^F NMR for target compound **C38**

**Fig**. **S128**. HRMS for target compound **C38**

# 2. Antiviral activities against PMMoV

Virions were isolated and extracted from disease-infected leaves of *Nicotiana benthamiana* according to the approach described in the literature (Phytopathology 1967;57:1285). And the curative, protective and inactivating activities of the target compounds against PMMoV were evaluated referenced to the literature as well (J Agric Food Chem 2022;70:6026-6036, J Agric Food Chem 2023;71:7977-7987).

# 3. Mechanism of action

## 3.1 Searching for the target protein of C1 acted on PMMoV

The probe in DMSO (1 *m*M) was added into the virus particle solution and incubated in the dark for 2 h. Then *N*^3^-biotin, BTTAA, CuSO4, and vitamin C were added into the reaction system. The system was reacted at 37 ℃ for 3 h. System analyzed by immunoblotting with antibody of PMMoV CP as loading control, while system analyzed by HRP streptavidin was used as the result of **C2**, **C1** and DMSO with viral total protein.

## 3.2 Determination of the dissociation constants of C1 with PMMoV CP

The dissociation constants of **C1** with PMMoV CP were analyzed by microscale thermophoresis (MST) according to the methods described in the literatures (Biophys J 1998;74:604-615, Bioorg Med Chem Lett 2017;27:4096-4100).

## 3.3 Molecular *modeling*

### 3.3.1 Homology modeling

Homology modeling was performed by Discovery Studio 4.5 to predict the three-dimension (3D) structure of PMMoV CP with TMV CP (PDB code 1EI7) (Biophys J 1998;74(1):604-15, Nucleic Acids Res 2017;45(D1):D271-D281) as a template. The model of PMMoV CP was subjected to energy minimization, and the rationality of protein structure was analyzed through Protein Health (J Agric Food Chem 2009;57(16):7348-55) in Discovery Studio 4.5 and MolProbity Ramachandran analysis in MolProbity (<http://altmolprobity.biochem.duke.edu/>) (Proteins 2003;50(3):437-50, Structure 2011;19(10):1395-412).

Protein Health demonstrated that residues of PMMoV CP in allowed region were 94.8%, residues in marginal region were 4.2%, and residues in disallowed region were 1.0%; The MolProbity Ramachandran analysis (Fig. S133) revealed that 92.3% (286/310) of all residues were in favored (98%) regions, 96.8% (300/310) of all residues were in allowed (> 99.8%) regions. From the results of Protein Health and MolProbity Ramachandran analysis, we can see that the homology modeling of PMMoV CP structure was generally reasonable, as the vast majority of residues were located in favorable or permissible regions.

### 3.3.2 Molecular docking

Molecular docking was performed with reference to the methods described in the literature (Brief Bioinform 2020;21(1):318-328). The 3D structures of compounds were intended by SYBYL 2.0 followed by 2,000-steps steepest descent minimization and 2,000-sreps conjugate gradient minimization, respectively. PMMoV CP was obtained by above mentioned homology modeling, and the hydrogens of the PMMoV CP were added by Discovery Studio 4.5. Compounds were docked into the binding pocket of PMMoV CP through AutoDock 4.2 (Expert Opin Drug Discov 2010;5(6):597-607). The grid was set to be 60 × 60 × 60 with a default spacing 0.375 Å. The genetic algorithm (GA) was used to perform 256 runs. Conformation with top rank was chosen as the representative for further analysis.

### 3.3.3 Molecular Dynamics (MD) Simulations

In order to obtain the stable binding mode of compounds with PMMoV CP, MD simulations were performed based on the docking results of ligands and proteins, respectively. AM1-BCC charge model in Antechamber module of Amber22 program was used to assign charges for ligand atoms (J Comput Chem 2002;23:1623-41, J Comput Chem 2000;21:132-146). The topology and coordinate files of the complex were built with the Leap module in Amber22 program. AMBER ff14SB force field for amino acid residues, and general AMBER force field (GAFF) for ligands (J Chem Theory Comput 2015;11(8):3696-713, J Chem Theory Comput 2006;2:209-15, J Comput Chem 2004;25(9):1157-74), Cl^-^ or Na^+^ for neutralizing the system. All molecules were dissolved in a rectangular TIP3P aqueous cassette extending at least 10 Å in each direction from the solute (J Chem Phys 2004;121(20):10096-103, J Chem Phys 1983;79:926-935). The Cpptraj module in Amber22 was used to perform RMSD, RMSF and radius of gyration (Rg) analysis for measuring the stability of the complex during the MD process.

## 3.4 Construction of infectious clones

Infectious cDNA clones of pCB-GFP-PMMoV CP^Y13A^ and pCB-GFP-PMMoV CP^Y140A^ were constructed followed the approach described in the literature (Methods Mol Biol 2014;1236:241-254). The sequences of the primers shown in Table S3 in details.

## 3.5 Transient expression of agrobacterium tumefaciens

Plasmids of pCB-GFP-PMMoV CP^Y13A^ and pCB-GFP-PMMoV CP^Y140A^ were transformed into *A*. *tumefaciens* EHA105 strain and cultured at 28 ℃ for 36-48 h. Monoclone in liquid medium containing antibiotics (Kan and Rif) was incubated at 28 ℃, 200 rpm for 12-16 h, collected at 5000 rpm, 10 min, and washed with sterile ddH_2_O for three times at 5000 rpm, 10 min. Then the OD_600_ was adjusted to 0.01 with *A*. *tumefaciens* transient transfer solution (10 mM MgCl_2_, 10 mM MES and 200 *μ*M acetosyringone, pH 5.6), and immersed for 4 h or more at rt. Finally, leaves with significant characteristics were collected and stored at -80 ℃ for further western-blot (PLoS Pathog 2020;16:e1008780, J Agric Food Chem 2023;71:7977-7987) and RT-qPCR (Mol Plant Pathol 2014;15:907-917, J Agric Food Chem 2023;71:7977-7987). And the sequences of primers used in the qPCR were listed in Table S3.

## 3.6 Co-immunoprecipitation mass spectrometry (Co-IP MS)

### 3.6.1 Vector construction and material preparation

The Flag-tagged vector was digested with KpnI and AscI, and a fusion protein of Flag-PMMoV CP was constructed through homologous recombination. Western-blot was performed to determine the expression of Flag at 24, 48 and 72 hours of post inoculation (hpi). The time point with the highest expression was selected for further protein extraction.

### 3.6.2 Beads binding antibody

Added and mixed 10 *µ*g target antibody with protein A/G beads at rt for 1 h, centrifuged with 500 rpm at 4 ℃ for 1 min, and discarded the supernatant. Then the pre-cooled PBS was used to resuspend beads, centrifuged with 500 rpm at 4 ℃ for 1 min, and discard the supernatant. Repeated the above procedure once.

### 3.6.3 Antibody binding bait protein

Added and mixed total protein with beads overnight at 4 ℃, centrifuged with 500 rpm at 4 ℃ for 1 min, and discarded supernatant. Then 0.5 mL of pre-cooled PBS used to resuspend beads, centrifuge with 500 rpm at 4 ℃ for 1 min, and discard the supernatant. Repeat 4 times. 30 *µ*L of IP lysis buffer, 30 *µ*L of SDS sample buffer (4x) as the IP group, 45 *µ*l of total protein solution, and 15 *µ*L of SDS sample buffer (4x) as the Input group for further western-blot and mass spectrometry.

## 3.7 RNA sequencing (RNA-seq)

RNA-seq was used to analyze the differentially expressed genes (DEGs) in Flag-CP and Flag-CP^Y13A^ treatment groups of *N*. *benthamiana* at 3 dpi.

## 3.8 Split-luciferase complementation assay (LCA)

Designed primers to construct vectors of nluc-PMMoV CP and nluc-PMMoV CP^Y13A^, cluc-A0A248QEL2, cluc-B1PSM0, cluc-I3QHX5, cluc-LOC109221810 and cluc-A2IBL2 by homologous recombination. Extracted plasmids and waited for sequencing confirmation before transforming them into GV3101 (pU19). *A*. *tumefaciens* transient transfer solution was used to have an OD_600_ value of 1.0, then mixed them with equal OD_600_ and equal volume and injected into *N*. *benthamiana* of same growth with nluc:cluc, nluc:cYFP-trap proteins and nYFP-bait protein:cYFP as negative controls. Interaction of trap proteins of A0A248QEL2, B1PSM0, I3QHX5, LOC109221810 and A2IBL2 with bait proteins of PMMoV CP and PMMoV CP^Y13A^ were recorded by UV at 48-72 hpi. The primers involved in LCA were shown in Table S3.

## 3.9 Bimolecular fluorescence complementation (BiFC)

nYFP-PMMoV CP:cYFP-A0A248QEL2, nYFP-PMMoV CP:cYFP-B1PSM0, nYFP-PMMoV CP:cYFP-I3QHX5, nYFP-PMMoV CP:cYFP-LOC109221810, nYFP-PMMoV CP:cYFP-A2IBL2 and nYFP-PMMoV CP^Y13A^:cYFP-A0A248QEL2, nYFP-PMMoV CP^Y13A^:cYFP-B1PSM0, nYFP-PMMoV CP^Y13A^:cYFP-I3QHX5, nYFP-PMMoV CP^Y13A^:cYFP-LOC109221810, nYFP-PMMoV CP^Y13A^:cYFP-A2IBL2 were co-injected into *N*. *benthamiana* with nYFP:cYFP, nYFP:cYFP-trap proteins and nYFP-bait protein:cYFP as negative controls. Interaction of trap proteins of A0A248QEL2, B1PSM0, I3QHX5, LOC109221810 and A2IBL2 with bait proteins of PMMoV CP and PMMoV CP^Y13A^ was observed at 48 hpi via confocal. The primers involved in BiFC were displayed in Table S3.

## 3.10 Virus-induced gene silencing (VIGS)

pTRV2-I3QHX5, pTRV2-PDS and pTRV2 with pTRV1 in equal OD_600_ (OD = 0.5) and equal volumes, were co-expressed into *N*. *benthamiana* of 4-5 leaves. When the positive control (TRV:PDS) showed the symptom of whitening at 10 dpi, RT-qPCR was used to detect the silencing efficiency of I3QHX5 while pCB-GFP-PMMoV of OD_600_ = 0.1 was injected. The expression levels of PMMoV CP were detected via western blot and RT-qPCR of *N*. *benthamiana* injected with pCB-GFP-PMMoV at 8 dpi. The primers involved in VIGS were exhibited in Table S3.

In order to further investigate the specific function of I3QHX5 in virus replication or transmission. Infected clone plasmid of GFP-PMMoV was transformed into the protoplasts of *N. benthamiana* injected with TRV:00 and TRV:I3QHX5. Firstly, protoplasts of TRV:00 and TRV:I3QHX5 were prepared using protoplast isolation and transformation kit (RealTimer, RTU4072). The de-endotoxin-treated GFP-PMMoV plasmids at a concentration of 1500 ng/*µ*L were transfected into the freshly undiluted protoplasts (5×10^5^/mL), and the samples were collected after transfection of 12 and 24 h. Finally, the accumulation of PMMoV RNAs in each protoplast was detected by RT-qPCR.

## 3.11 Cell viability assay

First, protoplast cells of *N. benthamiana* were prepared using the Protoplast Isolation and Transformation Kit (order number: RTU4072) from RealTimer Inc. The protoplast suspension was adjusted to a density of 2 × 10⁵ cells/mL and mixed with different concentrations of **C1** (final concentrations: 5, 10, 20, 50, and 100 µg/mL). Each treatment group was set up in triplicate wells. Simultaneously, a control group containing protoplast culture medium without **C1** and a blank control well without cells were established. All treatment groups were incubated at 25 °C for 24 h in a constant temperature incubator. After incubation, the medium was removed, and the cells were washed three times with phosphate buffer solution (PBS). Then, 0.5% MTT solution was added to each well, and the plates were incubated at 25 °C for another 4 h. Cell survival was calculated using the following formula to assess viability and growth at different **C1** concentrations.

Cell viability = (OD _treatment group_ – OD _blank group_)/ (OD _control group_ – OD _blank group_)

**Fig**. **S129**. Inhibitory activities of **C1** against PMMoV

**Fig**. **S130**. Antiviral activities of **C1** and Ningnanmycin against CMV (A), PVY (B), and TMV (C)

**Fig. S131.** (A) Morphological characteristics of isolated protoplasts; (B) Microscopic evaluation of membrane integrity assessment via trypan blue vital staining after treating protoplasts with 50 *µ*g/mL **C1** for 24 h; (C) Quantitative determination of cytotoxicity effects induced by 50 *µ*g/mL **C1** on isolated protoplasts as evaluated by MTT assay after 24 h of exposure.

**Fig**. **S132**. Identification of PMMoV CP as a direct target of **C2**. (A) System analyzed by immunoblotting with antibody of PMMoV CP as loading control. (B) System analyzed by HRP streptavidin was used as the result of **C2**, **C1** and DMSO with viral total protein.

**Fig**. **S133**. MolProbity Ramachandran analysis of PMMoV CP

**Fig. S134.** Molecular dynamics (MD) simulations and root mean square deviation (RMSD) between **C2** and PMMoV CP.

**Fig. S135.** Searching for key amino acid sites of **C22** acting on PMMoV CP. (A, B) MD simulations and RMSD between **C22** and PMMoV CP, root mean square fluctuation (RMSF) values and radius of gyration of gyration (Rg) of PMMoV CP.

**Fig**. **S136**. RNA-seq of Flag-CP and Flag-CP^Y13A^ in *N*. *benthamiana* at 3dpi. (A-C) Volcano plot, KEGG Enrichment, and Gene Function Classification (GO) of Flag-CP and Flag-CP^Y13A^ in *N*. *benthamiana* at 3dpi.

**Fig**. **S137**. LCA results of PMMoV CP and PMMoV CP^Y13A^ with LOC109221810 and A2IBL2

**Fig**. **S138**. BiFC results of PMMoV CP and PMMoV CP^Y13A^ with LOC109221810 and A2IBL2

**Fig**. **S139**. Symptom differences of TRV:PDS (A), TRV:I3QHX5 (B) and TRV:00 (C). Diluted protoplasts of TRV:00 (D) and TRV:I3QHX5 (E) under microscope. (F) The accumulation level of PMMoV RNAs in protoplasts TRV:00 and TRV:I3QHX5.

**Fig. S140.** (A) Friction inoculation of GFP-PMMoV in TRV:00 and TRV:I3QHX5 plants. (B) Number of infected virions in TRV:00 and TRV:I3QHX5 plants. (C) Accumulation level of PMMoV RNAs in TRV:00 and TRV:I3QHX5 plants.

**Fig. S141.** (A) Plasmid extraction of YFP-PMMoV and YFP-PMMoV CP^Y13A^; (B) Plasmid sequencing of YFP-PMMoV and YFP-PMMoV CP^Y13A^; (C) Expression of YFP-PMMoV and PMMoV CP^Y13A^ in *N. benthamiana*.

**Fig. S142.** (A) Plasmid extraction of the recombinant pET21a-GFP-PMMoV CP^Y13A^. (B) Sequencing verification of pET21a-GFP-PMMoV CP^Y13A^. (C) Phase separation of pET21a-GFP-PMMoV CP^Y13A^ with I3QHX5 *in vitro*. (D) Purification of pET21a-GFP-PMMoV CP^Y13A^. (E) SDS-PAGE analysis of pET21a-GFP-PMMoV CP^Y13A^ after purification. (F) Desalting result of pET21a-GFP-PMMoV CP^Y13A^. (G) SDS-PAGE analysis of pET21a-GFP-PMMoV CP^Y13A^ after desalting.

**Table S1**

Hydrogen bond analysis during the last 10 ns of the MD simulation.

| Systems | H-bonds | Avg. Length (Å) | Avg. Angle (°) | Occupancy |
| --- | --- | --- | --- | --- |
| PMMoV CP-**C1** | **C1**-Y13 | 2.86 | 138.06 | 76.82% |
|  | **C1**-Y140 | 2.90 | 159.31 | 88.52% |
| PMMoV CP^Y13A^-**C1** | **C1**-Y140 | 2.86 | 151.78 | 81.32% |
| PMMoV CP^Y140A^-**C1** | **C1**-N74 | 2.83 | 160.52 | 51.91% |
|  | **C1**-R219 | 2.91 | 158.99 | 24.13% |
| PMMoV CP-**C22** | **C22**-Y13 | 2.79 | 160.87 | 74.93% |
|  | **C22**-Y140 | 2.85 | 157.96 | 86.06% |
|  | **C22**-N74 | 2.83 | 146.34 | 96.85% |

**Table S2**

Binding free energies of proteins with **C1**, **C2**, and **C22.**

| Types | Binding free energies (kcal/mol) | | | | | | |
| --- | --- | --- | --- | --- | --- | --- | --- |
|  | Δ*E_ele_* | Δ*E_VDW_* | Δ*E*^MM^ | Δ*G*^PB^ | Δ*G*^np^ | −*T*Δ*S* | Δ*G*^bind^ |
| PMMoV CP-**C1** | −38.45 | −62.20 | −100.66 | 61.18 | −7.87 | 23.27 | −24.08 |
| PMMoV CP^Y13A^-**C1** | −11.35 | −38.34 | −49.70 | 25.40 | −4.91 | 20.68 | −8.53 |
| PMMoV CP^Y140A^-**C1** | −24.87 | −58.23 | −83.09 | 49.73 | −7.58 | 30.24 | −10.71 |
| PMMoV CP-**C2** | −41.44 | −64.34 | −105.78 | 63.73 | −8.20 | 26.67 | −23.58 |
| PMMoV CP-**C22** | −37.12 | −61.00 | −97.12 | 49.10 | −6.66 | 27.71 | −26.97 |

Δ*E_ele_*, electrostatic energies; Δ*E_VDW_*, van der Waals interaction; Δ*E*_MM_, molecular mechanical (MM) gas-phase binding energy; Δ*G*^PB^, electrostatic contribution; Δ*G*^np^, nonelectrostatic contribution; −*T*Δ*S*, entropic contribution; Δ*G_bind_*, binding free energy.

**Table S3**

Primers used in this study.

| No. | Primes | Sequence (5’-3’) | Usage |
| --- | --- | --- | --- |
| 1 | pCB301-F | gttcatttcatttggagagggtaaatttttcacaatttaacaacaacaac | Primers to  construct infectious cDNA of PMMoV mutants |
| 2 | pCB301-R | accctctccaaatgaaatgaacttcctta |  |
| 3 | pCB-GFP-PMMoV CP^Y13A^-F | gccttaggttctgtatgggccgatccattagagttacaaaa |  |
| 4 | pCB-GFP-PMMoV CP^Y13A^-R | cggcccatacagaacctaaggccactaattgattggca |  |
| 5 | pCB-GFP-PMMoV CP^Y140A^-F | tacaatcaagctctgttcgagagcgcgagtggactcacct |  |
| 6 | pCB-GFP-PMMoV CP^Y140A^-R | tcgaacagagcttgattgtacattcccgtgccacgaacta |  |
| 7 | nluc-CP-F/nluc-CP^Y13A^-F | agaacacgggggacgagctcggtaccatggcttacacagtttccagtgccaa | Primers used to construct vectors of LCA |
| 8 | nluc-CP-R/nluc-CP^Y13A^-R | gggacgcgtacgagatctggtcgacaggagttgtagcccaggtgagtccac |  |
| 9 | cluc-A0A248QEL2-F | tacgcgtcccggggcggtaccatggagactttcttgttcacctta |  |
| 10 | cluc-A0A248QEL2-R | ttgtagtccatttgttggatctcaagctttaggcttgaggg |  |
| 11 | cluc-A2IBL2-F | tcgtacgcgtcccggggcggtaccatggcaccaaaagccgagaa |  |
| 12 | cluc-A2IBL2-R | tgtagtccatttgttggatcttaagagctagtaaatttagtaacagccttggt |  |
| 13 | cluc-B1PSM0-F | acgcgtcccggggcggtaccatggcatccaagtatcatgcatcaag |  |
| 14 | cluc-B1PSM0-R | ttgtagtccatttgttggatctcattcatcgttttgcgtgttctgg |  |
| 15 | cluc-I3QHX5-F | cgtacgcgtcccggggcggtaccatggctctattagtcgagaaaa |  |
| 16 | cluc-I3QHX5-R | ccttgtagtccatttgttggatctcagtacctgtagtgagca |  |
| 17 | cluc- LOC109221810-F | gtacgcgtcccggggcggtaccatggctctccggagctccta |  |
| 18 | cluc- LOC109221810-R | tccttgtagtccatttgttggatcttaaagaccaaaattacatgcctgcacg |  |
| 19 | cYFP-A0A248QEL2-F | acgcacagggtaccatggagactttcttgttcacctt | Primers used to construct vectors of BiFC |
| 20 | cYFP-A0A248QEL2-R | ctcgcctggggatcctcaagctttaggcttgaggg |  |
| 21 | cYFP-B1PSM0-F | gacgcacagggtaccatggcatccaagtatcatgc |  |
| 22 | cYFP-B1PSM0-R | ctcgcctggggatcctcattcatcgttttgcgtgt |  |
| 23 | cYFP-I3QHX5-F | agtcgacgcacagggtaccatggctctattagtcgagaaaac |  |
| 24 | cYFP-I3QHX5-R | ctcgcctggggatcctcagtacctgtagtgagcag |  |
| 25 | cYFP-LOC109221810-F | tcgacgcacagggtaccatggctctccggag |  |
| 26 | cYFP-LOC109221810-R | ttcgagctcgcctggggatccttaaagaccaaaattacatgcct |  |
| 27 | nYFP-CP-F/nYFP-CP^Y13A^-F | agtcgacgcacagggtaccatggcttacacagtttcca |  |
| 28 | nYFP-CP-R/nYFP-CP^Y13^A-F | ctcgcctggggatccctaaggagttgtagcccag |  |
| 29 | cYFP-A2IBL2-F | gacgcacagggtaccatggcaccaaaagccg |  |
| 30 | cYFP-A2IBL2-F | tcgagctcgcctggggatccttaagagctagtaaatttagtaacagcctt |  |
| 31 | pTRV-I3QHX5-F | aggttaccgaattccttgattcatgagggtgttaagg | Primers used to construct vectors of VIGS |
| 32 | pTRV-I3QHX5-R | gagctcggtaccggatcccggcatccgtacaagtt |  |
| 33 | PMMoV CP-R | atggcttacacagtttccagtgcca | Primers used to amplify the genome of PMMoV CP |
| 34 | PMMoV CP-F | ttaaggagttgtagcccaggt |  |
| 35 | qPMMoV CP-F | gacgaggcgggtagatgatg | Primers for RT-qPCR |
| 36 | qPMMoV CP-R | agttgtagcccaggtgagtc |  |
| 37 | actin-F | ggactctggtgatggtgtca |  |
| 38 | actin-R | gaacatgtaaccacgctcgg |  |
| 39 | qA0A5B9Y6Q5-F | atccacaccaaacctccacc |  |
| 40 | qA0A5B9Y6Q5-R | aacgtgtaggacctgcttcg |  |
| 41 | qA0A248QEL2-F | acaaccaaggccaaagtgga |  |
| 42 | qA0A248QEL2-R | ctgaggtgaacccaatgcct |  |
| 43 | qA0A481NUV9-F | caacggtttggcatggcttt |  |
| 44 | qA0A481NUV9-R | agttttctggggccttctcc |  |
| 45 | qA2IBL2-F | gcccgttacaacaagaagcc |  |
| 46 | qA2IBL2-R | cagaaacagcgtgcttagcc |  |
| 47 | qB1PSM0-F | ggtttggcagaagagtgggt |  |
| 48 | qB1PSM0-R | cagagtggatggcggaaaga |  |
| 49 | qI3QHX5-F | cagatgggttttccccgaca |  |
| 50 | qI3QHX5-R | aaaactggggtgtcctgtgg |  |
| 51 | qTRV-I3QHX5-F | tctaccgataatgctgagtt |  |
| 52 | qTRV-I3QHX5-R | acgagtctttccttcatctt |  |
| 53 | qLOC109221810-F | cttggatgcttggaggaggg |  |
| 54 | qLOC109221810-R | gccaagatttgtcgagtggc |  |
| 55 | qQ8H0B4-F | gctaactgagcttctcggca |  |
| 56 | qQ8H0B4-R | gcgaggatgttgtgggagtt |  |
| 57 | qLOC109206267-F | tgtcggtactccagccaaac |  |
| 58 | qLOC109206267-R | gcctgccaattagacgcttg |  |
| 59 | qAFB70993.1-F | ccgatggacttaccagcctt |  |
| 60 | qAFB70993.1-R | ttttctccaacaacacgctcg |  |
| 61 | 21a-I3QHX5-F | gtatcactcatggcctcgagatggctctattagtcgaga | Primers used to construct vectors of pET21a-GFP CP and pET21a-GFP I3QHX5 |
| 62 | 21a-I3QHX5-R | tctcaatgatggtgatgatggtgtcagtacctgtagtgagca |  |
| 63 | 21a-R | ctcgaggccatgagtgatac |  |
| 64 | 21a-F | caccatcatcaccatcattgaga |  |
| 65 | 21a-CP-F | gtatcactcatggcctcgagatggcttacacagtttcca |  |
| 66 | 21a-CP-R | tctcaatgatggtgatgatggtgctaaggagttgtagcccag |  |
